# Global economic burden of depression in 154 countries from 2025 to 2050

**DOI:** 10.1038/s41591-026-04548-7

**Published:** 2026-07-28

**Authors:** Zhong Cao, Yuheng Luo, Lirui Jiao, Wenjin Chen, Klaus Prettner, Michael Kuhn, David E. Bloom, Dean T. Jamison, Till Winfried Bärnighausen, Simiao Chen

**Affiliations:** 1 Heidelberg Institute of Global Health, Faculty of Medicine and University Hospital, Heidelberg University, Heidelberg, Germany; 2 School of Health Policy and Management, Chinese Academy of Medical Sciences & Peking Union Medical College, Beijing, China; 3 Department of Health Policy and Management, Gillings School of Global Public Health, University of North Carolina at Chapel Hill, Chapel Hill, NC USA; 4 School of Population Medicine and Public Health, Chinese Academy of Medical Sciences & Peking Union Medical College, Beijing, China; 5 Vienna University of Economics and Business (WU), Department of Economics, Vienna, Austria; 6 Vienna Institute of Demography, Austrian Academy of Sciences, Vienna, Austria; 7 Harvard T.H. Chan School of Public Health, Harvard University, Boston, MA USA; 8 Department of Epidemiology and Biostatistics and Institute for Global Health Sciences University of California, San Francisco, CA USA

**Keywords:** Depression, Public health, Economics

## Abstract

Depression is a major contributor to the global burden of diseases with substantial socioeconomic consequences. Here, to estimate the economic effects of depression, we developed a macroeconomic model using data from the World Bank and Global Burden of Disease Study 2021. The model estimated the impact of depression on labor force participation, educational attainment and work experience, adjusted for age and sex. The projected global economic burden from 2025 to 2050 is estimated at US$12 trillion (2024 US dollars), representing 0.460% of annual global gross domestic product. When suicide-related deaths attributable to depression are accounted for, based on an assumed 60% attribution rate representing the upper bound of estimates in the literature, the overall economic burden increases to US$14 trillion. The highest economic burdens are observed in the United States and China. Relative to regional gross domestic product, the impact is greatest in North America. Reduced labor force participation and productivity were identified as the predominant drivers of this cost. The macroeconomic burden of depression is substantial and inequitably distributed globally. These findings underscore the importance of addressing depression as a public health and economic concern. Reducing the health burden of depression could yield economic returns by supporting productivity and capital accumulation.

## Abstract (web summary)

A macroeconomic modeling analysis across 154 countries estimates that depression will impose a US$12 trillion global economic burden between 2025 and 2050, equivalent to around 0.5% of the annual global GDP.

## Main

Depression is a leading contributor to the global burden of mental disorders worldwide, as measured by disability-adjusted life years (DALYs)^1,2^. It is characterized by a persistent low mood or loss of interest in previously pleasurable activities, rather than temporary sadness^3^. It was estimated that approximately 322 million people were living with depression globally in 2023 (around 127 million in males and 196 million in females), corresponding to an age-standardized prevalence rate of 3,810 per 100,000 population (3,005 and 4,611 per 100,000 population in males and females, respectively)^1^. The COVID-19 pandemic had further intensified this burden. A study estimated that the global prevalence of depression and anxiety increased by 27.6% and 25.6%, respectively, in the first year of the pandemic^4^. Another meta-analysis including over one million participants also found that 31% of children and adolescents showed depressive symptoms during this period, a substantial rise compared with pre-pandemic levels^5^. The associated DALY rate is projected to rise to 641 per 100,000 population by 2050^6^.

Previous studies consistently show that depression imposes a substantial societal and economic burden in selected countries^7–13^. Evidence from both high- and middle-income countries suggests considerable healthcare costs and productivity losses associated with depression^7,10,12^. However, existing estimates are largely derived from selected countries and specific settings, limiting cross-country comparability. Evidence also remains particularly limited in low- and middle-income countries, where reliable prevalence estimates and other key data are often lacking^13^. Consequently, comparable country-level projections of the burden of depression and their economic consequences are still needed to better understand the global distribution of this burden and to inform policy responses.

The economic burden of depression has been primarily assessed through cost-of-illness (COI) analyses^14–17^, microsimulation models^18,19^, generalized linear models based on cross-sectional survey data^12,20–23^ and the value of a statistical life method^13^ to estimate the economic value of health losses. COI analyses^14–17^ provide intuitive estimates of direct and indirect costs but do not account for broader economic adjustment mechanisms. Microsimulation models^18,19^ allow detailed modeling of individual-level heterogeneity but are often data intensive and limited in scope. Regression-based approaches^12,20–23^ using cross-sectional data are useful for identifying associations between depression and economic outcomes but may not capture dynamic effects on the economy. Value of a statistical life-based approaches^13^ capture the overall welfare loss associated with morbidity and mortality, but do not explicitly model impacts on economic output or production processes. As a result, these approaches do not fully capture the macroeconomic consequences of depression^24,25^. This gap highlights the need for approaches that can capture economy-wide impacts.

To address these limitations, our study applied a macroeconomic model based on economic growth theory, integrating the morbidity attributable to depression as a key factor shaping the buildup of both physical and human capital to estimate the economic burden of 154 countries from 2025 to 2050. The modeling framework has been previously validated in assessments of noncommunicable diseases, road injuries, COVID-19 and key risk factors (for example, air pollution, tobacco use)^26–33^.

## Results

### Economic burden by country

**Fig. 1:**
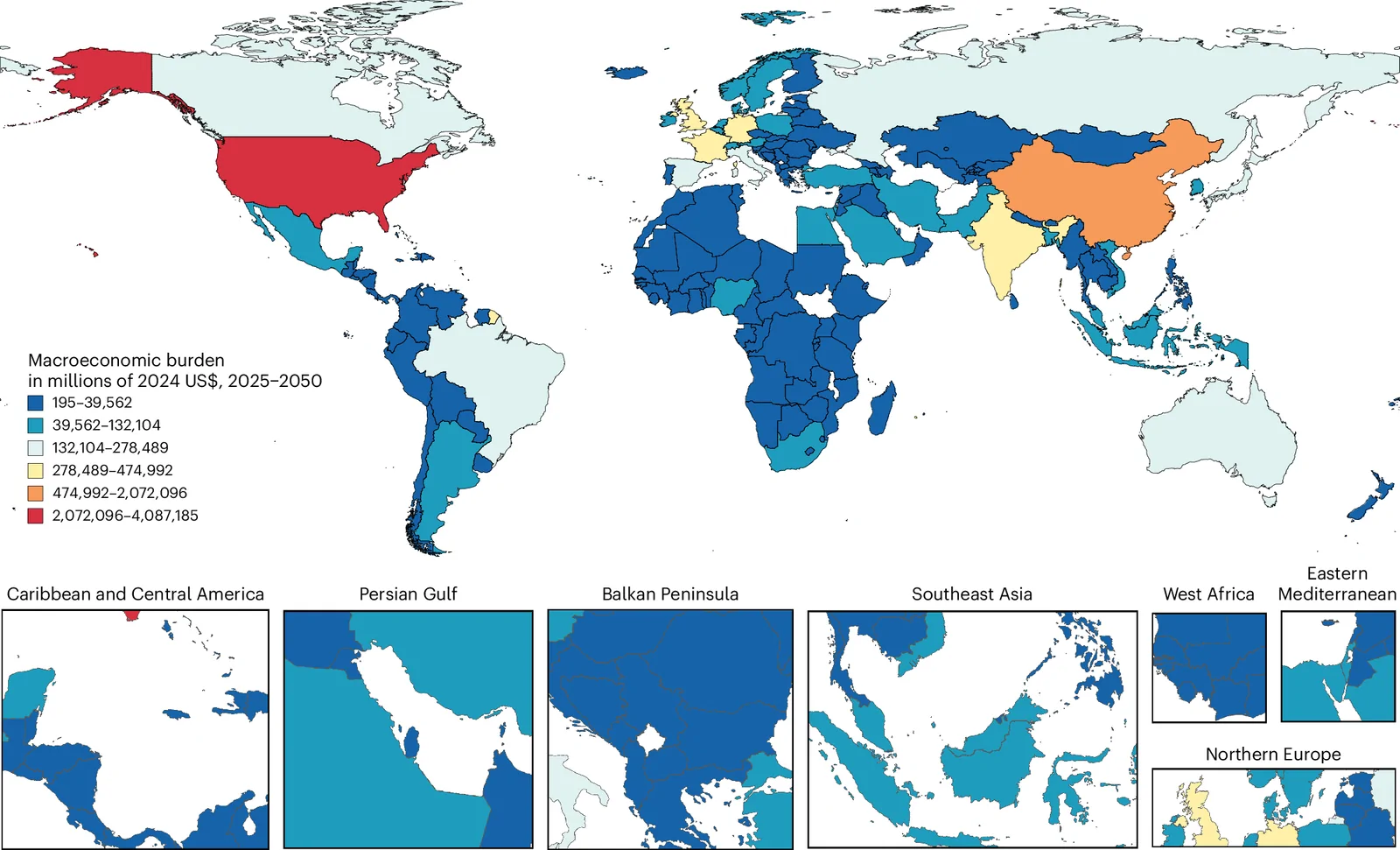
Macroeconomic burden of depression in 2025–2050 (in millions of 2024 US$). The map shows the projected cumulative macroeconomic burden attributable to depression across 154 countries and territories from 2025 to 2050, estimated using a health-augmented macroeconomic model. Economic losses reflect reductions in effective labor supply associated with depression morbidity, together with the effects of treatment-related expenditures on capital accumulation. Countries are shaded according to the magnitude of estimated economic losses, with darker red colours indicating larger cumulative burdens. The highest absolute economic burdens are concentrated in the United States and China, followed by several countries in Europe and East Asia. Regional insets are included to improve visualization of geographically smaller regions and territories. Countries or territories without sufficient data are shown in white. Basemap from Natural Earth (https://www.naturalearthdata.com/). Source data

**Fig. 2:**
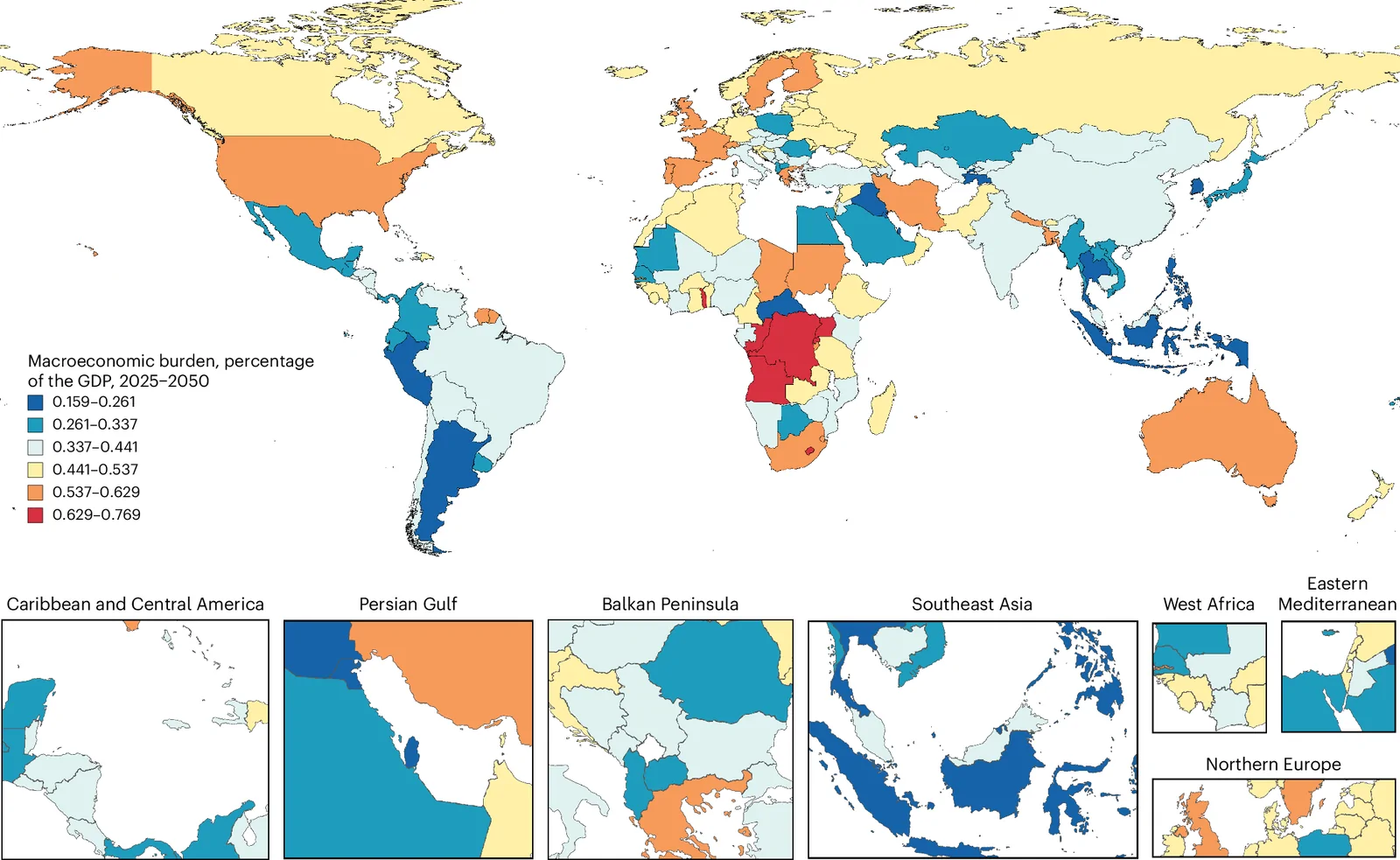
Macroeconomic burden of depression as a percentage of total GDP in 2025–2050. The map shows the projected macroeconomic burden of depression expressed as a percentage of total GDP across 154 countries and territories from 2025 to 2050, based on a health-augmented macroeconomic model. Burden estimates reflect reductions in effective labor supply associated with depression morbidity and the diversion of treatment-related expenditures from savings and investment. Countries are shaded according to burden levels, with darker red colours indicating higher relative economic losses. The highest relative burdens are concentrated in parts of sub-Saharan Africa, North America and Europe, whereas lower relative burdens are observed in parts of East Asia and Latin America. Regional insets are included to improve visualization of geographically smaller regions and territories. Countries or territories without sufficient data are shown in white. Basemap from Natural Earth (https://www.naturalearthdata.com/). Source data

Table 1 presents findings from 154 countries with complete data. The United States bears the highest economic burden attributable to depression, estimated at US$4,087 billion, followed by China (US$2,072 billion) and the United Kingdom (US$475 billion). In terms of gross domestic product (GDP) proportions, Lesotho faces the heaviest economic burden at 0.769%, followed closely by Uganda at 0.751% and Togo at 0.735%. When measured per person, Switzerland shows the highest cost at about US$12,251, slightly above Ireland at US$11,882 and Norway at US$11,685. The geographical distribution of the economic burden and its proportion of GDP are illustrated in Figs. 1 and 2, respectively. Our analysis projects the global macroeconomic burden of depression to be US$12 trillion (95% uncertainty intervals (UI), US$10–15 trillion) between 2025 and 2050, using a 2% discount rate. Our results also imply that the economic burden of depression is equivalent to an annual tax of 0.460% (95% UI, 0.384%–0.551%) on global output, or an average per capita burden of US$1,429 (95% UI, US$1,192–1,710) over the period of 2025–2050. Economic burdens of 154 countries in different years are shown in Supplementary Table 4 in Appendix C.

**Table 1:** Total macroeconomic cost, economic cost as a percentage of GDP in 2025–2050 and per capita cost attributable to depression, by country and World Bank region in 2024 US$ with lower and upper bounds

	Economic cost, 2024 US$ million^a^	Proportion of total GDP (×10^−5^), 2025–2050^a^	Per capita cost, 2024 US$^a^
East Asia and Pacific			
Australia	259,586 (208,173–323,154)	568 (456–707)	8,670 (6,953–10,793)
Brunei Darussalam	814 (626–1,050)	193 (149–249)	1,690 (1,299–2,180)
Cambodia	3,869 (3,029–4,918)	361 (283–459)	193 (151–246)
China	2,072,096 (1,752,053–2,440,645)	367 (311–433)	1,432 (1,211–1,687)
Fiji	430 (337–546)	291 (228–370)	427 (334–542)
Indonesia	106,014 (87,534–127,705)	245 (203–296)	339 (280–409)
Japan	278,489 (237,896–324,869)	291 (249–340)	2,419 (2,066–2,821)
Lao People’s Democratic Republic	1,773 (1,365–2,283)	333 (256–429)	203 (156–261)
Malaysia	50,787 (40,133–63,899)	396 (313–499)	1,339 (1,058–1,685)
Mongolia	2,245 (1,789–2,807)	397 (316–496)	562 (448–703)
Myanmar	6,566 (5,025–8,521)	275 (210–357)	109 (83–142)
New Zealand	28,110 (23,282–33,867)	462 (382–556)	5,253 (4,350–6,328)
Philippines	32,475 (27,125–38,773)	237 (198–283)	246 (205–294)
Republic of Korea	95,322 (79,078–114,655)	239 (198–287)	1,913 (1,587–2,301)
Singapore	22,186 (18,221–26,958)	182 (149–221)	3,490 (2,866–4,241)
Thailand	31,222 (24,754–39,235)	258 (204–324)	452 (359–569)
Vietnam	43,214 (33,519–55,365)	320 (248–410)	406 (315–520)
Europe and Central Asia			
Albania	1,523 (1,189–1,939)	314 (245–399)	572 (447–729)
Armenia	2,565 (2,033–3,224)	416 (330–523)	880 (697–1,106)
Austria	44,783 (36,235–55,201)	413 (334–509)	4,878 (3,947–6,013)
Azerbaijan	7,692 (6,000–9,804)	366 (286–467)	704 (549–898)
Belarus	9,927 (7,870–12,458)	533 (422–669)	1,100 (873–1,381)
Belgium	76,514 (61,541–94,752)	536 (431–663)	6,352 (5,109–7,866)
Bosnia and Herzegovina	2,109 (1,648–2,684)	354 (277–451)	711 (556–905)
Bulgaria	7,886 (6,332–9,793)	352 (282–437)	1,310 (1,052–1,627)
Croatia	7,812 (6,171–9,830)	452 (357–569)	2,118 (1,673–2,665)
Cyprus	3,229 (2,537–4,102)	337 (264–427)	2,468 (1,939–3,135)
Czechia	26,388 (21,161–32,805)	369 (296–459)	2,476 (1,985–3,078)
Denmark	51,322 (40,689–64,509)	523 (414–657)	8,413 (6,670–10,575)
Estonia	4,630 (3,673–5,811)	519 (412–651)	3,753 (2,977–4,710)
Finland	39,562 (32,378–48,171)	574 (470–699)	7,127 (5,833–8,678)
France	389,927 (310,509–487,715)	584 (465–731)	5,803 (4,621–7,258)
Georgia	3,353 (2,642–4,229)	459 (362–579)	899 (709–1,134)
Germany	468,179 (376,364–580,866)	499 (401–620)	5,699 (4,582–7,071)
Greece	26,348 (19,899–34,705)	549 (415–723)	2,744 (2,072–3,614)
Hungary	17,762 (14,058–22,323)	410 (324–515)	1,971 (1,560–2,478)
Iceland	3,204 (2,522–4,047)	485 (381–612)	8,722 (6,863–11,015)
Ireland	64,398 (53,209–77,824)	468 (387–566)	11,882 (9,818–14,359)
Italy	199,940 (162,991–244,223)	439 (358–537)	3,478 (2,836–4,249)
Kazakhstan	21,704 (17,436–26,906)	318 (256–395)	990 (795–1,227)
Kyrgyzstan	1,477 (1,170–1,853)	407 (323–511)	183 (145–229)
Latvia	4,909 (3,948–6,073)	530 (426–656)	3,023 (2,431–3,740)
Lithuania	7,061 (5,611–8,862)	470 (373–589)	3,016 (2,397–3,785)
Luxembourg	8,242 (6,705–10,085)	411 (334–503)	11,302 (9,194–13,829)
Montenegro	584 (458–741)	367 (288–465)	954 (748–1,210)
Netherlands	114,407 (90,807–143,785)	497 (395–625)	6,576 (5,219–8,264)
North Macedonia	1,126 (883–1,430)	333 (261–423)	568 (445–721)
Norway	71,975 (59,032–87,537)	474 (388–576)	11,685 (9,584–14,211)
Poland	55,063 (45,730–65,978)	317 (263–379)	1,546 (1,284–1,852)
Portugal	33,463 (26,046–42,778)	584 (455–747)	3,476 (2,706–4,444)
Republic of Moldova	2,690 (2,128–3,382)	500 (395–628)	728 (576–916)
Romania	21,331 (16,999–26,675)	285 (227–356)	1,216 (969–1,520)
Russian Federation	267,942 (219,758–324,955)	486 (398–589)	1,910 (1,567–2,317)
Serbia	8,039 (6,393–10,061)	393 (313–492)	1,029 (818–1,287)
Slovakia	10,458 (8,267–13,169)	371 (293–467)	1,991 (1,574–2,507)
Slovenia	6,803 (5,411–8,506)	481 (383–602)	3,376 (2,685–4,221)
Spain	200,616 (170,481–235,328)	620 (527–727)	4,421 (3,757–5,186)
Sweden	85,219 (71,419–101,422)	582 (488–692)	7,811 (6,546–9,296)
Switzerland	115,632 (95,488–139,573)	591 (488–713)	12,251 (10,117–14,787)
Tajikistan	930 (730–1,179)	244 (191–309)	70 (55–89)
Turkey	93,020 (72,290–119,190)	359 (279–460)	1,005 (781–1,287)
Ukraine	22,084 (17,759–27,282)	500 (402–618)	570 (458–704)
United Kingdom	474,992 (387,938–579,359)	599 (489–731)	6,605 (5,395–8,057)
Uzbekistan	9,841 (7,754–12,420)	391 (308–494)	248 (195–313)
Latin America and Caribbean			
Argentina	42,819 (35,564–51,419)	252 (209–302)	834 (692–1,001)
Bahamas	1,326 (1,025–1,707)	388 (300–499)	2,996 (2,315–3,858)
Barbados	753 (579–971)	504 (388–650)	2,632 (2,025–3,393)
Belize	387 (302–494)	408 (318–520)	760 (593–970)
Bolivia (Plurinational State of)	5,459 (4,271–6,948)	418 (327–532)	383 (299–487)
Brazil	224,665 (190,509–264,006)	441 (374–518)	991 (840–1,164)
Chile	27,740 (22,023–34,921)	366 (291–461)	1,393 (1,106–1,754)
Colombia	26,786 (21,553–33,114)	296 (238–366)	491 (395–606)
Costa Rica	8,714 (6,762–11,174)	441 (342–565)	1,554 (1,206–1,992)
Dominican Republic	18,039 (13,752–23,502)	516 (393–672)	1,478 (1,127–1,926)
Ecuador	11,002 (8,663–13,941)	324 (255–410)	519 (409–657)
El Salvador	3,842 (2,990–4,914)	420 (327–537)	560 (436–716)
Guatemala	9,778 (7,610–12,547)	298 (232–382)	417 (325–536)
Haiti	2,329 (1,778–3,028)	419 (320–545)	171 (131–223)
Honduras	3,930 (3,016–5,096)	386 (297–501)	317 (243–411)
Jamaica	1,801 (1,383–2,331)	389 (299–504)	594 (456–769)
Mexico	132,104 (109,928–158,303)	311 (259–372)	899 (748–1,078)
Nicaragua	1,865 (1,452–2,386)	402 (313–514)	238 (185–304)
Panama	7,112 (5,690–8,909)	281 (225–352)	1,343 (1,075–1,683)
Paraguay	5,310 (4,158–6,759)	384 (301–489)	631 (494–803)
Peru	10,605 (8,514–13,201)	159 (127–197)	281 (225–349)
Suriname	512 (400–653)	561 (437–714)	785 (612–1,000)
Uruguay	6,368 (5,091–7,947)	329 (263–411)	1,767 (1,413–2,206)
Venezuela (Bolivarian Republic of)	17,435 (13,426–22,511)	396 (305–511)	498 (383–643)
Middle East and North Africa			
Algeria	29,044 (22,148–37,872)	488 (372–637)	535 (408–697)
Bahrain	4,447 (3,525–5,608)	300 (238–378)	2,081 (1,649–2,624)
Djibouti	602 (464–776)	523 (403–674)	507 (390–654)
Egypt	48,389 (38,030–61,439)	297 (233–377)	357 (281–453)
Iran (Islamic Republic of)	59,488 (46,188–76,200)	609 (473–781)	614 (477–787)
Iraq	22,039 (17,668–27,538)	261 (209–326)	380 (305–475)
Israel	80,841 (63,946–102,036)	527 (417–665)	7,348 (5,812–9,274)
Jordan	4,819 (3,760–6,154)	369 (288–472)	416 (325–532)
Kuwait	10,961 (8,903–13,559)	255 (207–315)	2,182 (1,772–2,699)
Lebanon	3,396 (2,549–4,484)	516 (387–681)	535 (402–707)
Malta	1,662 (1,315–2,099)	400 (317–506)	3,768 (2,981–4,759)
Morocco	21,232 (16,470–27,249)	504 (391–647)	493 (383–633)
Oman	20,136 (15,455–26,078)	530 (407–686)	3,197 (2,454–4,141)
Qatar	13,876 (11,146–17,306)	196 (158–245)	3,923 (3,151–4,893)
Saudi Arabia	86,681 (69,587–108,287)	290 (233–362)	2,089 (1,677–2,610)
Syrian Arab Republic	3,910 (2,967–5,111)	465 (353–608)	135 (102–176)
Tunisia	6,897 (5,291–8,951)	537 (412–697)	524 (402–680)
North America			
Canada	251,486 (205,102–307,213)	458 (373–559)	5,885 (4,800–7,189)
United States of America	4,087,185 (3,522,560–4,734,839)	610 (526–707)	11,303 (9,741–13,094)
South Asia			
Bangladesh	82,443 (63,518–106,225)	560 (431–721)	445 (343–574)
Bhutan	472 (365–606)	511 (395–656)	542 (419–697)
India	442,287 (366,898–530,464)	363 (301–435)	283 (235–339)
Maldives	775 (612–979)	346 (273–437)	1,415 (1,116–1,787)
Nepal	7,992 (6,267–10,133)	601 (471–762)	233 (183–296)
Pakistan	58,023 (45,711–73,138)	447 (352–563)	199 (157–251)
Sri Lanka	6,950 (5,474–8,760)	367 (289–463)	315 (248–397)
Sub-Saharan Africa			
Angola	36,387 (27,533–47,728)	723 (547–948)	643 (487–844)
Benin	3,673 (2,818–4,748)	519 (398–671)	195 (150–252)
Botswana	1,944 (1,566–2,413)	293 (236–364)	635 (512–788)
Burkina Faso	3,911 (3,025–5,028)	505 (390–649)	117 (91–151)
Burundi	953 (734–1,230)	559 (430–721)	50 (38–64)
Côte d’Ivoire	10,710 (8,307–13,708)	374 (290–479)	267 (207–341)
Cabo Verde	467 (365–594)	629 (492–801)	730 (571–929)
Cameroon	8,173 (6,308–10,520)	459 (354–591)	204 (158–263)
Central African Republic	195 (155–245)	250 (200–315)	28 (23–36)
Chad	3,027 (2,299–3,959)	573 (435–749)	115 (88–151)
Comoros	224 (173–287)	481 (373–617)	184 (142–236)
Congo	3,999 (3,060–5,191)	720 (551–935)	478 (365–620)
Democratic Republic of the Congo	18,707 (14,161–24,531)	693 (525–909)	127 (96–166)
Eswatini	964 (744–1,242)	574 (443–739)	662 (511–853)
Ethiopia	28,780 (22,988–35,797)	533 (426–663)	172 (137–213)
Gabon	2,653 (2,070–3,390)	362 (282–462)	845 (659–1,080)
Gambia	496 (374–654)	567 (428–748)	130 (98–172)
Ghana	11,988 (9,189–15,527)	513 (393–664)	278 (213–360)
Guinea	4,363 (3,341–5,652)	497 (380–644)	215 (164–278)
Guinea-Bissau	332 (257–428)	508 (392–653)	116 (90–149)
Kenya	20,096 (16,611–24,199)	418 (346–503)	264 (218–318)
Lesotho	564 (437–725)	769 (595–989)	230 (178–295)
Madagascar	2,998 (2,298–3,881)	516 (396–668)	71 (54–92)
Mali	3,283 (2,531–4,231)	380 (293–490)	99 (77–128)
Mauritania	1,440 (1,119–1,843)	330 (257–422)	203 (158–260)
Mauritius	1,217 (957–1,540)	351 (276–444)	976 (768–1,236)
Mozambique	3,927 (3,070–5,016)	423 (331–541)	78 (61–100)
Namibia	1,853 (1,457–2,346)	420 (330–531)	549 (432–695)
Niger	2,651 (2,051–3,411)	360 (278–463)	58 (45–74)
Nigeria	71,421 (57,680–87,898)	385 (311–474)	228 (184–280)
Rwanda	4,275 (3,268–5,552)	735 (562–954)	227 (174–295)
Senegal	4,095 (3,194–5,228)	327 (255–417)	159 (124–202)
Sierra Leone	631 (489–810)	459 (356–589)	58 (45–74)
South Africa	60,869 (50,900–72,455)	542 (453–645)	872 (729–1,038)
Sudan	6,442 (4,862–8,462)	544 (411–715)	99 (75–130)
Togo	2,323 (1,780–3,007)	735 (563–952)	189 (145–245)
Uganda	17,089 (13,053–22,232)	751 (574–977)	242 (185–314)
United Republic of Tanzania	17,783 (13,681–22,952)	517 (398–668)	183 (140–236)
Zambia	6,389 (4,972–8,162)	513 (399–656)	215 (167–274)
Zimbabwe	3,601 (2,750–4,672)	355 (271–461)	180 (137–233)

^a^Uncertainty intervals in parentheses are calculated based on the lower and upper bounds of 95% uncertainty intervals for GBD morbidity data.

### Economic burden by World Bank region and income group

Analysis of the economic burden by World Bank region revealed notable disparities (Table 2). Expressed as a percentage of GDP, the burden was highest in North America (0.599%), followed by Europe and Central Asia (0.506%) and sub-Saharan Africa (0.493%). The East Asia and Pacific regions showed the lowest relative burden (0.351%). The per capita economic burden ranged widely, from US$229 in sub-Saharan Africa to US$10,730 in North America. Consequently, the total aggregate economic burden was greatest in North America (US$4,339 billion) and Europe and Central Asia (US$3,099 billion), while sub-Saharan Africa recorded the lowest (US$375 billion). Stratification by income group indicated that the highest relative burden has materialized in low-income countries (0.566% of GDP), corresponding to an aggregate loss of US$108 billion and US$134 per capita. By contrast, upper-middle-income countries showed the lowest relative burden (0.371% of GDP).

**Table 2:** Total economic cost, cost as a percentage of GDP in 2025–2050 and per capita cost attributable to depression morbidity, by World Bank region and World Bank income group, and globally in billions of 2024 US$ with an upper bound and a lower bound

	Economic costs, 2024 US$ billion^a^	Proportion of total GDP (×10^−5^), 2025–2050^a^	Per capita cost, 2024 US$^a^
By region			
East Asia and Pacific	3,035 (2,544–3,609)	351 (294–418)	1,262 (1,057–1,500)
Europe and Central Asia	3,099 (2,512–3,810)	506 (410–622)	3,354 (2,719–4,124)
Latin America and Caribbean	571 (470–691)	353 (291–428)	802 (661–970)
Middle East and North Africa	418 (329–531)	376 (296–477)	806 (635–1,023)
North America	4,339 (3,728–5,042)	599 (514–696)	10,730 (9,219–12,470)
South Asia	599 (489–730)	391 (319–477)	286 (233–348)
Sub-Saharan Africa	375 (297–471)	493 (390–620)	229 (181–287)
By income group			
Low income	108 (84–139)	566 (438–728)	134 (104–173)
Lower middle income	1,214 (976–1,503)	377 (303–467)	302 (243–374)
Upper middle income	3,205 (2,677–3,823)	371 (310–443)	1,217 (1,017–1,452)
High income	7,891 (6,618–9,396)	528 (443–629)	6,548 (5,492–7,798)
Total	12,435 (10,369–14,884)	460 (384–551)	1,429 (1,192–1,710)

^a^Uncertainty intervals in parentheses are calculated based on the lower and upper bounds of 95% uncertainty intervals for GBD morbidity data.

Extended Data Table 1 reveals a disparity between the geographic distribution of economic losses estimated in our study and the DALYs attributable to depression from the Global Burden of Disease (GBD) study^6^. While depression is projected to result in 51 million DALYs in 2025 and 65 million DALYs in 2050 globally, the economic burden is disproportionately distributed relative to both population size and DALYs. For instance, sub-Saharan Africa, which accounts for 15 million DALYs (22.7%) of the global total in 2050, bears only US$375 billion (3.0%) of the estimated US$12 trillion in economic losses projected for the period 2025–2050. Conversely, Europe and Central Asia, with 7 million DALYs (10.6%) in 2050, accounts for a substantially larger share of the economic burden, totaling US$3 trillion (24.9%) over the same period. East Asia and Pacific face a substantial burden in both health and economic terms, contributing 12 million DALYs (24.5%) of the global total in 2025, 13 million DALYs (20.3%) in 2050 and US$3,035 billion (24.4%) of the overall economic loss. Projections indicate a shift in the distribution of DALYs, with an increasing proportion occurring in low-income and lower-middle-income countries and a corresponding decrease in high-income countries. By 2050, lower-middle-income countries are expected to contribute 47.7% of the global DALYs attributable to depression.

### Roles of physical capital decline in the economic burden of depression

**Fig. 3:**
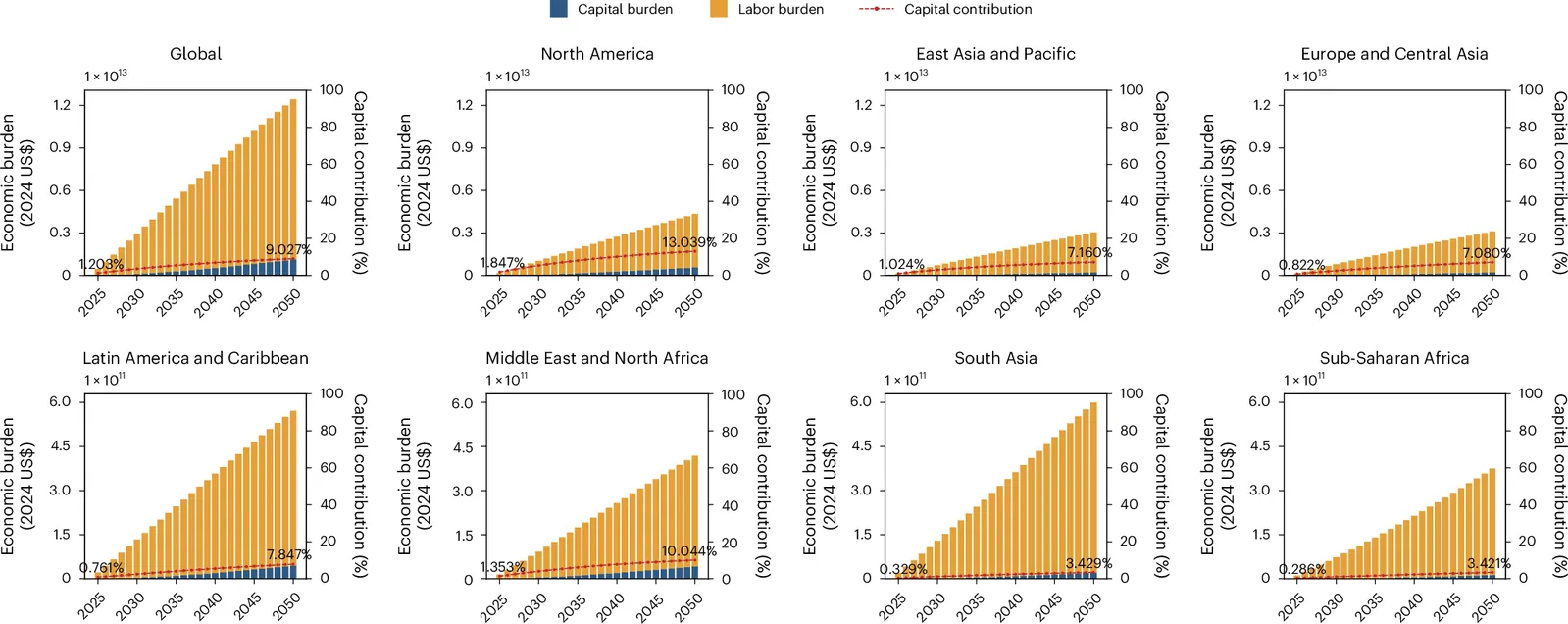
Contribution of physical capital decline and labor force reduction to the total economic cost of depression globally and by World Bank region. The blue charts depict the economic burden attributable to physical capital decline, while the orange charts represent the economic burden associated with labor force reduction. The red lines illustrate the temporal trends in the proportional contribution of physical capital decline to the total economic burden. The cumulative economic burden is adjusted for discounting at a rate of 2% per year. Source data

**Fig. 4:**
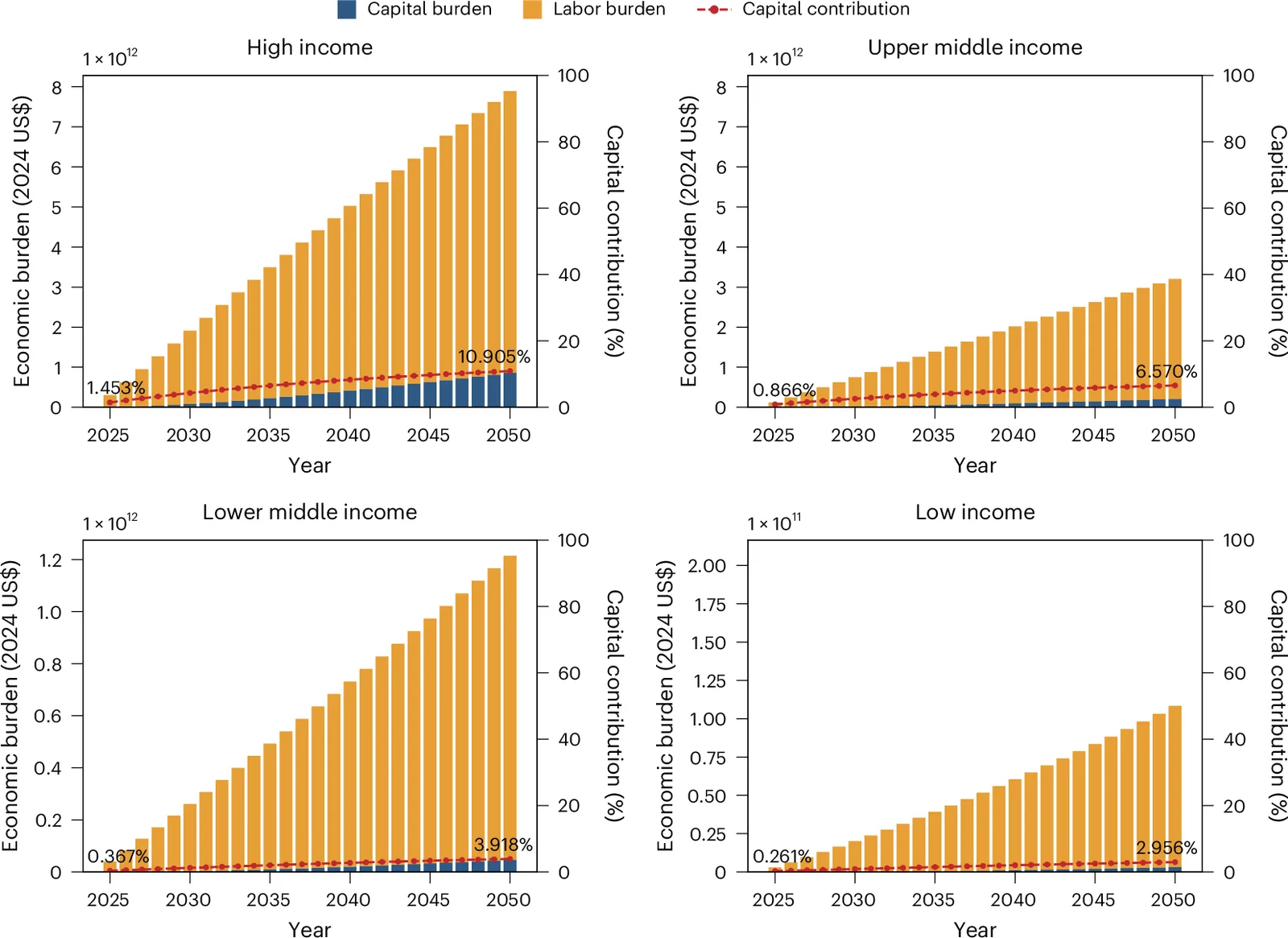
Contribution of physical capital decline and labor force reduction to the total economic cost of depression by World Bank income group. The blue charts depict the economic burden attributable to physical capital decline, while the orange charts represent the economic burden associated with labor force reduction. The red lines illustrate the temporal trends in the proportional contribution of physical capital decline to the total economic burden. The cumulative economic burden is adjusted for discounting at a rate of 2% per year. Source data

Our analysis quantified the proportion of the total economic burden of depression attributable to declines in physical capital from 2025 to 2050. The contribution of physical capital decline was consistently smaller than that of labor force reductions. Globally, the proportion of the total economic burden linked to physical capital decline is projected to increase from 1.203% in 2025 to 9.027% by 2050. Substantial regional heterogeneity was observed (Fig. 3). In 2025, capital contribution is highest in North America at 1.847% and lowest in sub-Saharan Africa at 0.286%. In 2050, North America is projected to have the highest proportion (13.039%), while sub-Saharan Africa is projected to have the lowest (3.421%). Across income groups in 2050 (Fig. 4), high-income countries are projected to experience the highest share (10.905%), followed by upper-middle-income (6.570%), lower-middle-income (3.918%) and low-income countries (2.956%).

### Sensitivity analyses

Supplementary Tables 5 and 6 show the stability of our results by changing all input parameters at once. The results show that the estimated global economic burden of depression dropped slightly to US$10,928 billion, or about US$1,256 per person—figures that are still close to the main estimate of US$12,435 billion and US$1,429 per person. Supplementary Tables 7–10 show additional tests using different discount rates of 0% and 3%. With no discounting (0%), the global economic burden rose sharply to US$16,113 billion, or US$1,852 per person. By contrast, using a 3% rate lowered the total to US$10,997 billion (US$1,264 per person), just below the main estimate based on a 2% rate. Although the absolute numbers shifted under these conditions, the overall pattern stayed the same: the United States, China and the United Kingdom consistently showed the highest economic burdens, while the Central African Republic, Comoros and Guinea-Bissau remained among the lowest.

When the upper bound of GDP was used as an input to the model (Supplementary Tables 11 and 12), the total economic burden amounts to US$13,868 billion, corresponding to 0.457% of GDP. When the lower-bound of GDP was used as an input to the model (Supplementary Tables 13 and 14), the total economic burden is US$11,101 billion, corresponding to 0.462% of GDP. These results are broadly consistent with the baseline estimates. To reflect the substantial cross-country variation in reported treatment costs (Supplementary Table 2), we conducted sensitivity analyses by varying treatment costs. Under the upper-bound scenario, the economic burden reaches 0.832% of GDP (Supplementary Tables 15 and 16), while under the lower-bound scenario, it declines to 0.439% (Supplementary Tables 17 and 18), both of which remain consistent with the baseline estimate.

Incorporating mortality increases the estimated economic burden, but it does not materially alter the overall magnitude of the results. Under the assumption that 60% of suicide deaths are attributable to depression, the economic burden share increases to 0.512%, representing nearly a 10% increase compared with the baseline (Supplementary Tables 19 and 20). Under the assumption that 16% of suicide deaths are attributable to depression, the economic burden share rises only slightly to 0.474% (Supplementary Tables 21 and 22). These findings suggest that, while depression-related mortality contributes to the total burden, morbidity-related productivity losses remain the dominant driver in the model.

## Discussion

This study provides a full global estimate of the economic burden of depression over time using a macroeconomic modeling approach. Our results highlight several important insights. First, we project that depression will result in a cumulative global economic loss of US$12 trillion between 2025 and 2050, equivalent to 0.460% of the total projected cumulative global GDP. Second, this work represents a systematic attempt to estimate the macroeconomic burden of depression across 154 countries, contributing to 96% of the total population. Third, our findings reveal substantial cross-country heterogeneity in both the health and economic consequences of depression. Among the main drivers, lower labor force participation stands out as the primary way depression affects the economy, while the decline in physical capital investment has a smaller role.

Most studies examining the economic burden of depression classify costs into three groups: direct medical, direct nonmedical and indirect costs. Direct medical costs cover spending on healthcare services such as hospital stays, clinic visits and medication. Direct nonmedical costs include things such as transportation and caregiving. Indirect costs arise mainly from lost productivity, when people miss work, struggle to perform at their usual level, or die prematurely^9^. While useful, this traditional framework often overlooks the broader effects depression has on macroeconomic growth over time. Studies have shown that every US$1 invested in scaling up treatment for depression and anxiety yields a return of US$4 in improved health and productivity^34^. Therefore, rather than viewing treatment costs as a drain on economic resources, they should be considered strategic investments that contribute to human capital development and economic growth. Adopting a macroeconomic perspective that accounts for the cumulative effects of depression on human and physical capital formation offers a more accurate estimation of its economic burden. Our perspective mirrors methods used to study the economic effects of other major health issues and highlights why funding mental health is essential for sustainable economic growth^35^.

Our results are broadly comparable to existing evidence. For example, the World Health Organization has estimated that depression and anxiety together cause around US$1.15 trillion (in 2013 USD) in global losses each year (equivalent to about US$1.55 trillion in 2024 USD, roughly 1.040% of global GDP)^34,36^. Given lower per-person costs for anxiety^34,37^ despite comparable prevalence, depression probably accounts for a larger share of the combined economic burden, broadly consistent with our global estimates. Differences between our estimates and previous studies largely reflect variations in methodological approaches (Supplementary Table 23). In China, the estimated direct medical costs^7^ are substantially lower than our burden, as our framework additionally captures broader economic impacts, including the cumulative effects of medical costs on physical capital accumulation. By contrast, the US study using incremental costs yields estimates broadly consistent with ours, suggesting that empirically derived excess costs associated with depression may approximate real-world economic impacts^8^. By comparison, studies applying the human capital approach, such as those from the United States^9^, Romania^10^ and Switzerland^11^, report substantially higher estimates. These approaches directly value lost productivity, particularly due to premature mortality, by multiplying lost work time by wages. By contrast, our model incorporates macroeconomic adjustment mechanisms, whereby labor-market replacement mitigates part of the productivity loss when individuals exit the workforce. As a result, while economic losses remain substantial, they are lower than estimates based purely on forgone earnings.

Previous studies have shown that depression is associated with productivity losses through reduced employment, absenteeism, presenteeism and premature mortality^9,35^. For example, COI studies of treatment-resistant depression and major depression with suicide risk have estimated substantial indirect productivity costs from reduced employment, absenteeism, presenteeism and premature death^38^. Cross-country studies have also shown that depression-related absenteeism and presenteeism costs vary markedly across countries, with presenteeism often accounting for a large share of workplace productivity loss^39^. In addition, systematic reviews have shown that estimates of work impairment and productivity-loss costs associated with depression vary substantially across studies because of differences in study populations, measurement instruments and definitions of productivity loss^40^. This heterogeneity limits their direct use as standardized inputs for long-term macroeconomic projections across 154 countries and territories. Therefore, in this study, years lived with disability (YLDs) were used as standardized indicators of functional health loss that may affect effective labor supply, thereby supporting cross-country comparability within the macroeconomic modeling framework^6,41^. In addition, relatively few previous macroeconomic burden studies of depression have incorporated suicide-related mortality into long-term economic projections. In our sensitivity analyses, inclusion of suicide-related mortality resulted in higher projected macroeconomic losses across regions and income groups, suggesting that the overall economic burden of depression may be underestimated when mortality effects are excluded.

Compared with other major illnesses such as cancer or chronic obstructive pulmonary disease^30,32^, depression stands out for its especially high burden of illness. Several factors help explain this pattern. First, depression affects a large share of the global population. It has been reported that nearly one billion people worldwide live with some form of mental disorder^42^, and about 322 million people experience depression globally in 2023^1^. Second, depression typically emerges early in life^37^. Depression typically appears during adolescence or early adulthood, a stage when people are pursuing education, starting careers and building families. Because it can last for many years, or even a lifetime, its effects accumulate over time. By contrast, many cancers and other major physical illnesses are more often diagnosed later in life. Third, depression makes it hard to work and be productive. The main symptoms of depression, such as low energy, trouble concentrating and loss of motivation, make it hard for a person to do their everyday tasks and work. This leads to substantial losses in productivity and reduced workforce participation. As a result, despite having lower direct mortality rates than cancer or chronic obstructive pulmonary disease, depression still imposes an immense global health burden.

Disparities in the economic burden of nations and regions are evident. For example, Lesotho, Uganda and Togo face the highest economic burden as a percentage of GDP. According to GBD 2021 Forecasting Collaborators^6^, the global age-standardized YLD rate for depression in Lesotho, Uganda and Togo is consistently higher than the global average over the period 2022–2050, which helps explain the relatively high economic burden observed in these countries. Furthermore, high-income countries shoulder much of the depression economic burden. In North America, the overall health burden is particularly high, probably tied to both the widespread presence of mental health conditions and the limits of mental health services. The per-person economic impact is greatest in North America, Europe and Central Asia, which may reflect the higher levels of education and human capital in these regions. In upper-middle-income countries, the burden is lower (around 0.371% of the GDP). One reason for this gap could be the relatively low rate of depression reported in China, whose large population and economy largely influence this income group’s overall figures.

To lessen the global economic impact of depression, we need to work together to make sure that inexpensive, evidence-based therapies are available and that supportive policies are in place. Internet-based cognitive behavioral therapy is one of the most cost-effective solutions, especially in low- and middle-income countries. With low delivery expenses due to digital platforms and less clinician involvement, internet-based cognitive behavioral therapy offers a highly scalable solution that can achieve high benefit-to-cost ratios^43^. Government policy support is also important for reducing the burden of depression. Increasing investment in mental health services, especially through integration into primary care, can extend access, eliminate stigma and promote early intervention, for an estimated yearly cost of only US$1–5 per capita in low-resource countries^44^. Addressing the broader social determinants of mental health and combating stigma can further amplify the effectiveness of these interventions, enhancing their cost-efficiency and creating a sustainable path toward improving global mental health outcomes^45^.

This study has several limitations that warrant consideration. First, while mortality related to suicide is incorporated in sensitivity analyses, our model does not fully capture non-suicide mortality associated with depression. Depression may increase mortality risk through multiple pathways, including comorbid conditions and health-related behaviors. Our estimates should be interpreted as conservative with respect to the broader mortality consequences of depression. Second, while our dataset consisted of 154 countries and territories, representing 96% of the global population, the exclusion of the remaining 4% may limit the generalizability of our findings to some extent. Third, our estimation of treatment costs for depression relied primarily on US-based data scaled by country-specific health expenditure, which may not accurately reflect global treatment patterns. However, given that treatment costs constitute a relatively small proportion of the overall economic burden of depression, this potential bias is unlikely to substantially affect our primary conclusions. Fourth, prevalence estimates were mostly based on household surveys, which are often not nationally representative and rely on self-reported questionnaires rather than clinical diagnosis. Cultural differences in reporting depressive symptoms may introduce measurement bias and affect cross-country comparability. However, the GBD study estimation frameworks apply standardized methods to harmonize these data and remain as a comprehensive source available for global analysis. Fifth, although depression is associated with substantial productivity losses, available productivity estimates remain heterogeneous across countries and study settings. Therefore, YLDs were used as standardized indicators of functional health loss to support cross-country comparability rather than as direct measures of productivity loss.

Overall, depression places a heavy strain on the global economy, accounting for about 0.460% of the world’s annual GDP. This burden, however, is uneven across regions. North America faces the greatest impact at 0.599% of GDP, followed by Europe and Central Asia at 0.506%, and sub-Saharan Africa at 0.493%. These numbers highlight an urgent need to strengthen global efforts aimed at preventing and treating depression, ensuring that investment in mental health becomes a priority to reduce its wide-reaching economic and social costs.

## Methods

### Data sources

This study used officially published and publicly available data from 154 countries and territories. Savings rates were obtained from the World Bank, GDP projections from the GBD Health Financing Collaborator Network^46^ and incidence and prevalence data from the GBD 2021 Forecasting Collaborators^6^. Treatment costs for depression in the United States were derived from a previous study^47^, which systematically estimated national healthcare and public health spending by condition. Based on these official data sources, we aimed to provide an objective estimation of the economic burden of depression. Comprehensive details regarding the data sources for disease, population, labor, education and macroeconomic indicators, along with the parameter values and data sources used in the macroeconomic model, are provided in Appendix A and Supplementary Table 1. To ensure that the results are more accessible, all cost estimates were standardized and expressed in 2024 US dollars.

### Macroeconomic model

We estimated the macroeconomic burden of depression across 154 countries and territories that met our inclusion criteria for complete data availability. Depression was defined according to the standardized classification system used in the GBD study^48^. We directly calculated the macroeconomic burden of depression for 154 countries using the health macroeconomic model described in detail in previous studies and in Appendix B. We calculated all age–sex-specific morbidity rates, education levels, population sizes and labor force participation rates to estimate the economic burden of depression for each country in our model.

In our economic framework, depression imposes substantial economic costs through three interrelated mechanisms that collectively diminish both human and physical capital. First, these disorders reduce the available labor supply mainly through morbidity effects as illness-related productivity declines and absenteeism impair workforce efficiency. Second, depression also slows down the process of building capital. When people struggle with depression, their spending habits often change as they may use savings to cover medical bills that insurance does not fully pay for. At the same time, higher insurance premiums and greater public healthcare spending place pressure on the broader financial system. Together, these factors reduce overall savings and investment. Some of this money simply shifts from general spending to healthcare, but when funds move away from productive investments and into medical costs, the long-term impact can be serious as it weakens both capital growth and productivity, ultimately limiting future economic development. This combined effect on labor and capital highlights how depression affects the economy in ways that go far beyond the immediate cost of treatment. Different from previous studies^26–32^, deaths associated with depression are often recorded under other causes, such as cardiovascular diseases or suicide, rather than being directly attributed to depression itself ^49–51^. This makes it challenging to accurately estimate mortality specifically linked to depression.

Specifically, we estimate the economic burden of depression by translating morbidity into changes in effective labor supply and capital accumulation within a macroeconomic framework. For labor input, we use age–sex-specific prevalence of depression from GBD projections and apply disability weights to adjust effective labor supply. Disability weight represents proportional loss of functional health on a standardized 0–1 scale and is used in the GBD framework to estimate nonfatal health loss^52^. In this study, instead of interpreting them as direct measures of productivity loss, they were used as standardized indicators of reductions in functional health capacity associated with depression, which may affect effective labor supply at the population level within the health-augmented macroeconomic framework. Related approaches have been used in previous burden-of-disease and productivity-loss studies of health conditions, such as depression^38^, uncorrected presbyopia^53^ and myopia^54^. These studies incorporated disability-based measures together with economic or labor-related indicators to estimate the broader health and productivity consequences associated with disease burden. These estimates are combined with country-specific demographics, baseline labor participation rates, human capital, education and working experience to construct effective labor supply across countries. Mortality attributable to depression is not included in the main analysis owing to challenges in depression attribution. To assess its potential impact, we incorporate mortality in sensitivity analyses (Appendix D) by applying alternative assumptions on the proportion of suicide deaths attributable to depression. In addition, treatment costs are incorporated as a reduction in resources available for savings and investment, thereby affecting the trajectory of capital accumulation over time. The magnitude of this effect is determined using country-specific macroeconomic inputs, including GDP, savings rates and health expenditure levels. Details of treatment cost inputs and cross-country extrapolations are provided in Supplementary Table 1.

### Statistical analyses

To estimate the macroeconomic burden of depression, we compared total output (GDP) for the period 2025–2050 under two scenarios: (1) the status quo scenario with no interventions to mitigate morbidity from depression relative to current and projected rates and (2) the counterfactual scenario assuming complete elimination of depression. The macroeconomic burden of depression was then quantified as the cumulative difference in projected annual GDP for these two scenarios. Different from previous studies^26–32^, this study uses prevalence and incidence rates derived from the GBD study^6^ to estimate the average duration time (Supplementary Table 3) of depression. The baseline estimate is computed subject to a yearly discount rate of 2%, and the results are also provided for discount rates of 0% and 3%.

We performed sensitivity analyses by varying morbidity rates across all 154 countries. Baseline estimates were derived using mean morbidity rates from the GBD study. To account for uncertainty, we calculated both lower- and upper-case scenarios using the lower and upper limits of the GBD data. For the main analysis, we used a 2% discount rate. Additional sensitivity analyses were conducted using alternative discount rates (0% and 3%) and variations in key model parameters. As GDP per capita is subject to considerable uncertainty, we re-ran the model using the upper and lower bounds of GDP to assess the differences compared with the baseline results. In addition, because treatment costs vary across regions, we reviewed studies from multiple countries^55–57^ to compare their estimates with those reported in a previous study^47^. To account for this variation and the uncertainty in cost estimates, we incorporated upper and lower bounds of treatment costs into the model as part of sensitivity analyses, allowing us to assess how differences in treatment costs affect the results relative to the baseline.

Notably, the GBD study did not include suicide-related deaths in its estimates of mortality attributable to depression. To account for the potential contribution of mortality, we incorporated suicide deaths as a sensitivity analysis. While global suicide estimates are available^58^, the proportion attributable specifically to depression remains uncertain. Existing literature indicates that approximately 16%–60% of suicide deaths are linked to underlying depression^59–63^. Based on the literature, we applied a range of 16%–60% to total suicide deaths to estimate depression-attributable mortality. These adjusted mortality estimates were then incorporated into the model to assess their impact on the overall economic burden. All sensitivity analysis results are provided in Appendix D.

### Ethics and inclusion statement

This analysis was not submitted for institutional ethical approval because all data used in this study were aggregate level, de-identified and publicly available. No direct human participant recruitment, intervention, primary data collection or access to identifiable individual-level records was involved. The GBD study estimates draw on epidemiological data and statistical modeling from countries and territories worldwide, including low- and middle-income countries. The World Bank, United Nations World Population Prospects, International Labour Organization and Barro Lee Educational Attainment Database provide publicly available national and regional data on economic output, population, labor force participation and educational attainment. These data sources are relevant to countries and regions across diverse geographic and socioeconomic settings. The authorship team jointly contributed expertise in global health, economics, epidemiology and public health. Roles and responsibilities were agreed upon by all authors.

### Reporting summary

Further information on research design is available in the Nature Portfolio Reporting Summary linked to this article.

## Online content

Any methods, additional references, Nature Portfolio reporting summaries, source data, extended data, supplementary information, acknowledgements, peer review information; details of author contributions and competing interests; and statements of data and code availability are available at https://doi.org/10.1038/s41591-026-04548-7.

## Supplementary information

Supplementary Information Supplementary Discussion and Tables 1–23.

Reporting Summary

## Source data

Source Data Figs. 1–4 Statistical source data.

## Data Availability

All data used in this study were obtained from publicly available repositories and databases, including the Global Burden of Disease (GBD) Study database (https://ghdx.healthdata.org/), the World Bank Open Data platform (https://data.worldbank.org/), the United Nations World Population Prospects database (https://population.un.org/wpp/), the International Labour Organization database (https://ilostat.ilo.org/) and the Barro-Lee Educational Attainment Database (http://www.barrolee.com/). Detailed descriptions of the data sources, parameter values and processing procedures are provided in the Methods and Appendix A. No new datasets were generated for this study. Source data are provided with this paper.

## Extended data

**Extended Data Table 1:** Macroeconomic and disease cost measured in DALYs by World Bank region and country income group. The percentages of economic burden and DALYs are calculated with 154 countries with full age-sex specific populations and morbidities. DALYs data are from the GBD Study

	Economic costs, 2024 U.S.$ billion (%)^a^	DALYs in 2025, thousand (%)^a^	DALYs in 2050, thousand (%)^a^	Annual GDP 2025–50, 2024 U.S.$ billion (global %)	Annual population 2025–50, millions (global %)
By World Bank region					
East Asia and Pacific	3035(2544-3609)	12427(8551-17046)	13216(9132-18025)	33238	2406
	24.4% (20.5%-29.0%)	24.5% (16.9%-33.6%)	20.3% (14.0%-27.7%)	32.0%	27.6%
Europe and Central Asia	3099(2512-3810)	6858(4639-9520)	6917(4680-9583)	23567	924
	24.9% (20.2%-30.6%)	13.5% (9.2%-18.8%)	10.6% (7.2%-14.7%)	22.7%	10.6%
Latin America and Caribbean	571(470-691)	4142(2816-5713)	4944(3373-6827)	6215	712
	4.6% (3.8%-5.6%)	8.2% (5.6%-11.3%)	7.6% (5.2%-10.5%)	6.0%	8.2%
Middle East and North Africa	418(329-531)	3665(2392-5254)	4819(3173-6838)	4277	519
	3.4% (2.6%-4.3%)	7.2% (4.7%-10.4%)	7.4% (4.9%-10.5%)	4.1%	6.0%
North America	4339(3728-5042)	3042(2128-4095)	3182(2250-4268)	27873	404
	34.9%(30.0%-40.5%)	6.0% (4.2%-8.1%)	4.9% (3.5%-6.6%)	26.8%	4.6%
South Asia	599(489-730)	13094(8824-18177)	17223(11639-23675)	5892	2097
	4.8% (3.9%-5.9%)	25.8% (17.4%-35.9%)	26.5% (17.9%-36.4%)	5.7%	24.1%
Sub-Saharan Africa	375(297-471)	7469(4858-10543)	14805(9677-20995)	2926	1640
	3.0% (2.4%-3.8%)	14.7% (9.6%-20.8%)	22.7% (14.9%-32.2%)	2.8%	18.8%
By World Bank income group					
Low income	108(84-139)	3857(2447-5574)	7889(5045-11419)	735	806
	0.9% (0.7%-1.1%)	7.6% (4.8%-11.0%)	12.2% (7.8%-17.6%)	0.7%	9.3%
Lower middle income	1214(976-1503)	22197(14897-30891)	30979(20847-42858)	12384	4023
	9.8% (7.9%-12.1%)	43.9% (29.5%-61.1%)	47.7% (32.1%-66.0%)	11.9%	46.2%
Upper middle income	3205(2677-3823)	15587(10634-21468)	16860(11549-23122)	33198	2634
	25.8% (21.6%-30.8%)	30.9% (21.0%-42.5%)	26.0% (17.8%-35.6%)	31.9%	30.3%
High income	7891(6618-9396)	8882(6115-12170)	9206(6380-12562)	57500	1205
	63.5% (53.3%-75.7%)	17.6% (12.1%-24.1%)	14.2% (9.8%-19.3%)	55.3%	13.8%
Total	12435(10369-14884)	50522(34093-70104)	64936(43821-89961)	103987	8702
	100.0%	100.0%	100.0%	100.0%	100.0%

Extended Data Table 1 summarizes the distribution of economic costs attributable to depression morbidity, depression-related DALYs, GDP and population by World Bank region and income group. The results show that economic costs were concentrated in high-income countries and North America, which accounted for 63.5% and 34.9% of the global economic burden, respectively. In contrast, DALYs were concentrated in lower-middle-income countries, South Asia and Sub-Saharan Africa. Lower-middle-income countries accounted for 43.9% of DALYs in 2025 and 47.7% in 2050, despite contributing only 9.8% of global economic costs. These results highlight a mismatch between where the economic costs occur and where the health burden is greatest.

^a^Uncertainty intervals in parentheses are calculated based on the lower and upper bounds of 95% uncertainty intervals for GBD morbidity data.

## Code availability

The code used for the macroeconomic modeling analyses and generation of descriptive tables and figures is publicly available via GitHub at https://github.com/caozhong14/macroeconomic-modelling-depression.
