# Supplementary material for: Global economic burden of depression in 154 countries from 2025 to 2050
Source: Nat Med. 2026 Jul 28;32(9):3406–16. doi: 10.1038/s41591-026-04548-7 (PMC13577905; doi:10.1038/s41591-026-04548-7)
Supplement: Supplementary file 1 [file 41591_2026_4548_MOESM1_ESM.pdf]

nature medicine

Supplementary information https://doi.org/10.1038/s41591-026-04548-7

Global economic burden of depression in 154 countries from 2025 to 2050

In the format provided by the authors and unedited

SI Appendix: Global economic burden of depression in 154 countries from 2025 to 2050

A: Data description 2
Education 2
Morbidity 2
GDP projection 2
Physical capital 2
Labor participation 2
Saving rate and health expenditure 2
Parameter values and data sources 2
Treatment costs 3
Discount rate 3
B: Modeling details 4
Production function 4
Impact of depression on labor supply 5
Impact of depression on physical capital accumulation 10
Decomposing the Economic Burden: Contributions of Physical Capital Accumulation and Effective Labor Supply Dynamics 11
C: Yearly economic burden in 154 cuntries. 11
D: Sensitivity analyses 18
Variation of all parameters 18
Discounted estimates 23
Sensitivity analyses for GDP input data 32
Sensitivity analyses for treatment costs input data 46
Economic burden accounting for suicide-related deaths 61
E: Strengths and limitations 76
References 79

A: Data description

Education

Age-specific educational attainment data were obtained from the Barro-Lee Educational Attainment Database,[18] which provides educational attainment data by five-year age-sex groups up to 2010. For 2010–2030, no age-specific data are available, but the database provides projections for the population aged 15–64. We approximated the age-specific estimates by assuming that educational attainment for each age-sex group grows at the same rate. Because the Barro-Lee database presents data in five-year intervals, linear interpolation was adopted to extend the estimates to each year. For 2030–2050, we projected the education attainment by assuming the same growth rate as between 2010–2030 for each age and sex group.

Morbidity

The age-sex-specific morbidity (measured in YLLs and YLDs) of depression up to 2050 were obtained from the forecasting analysis conducted by GBD 2021.[19,20]

GDP projection

GDP projections for the status quo scenario are from research by the Global Burden of Disease Health Financing Collaborator Network that produced estimates for Gross Domestic Product (GDP) from 1960-2050.[21] Data are provided for GDP in 2020 international dollars and 2022 U.S. dollars. We use the unit of 2022 U.S. dollars and convert it into 2024 U.S. dollars.

Physical capital

For each country, the physical capital stock was obtained from the Penn World Table projections 10.[22] We use the unit of 2017 U.S. dollars and convert it into 2024 U.S. dollars. However, since the data in this database are only updated through 2019, we utilized GDP data (in 2024 U.S. dollars) from Global Burden of Disease Health Financing Collaborator Network for the period 2019 to 2024.

Labor participation

For each country, the labor participation rate (by five-year age-sex groups) were obtained from the International Labour Organization database for 2010–2020.[23] For estimates beyond 2020, we assumed that the percentage growth rate of the labor participation rate remains the same as over 2010–2020.

Population

For each country, the population by five-year age group was obtained from the population dynamics database built by the Department of Economic and Social Affairs (DESA) of the United Nations.[24] For those countries without five-year age group data, we used the total population from DESA for the imputation of the economic burden.

Saving rate and health expenditure

We obtained country-specific saving rates and health expenditures from the World Bank database.[25] For the projection, we assumed that the saving rate remains constant (at the average from 2010–2019), while health expenditures (as a percentage of GDP) grow at the same rate as in 2000–2019. In cases where countries exhibit negative saving rates, we normalize these values to zero for the purpose of labor and capital contribution calculations.

Parameter values and data sources

Table S1 shows parameter values and data sources in the model, where definitions for parameters are taken from Bloom et al. (2020)[1] and Chen et al. (2019).[4,14]

Table S1. Parameter values and data sources

Parameter	**Definition**	Value	Source
α	Capital share	Country specific	Penn World Table[22,26]
δ	Depreciation rate	0.05	Grossmann et al. (2013)[27]
η_1	Mincer elasticity of education	0.091	Psacharopoulos and Patrinos (2018)[28]
η_2	First-degree Mincer elasticity of experience	0.1301	Heckman et al. (2006)[29]
η_3	Second-degree Mincer elasticity of experience	−0.0023	Heckman et al. (2006)[29]
χ_i	Fraction of treatment cost financed out of saving	Set as saving rate	World Bank (2020)[25]

Treatment costs

We estimated treatment costs for depression based on data from Dieleman et al. (2025).[30] For countries with available data, treatment costs per case were calculated using prevalence estimates from the GBD. For countries without direct estimates, costs were extrapolated by assuming that treatment cost per case is proportional to each country's per capita health expenditure, following approaches used in prior studies.[9,31,32] Treatment cost per capita was then obtained by multiplying per-case costs by country-specific prevalence of depression. For years beyond 2020, treatment costs were projected to grow at the same rate as per capita health expenditure in each country. To reflect cross-country variation and assess uncertainty, we also present data from three studies reporting treatment costs in select countries for specific years, allowing comparison with the estimates from Dieleman et al., as shown in **Table S2**.

Table S2. Treatment costs comparison in three countries.

**Country**	**Year**	**Literature result (2024 US dollars)**	**Dieleman result (2024 US dollars)**	**Fraction**
**Spain**[33]	2017	40.504	83.551	0.485
**Japan**[34]	2011	458.078	47.403	9.664
**India**[35]	2019	3.276	1.356	2.417

Discount rate

In the main analysis, we used a discount rate of 2%. Additionally, in the sensitivity analysis, we provided results for alternative discount rates of 0% and 3%.

B: Modeling details

We aimed to quantify the economic impact of depression, accounting for both healthcare expenditures and morbidity-related productivity losses. To quantify the macroeconomic burden of depression, we compared total output (gross domestic product, GDP) for the period 2025-2050 under two scenarios: (i) the status quo scenario with no interventions to reduce mortality and morbidity from depression relative to current and projected rates; and (ii) the counterfactual scenario assuming complete elimination of depression. The macroeconomic burden of depression was then calculated as the cumulative difference in projected annual GDP for these two scenarios. We calculated all age-sex-specific mortality rates, morbidity rates, education levels, population sizes, and labour force participation rates to estimate the economic burden of depression for each country in our model. We also used the disability weight defined by GBD to analyze the impact of depression disability, where "0" means almost no impact on daily life, and "1" represents an impact equivalent to death. For each country, we thus carried out the following analysis:

Step 1. We identified the disease burden of depression (in terms of morbidity, and treatment costs).
Step 2. We constructed economic projections for two scenarios: a status quo scenario, in which GDP is projected to grow based on current estimates and projections of disease prevalence, and a counterfactual scenario, in which depression prevalence is eliminated from the beginning of the time frame. The economic projections utilize a macroeconomic production function and can be further decomposed into two parts:

a) Projections of effective labor supply.
b) Projections of physical capital accumulation.

Step 3. We calculated the economic loss as the discounted (0%, 2%, 3%) cumulative difference in projected annual GDP between these two scenarios.

This detailed model description follows our previous contributions, in which we applied the framework to estimate the economic burden of noncommunicable diseases in China, Japan, and South Korea[1] and in the United States and European countries[2,3] and the economic burden of diabetes, cancer, dementias, chronic obstructive pulmonary disease, road injuries and other risk factors.[3-9]

Production function

Consider an economy in which time $t = 1,2,\dots,\infty$ evolves discretely. Building on Lucas,[10] we considered the following production function for this economy:

$$Y_t = A_t K_t^{\alpha} H_t^{1-\alpha}, \tag{1}$$

where Y_t is aggregate output; A_t is the technological level at time t, which we assume evolves exogenously; K_t is the physical capital stock (i.e., machines, factory buildings, etc.); and H_t represents aggregate human capital. The parameter α is the elasticity of final output with respect to physical capital. The aggregate production function recognizes that output is not only produced with physical capital and raw labor as in the Solow framework,[11] on which the original EPIC model is based,[12] but with effective labor, of which health is a crucial determinant.

Physical capital evolves according to

$$K_{t+1} = (1-\delta)K_t + Y_t - C_t - TC_t = (1-\delta)K_t + \gamma_t Y_t\,, \tag{2}$$

where δ refers to the depreciation rate, γ_t refers to the saving rate, TC_t refers to the costs of ongoing treatment of depression (or the types of depression under consideration), and C_t refers to the volume of consumption. From Equation (2), it follows that the saving rate is defined as

$$\gamma_t = 1 - \frac{C_t + TC_t}{Y_t}. \tag{3}$$

Note that aggregate output Y_t is used for three purposes: (i) to pay treatment costs TC_t (hospitalization, medication, etc.), (ii) to consume C_t, and (iii) to save.

Individuals of age-sex group (a, s) with age a and sex s are endowed with $h_t^{(a,s)}$ units of human capital and supply $\ell_t^{(a,s)}$ units of labor from age 15 up to their retirement. Children younger than 15 and retirees older than the age of R do not work. R varies by country and could correspond to a high age (e.g., some people older than 80 could also be working). In the theoretical derivations, R indicates the upper bound of the summation. In our simulations, we used labor projections from the International Labour Organization and positive values for the labor force exist for cohorts older than 65. We discretized age less than 65 into 13 groups every five years and all ages older than 65 into 1 group. Thus, we have 28 age-sex groups marked as G. Aggregate human capital in the production function (1) is then defined as the sum over the age-sex-time-specific effective labor supply of each age-sex group:

$$H_t = \sum_{(a,s)\in G} h_t^{(a,s)} \ell_t^{(a,s)} N_t^{(a,s)}, \tag{4}$$

where $N_t^{(a,s)}$ denotes the number of individuals in age-sex group (a, s). Note that aggregate human capital increases with the number of working-age individuals who live in the economy (i.e., with a higher $N_t = \sum_{(a,s)\in G} N_t^{(a,s)}$), with individual human capital endowment (i.e., with a higher $h_t^{(a,s)}$ for at least one group), and with labor supply (i.e., with a higher $\ell_t^{(a,s)}$ for at least one group).

We followed Mincer[13] and constructed the average human capital for each age-sex group with age a according to an exponential function of education and work experience:

$$h_t^{(a,s)} = \exp\left[\eta_1\left(ye_t^{(a,s)}\right) + \eta_2\left(a - ye_t^{(a,s)} - 5\right) + \eta_3\left(a - ye_t^{(a,s)} - 5\right)^2\right], \tag{5}$$

where η_1 is the semi-elasticity of human capital with respect to average years of education as given by $ye_t^{(a,s)}$, and η_2 and η_3 are the semi-elasticities of human capital with respect to the experience of the workforce $\left(a - ye_t^{(a,s)} - 5\right)$ and the experience of the workforce squared $\left(a - ye_t^{(a,s)} - 5\right)^2$, respectively. We assumed a school entry age of 5 years throughout.

Impact of depression on labor supply

Following Bloom et al.[1] and Chen et al.,[2,4,5,14] the evolution of labor supply in the status quo scenario is given by

$$L_t^{(a,s)} = \ell_t^{(a,s)} N_t^{(a,s)} \text{ with } N_t^{(a,s)} = \left[1 - \sigma_{t-1}^{(a-1,s)}\right] N_{t-1}^{(a-1,s)}, \tag{6}$$

where $\sigma_t^{(a,s)}$ is the overall mortality rate of the age-sex group with age a and sex s in time t, which is set to zero in the main analysis. Mortality and morbidity reduce effective labor supply. The reduction of the population size $N_t^{(a,s)}$ captures the mortality effect.

Let $\sigma_{r,t}^{(a,s)}$ denote the mortality rate of people in each age-sex group due to depression and let $\sigma_{-r,t}^{(a,s)}$ be the overall mortality rate due to causes other than depression. Then we have

$$\left(1 - \sigma_t^{(a,s)}\right) = \left(1 - \sigma_{r,t}^{(a,s)}\right)\left(1 - \sigma_{-r,t}^{(a,s)}\right). \tag{7}$$

Next, we considered the mortality effect of depression. In general, depression reduces labor supply by reducing the population $N_t^{(a,s)}$ (through $\sigma_{r,t}^{(a,s)}$). In the counterfactual case, where depression is eliminated from time $t = 0$ onward, the evolution of labor supply is defined similarly to equation (6), but with a different overall mortality rate ($\sigma_{-r,t}^{(a,s)}$ instead of $\sigma_t^{(a,s)}$). For simplicity, we assumed that the number of births is the same in both cases at each point in time t.

In the counterfactual scenario, the size of the cohort with age a and sex s at time t ($\overline{N}_t^{(a,s)}$) evolves according to

$$\overline{N}_t^{(a,s)} = \left[1 - \sigma_{-r,t-1}^{(a-1,s)}\right]\overline{N}_{t-1}^{(a-1,s)}, \overline{N}_0^{(a,s)} = N_0^{(a,s)}, \overline{N}_t^{(0,s)} = N_t^{(0,s)}. \tag{8}$$

Following Bloom et al.,[1] the loss of labor due to mortality accumulates over the years according to

$$\overline{N}_t^{(a,s)} = \frac{N_t^{(a,s)}}{\prod_{\tau=0}^{\min\{t,a\}-1}\left[1 - \sigma_{r,t-1-\tau}^{(a-1-\tau,s)}\right]}. \tag{9}$$

The reduction of the labor participation rate $\ell_t^{(a,s)}$ captures the morbidity effect because people with an illness typically reduce their labor supply, either by reducing working hours or by leaving the workforce. Following Bloom et al.,[1] the labor participation rate in the counterfactual scenario $\overline{\ell}_t^{(a,s)}$ can be established as

$$\overline{\ell}_t^{(a,s)} \approx \frac{\ell_t^{(a,s)}}{\prod_{\tau=0}^{\min\{t,a\}-1}\left[1 - p^{\tau} m^{(a-1-\tau,s)}\right]}. \tag{10}$$

where $m^{(a,s)}$ measures the rate of people leaving the labor force due to morbidity of the disease, and p^{τ} is the probability of a patient not recovering from depression morbidity in each year.

The value of p can be inferred from the average duration of the disease through

$$p = 1 - \frac{1}{D}. \tag{11}$$

where D denotes the average duration time of the disease. For a disease, we can estimate its average duration time by

$$P = I * D. \tag{12}$$

where P and I denote the prevalence and incidence rate of the disease, respectively. For depression, we use the prevalence and incidence from GBD data[15], and the average duration of the disease is estimated as 1.06 years. Previous studies have reported the duration of depression. For instance, ten Have et al.[16] estimated that the median duration of depression is 10.7 months, while Herrman et al. [17] reported that most depressive episodes remit within one year. Since our study primarily relies on GBD data, we calculated the global average disease duration by dividing the prevalence rate by the incidence rate of depression in GBD 2023 data. The disease duration estimates for individual countries are presented in **Table S3**. As shown, the shortest duration is observed in the Netherlands, at 0.79 years, while the longest is in Taiwan (province of China), at 1.42 years. Therefore, we consider the use of a global uniform duration of 1.06 years to be comparable with existing evidence, and a reasonable assumption when estimating the economic burden of depression at a global level.

Table S3. Duration of diseases calculated by the fraction of prevalence rate and incidence rate in 204 countries and globally using GBD data.

Location	Duration	Location	Duration	Location	Duration
**Afghanistan**	1.02	**Germany**	1	**Palau**	1.2

**Albania**	1.14	**Ghana**	0.99	**Palestine**	0.95
**Algeria**	1.05	**Greece**	0.9	**Panama**	0.98
**American Samoa**	1.16	**Greenland**	1.06	**Papua New Guinea**	1.06
**Andorra**	1	**Grenada**	1.04	**Paraguay**	0.93
**Angola**	0.94	**Guam**	1.2	**Peru**	1.02
**Antigua and Barbuda**	1.07	**Guatemala**	0.95	**Philippines**	1.21
**Argentina**	0.99	**Guinea**	0.98	**Poland**	1.23
**Armenia**	1.13	**Guinea-Bissau**	0.98	**Portugal**	1.02
**Australia**	0.95	**Guyana**	1.02	**Puerto Rico**	1.12
**Austria**	1.17	**Haiti**	0.88	**Qatar**	1.11
**Azerbaijan**	1.08	**Honduras**	0.94	**Republic of Korea**	0.95
**Bahamas**	1.07	**Hungary**	1.14	**Republic of Moldova**	1.26
**Bahrain**	1.07	**Iceland**	0.91	**Romania**	1.36
**Bangladesh**	1.02	**India**	1.11	**Russian Federation**	1.06
**Barbados**	1.04	**Indonesia**	1.35	**Rwanda**	1.01
**Belarus**	1.15	**Iran (Islamic Republic of)**	1.14	**Saint Kitts and Nevis**	1.03
**Belgium**	0.92	**Iraq**	1.04	**Saint Lucia**	1.05
**Belize**	1.12	**Ireland**	1.02	**Saint Vincent and the Grenadines**	1.01
**Benin**	0.95	**Israel**	0.87	**Samoa**	1.14
**Bermuda**	1.04	**Italy**	1.21	**San Marino**	1.02
**Bhutan**	1.02	**Jamaica**	1.11	**Sao Tome and Principe**	1.08

**Bolivia (Plurinational State of)**	0.98	**Japan**	0.94	**Saudi Arabia**	0.99
**Bosnia and Herzegovina**	1.12	**Jordan**	1.03	**Senegal**	1.02
**Botswana**	1.01	**Kazakhstan**	1.11	**Serbia**	1.15
**Brazil**	0.95	**Kenya**	1.05	**Seychelles**	1.29
**Brunei Darussalam**	0.93	**Kiribati**	1.06	**Sierra Leone**	0.98
**Bulgaria**	1.2	**Kuwait**	1.07	**Singapore**	1.01
**Burkina Faso**	0.95	**Kyrgyzstan**	1.07	**Slovakia**	1.26
**Burundi**	0.96	**Lao People's Democratic Republic**	1.27	**Slovenia**	1.23
**Cabo Verde**	1.13	**Latvia**	1.21	**Solomon Islands**	1.11
**Cambodia**	1.28	**Lebanon**	1.04	**Somalia**	0.97
**Cameroon**	1	**Lesotho**	1.05	**South Africa**	1.08
**Canada**	1.12	**Liberia**	1.01	**South Sudan**	0.95
**Central African Republic**	0.92	**Libya**	1.04	**Spain**	1.01
**Chad**	0.92	**Lithuania**	1.19	**Sri Lanka**	1.24
**Chile**	0.99	**Luxembourg**	0.94	**Sudan**	0.95
**China**	1.16	**Madagascar**	0.97	**Suriname**	1.07
**Colombia**	0.97	**Malawi**	0.94	**Sweden**	0.87
**Comoros**	0.96	**Malaysia**	1.14	**Switzerland**	0.93
**Congo**	0.88	**Maldives**	1.21	**Syrian Arab Republic**	0.97
**Cook Islands**	1.19	**Mali**	1.02	**Taiwan**	1.42
**Costa Rica**	0.88	**Malta**	1.14	**Tajikistan**	1.04

**Croatia**	1.36	**Marshall Islands**	1.12	**Thailand**	1.41
**Cuba**	1.07	**Mauritania**	1.03	**Timor-Leste**	1.14
**Cyprus**	0.95	**Mauritius**	1.02	**Togo**	0.99
**Czechia**	1.21	**Mexico**	1.06	**Tokelau**	1.16
**Côte d'Ivoire**	1.03	**Micronesia (Federated States of)**	1.14	**Tonga**	1.16
**Democratic People's Republic of Korea**	1.08	**Monaco**	1	**Trinidad and Tobago**	1.14
**Democratic Republic of the Congo**	0.94	**Mongolia**	1.09	**Tunisia**	1.05
**Denmark**	0.9	**Montenegro**	1.26	**Turkmenistan**	1.13
**Djibouti**	0.96	**Morocco**	1.04	**Tuvalu**	1.14
**Dominica**	1.02	**Mozambique**	0.98	**Türkiye**	1.06
**Dominican Republic**	1.06	**Myanmar**	1.15	**Uganda**	0.97
**Ecuador**	0.98	**Namibia**	1.06	**Ukraine**	1.03
**Egypt**	1.06	**Nauru**	1.11	**United Arab Emirates**	1.1
**El Salvador**	0.96	**Nepal**	1.06	**United Kingdom**	0.86
**Equatorial Guinea**	0.98	**Netherlands**	0.79	**United Republic of Tanzania**	0.98
**Eritrea**	0.99	**New Zealand**	0.99	**United States Virgin Islands**	1.1
**Estonia**	1.2	**Nicaragua**	0.97	**United States of America**	1.06
**Eswatini**	1.06	**Niger**	0.96	**Uruguay**	1.04
**Ethiopia**	0.91	**Nigeria**	0.97	**Uzbekistan**	1
**Fiji**	1.2	**Niue**	1.2	**Vanuatu**	1.11
**Finland**	1.05	**North Macedonia**	1.27	**Venezuela (Bolivarian Republic of)**	0.97

**France**	0.99	**Northern Mariana Islands**	1.18	**Viet Nam**	1.19
**Gabon**	0.97	**Norway**	1.02	**Yemen**	0.99
**Gambia**	0.97	**Oman**	1.16	**Zambia**	0.97
**Georgia**	1.02	**Pakistan**	0.98	**Zimbabwe**	1.03

Because the impact of morbidity is hard to estimate directly and the mortality for depression cannot be taken from the GBD study, we used the estimation of disability weights (DW) to get morbidity for each age-sex group:

$$m^{(a,s)} = \mathrm{DW} * I^{(a,s)}. \tag{13}$$

where $I^{(a,s)}$ denotes the incidence rate of corresponding age and sex group. Noting that as incidence of depression is harder to estimate than prevalence, so we used equation (12) to obtain incidence data on the basis of prevalence.

In sum, by reducing the prevalence of depression, the *counterfactual scenario* is associated with an increase in labor supply as compared with the *status quo scenario.* We approximated the change in labor supply (at time t for each age-sex group) by

$$\Delta L_t^{(a,s)} \approx \ell_t^{(a,s)} N_t^{(a,s)} \sum_{\tau=0}^{\min\{t,a\}-1} \left[\sigma_{r,t-1-\tau}^{(a-1-\tau,s)} + p^{\tau} m^{(a-1-\tau,s)}\right]. \tag{14}$$

Bloom et al. (2020)[1] provides the detailed mathematical proof.

Impact of depression on physical capital accumulation

Depression also impedes the accumulation of physical capital because savings finance part of the treatment costs. Following Bloom et al.[1] and Chen et al.,[2] physical capital accumulation in the counterfactual scenario can be written as

$$\bar{K}_{t+1} = \bar{\gamma}_t \bar{Y}_t + (1-\delta)\bar{K}_t\,, \tag{15}$$

$$\bar{\gamma}_t \bar{Y}_t = \bar{I}_t = \bar{Y}_t - \bar{C}_t = \gamma_t \bar{Y}_t + \chi TC_t,$$

where an overbar indicates the counterfactual scenario and where χ is the fraction of the treatment cost that is diverted to savings. The counterfactual saving rate is thus defined by

$$\bar{\gamma}_t = \frac{\gamma_t \bar{Y}_t + \chi TC_t}{\bar{Y}_t}. \tag{16}$$

For more details, see Bloom et al. (2020)[1] and Chen et al. (2018).[2]

Because depression is assumed to be eliminated in the counterfactual scenario, the resources that were devoted to its treatment can now be used for savings or for consumption. Notice that this creates an income effect that, in reality, could affect the division of households' income between savings and consumption. For tractability, we assumed that aggregate investment consists of two parts in the counterfactual scenario: a fixed share γ_t of total output and an additional part from TC_t that would otherwise have been used to pay to treat depression:

$$\bar{I}_t = \gamma_t \bar{Y}_t + \chi TC_{t.} \quad (17)$$

Decomposing the Economic Burden: Contributions of Physical Capital Accumulation and Effective Labor Supply Dynamics

From equation (1), taking logarithms on both sides gives:

$$log(Y_t) = log(A_t) + \alpha\, log(K_t) + (1-\alpha)\, log(H_t). \quad (18)$$

In this model, we assume that A_t evolves exogenously ($\bar{A}_t = A_t$), then we have:

$$log(\bar{Y}_t) - log(Y_t) = \alpha(log(\bar{K}_t) - log(K_t)) + (1-\alpha)(log(\bar{H}_t) - log(H_t)) \quad (19)$$

Thus, we can break down the change in $\log(Y_t)$ into two components: one driven by the change in physical capital K_t ($\alpha(log(\bar{K}_t) - log(K_t))$) and the other by the change in human capital or effective labor supply H_t ($(1-\alpha)(log(\bar{H}_t) - log(H_t))$).

The contribution of physical capital to the economic burden is given by:

$$C_{K_t} = \frac{\alpha(log(\bar{K}_t) - log(K_t))}{log(\bar{Y}_t) - log(Y_t)}. \quad (20)$$

Similarly, the contribution of human capital or effective labor supply is:

$$C_{H_t} = \frac{(1-\alpha)(log(\bar{H}_t) - log(H_t))}{log(\bar{Y}_t) - log(Y_t)}. \quad (21)$$

where $C_{K_t}$and C_{H_t} represent contribution of physical capital and human capital at time t respectively.

C: Yearly economic burden in 154 cuntries.

Table S4: Yearly economic burden of 154 countries in millions of 2024 USD, discounting at 2%.

Country	2025	2030	2035	2040	2045	2050
**Albania**	65.175	64.797	59.926	56.304	53.034	48.803
**Algeria**	1005.869	1089.202	1122.143	1151.19	1148.654	1114.447
**Angola**	964.254	1153.59	1322.676	1488.82	1643.609	1792.048
**Argentina**	1475.126	1635.56	1690.603	1700.111	1653.895	1587.395

**Armenia**	98.997	104.521	103.419	100.151	92.326	81.889
**Australia**	9281.008	10074.366	10147.319	10095.608	9955.197	9665.386
**Austria**	1850.515	1880.745	1765.101	1671.894	1581.851	1485.36
**Azerbaijan**	298.445	312.026	306.205	297.037	280.355	257.125
**Bahamas**	51.753	54.938	53.362	50.546	47.288	44.175
**Bahrain**	158.096	177.539	180.371	174.542	164.365	153.455
**Bangladesh**	2697.583	3104.903	3224.712	3279.588	3249.028	3150.852
**Barbados**	33.083	32.744	30.105	27.637	25.425	23.236
**Belarus**	404.993	410.848	399.064	379.706	351.855	317.567
**Belgium**	3068.739	3143.67	3018.738	2893.735	2756.699	2617.262
**Belize**	12.65	14.411	15.075	15.378	15.422	15.278
**Benin**	102.955	121.47	134.484	148.191	161.291	174.588
**Bhutan**	15.617	17.616	18.285	18.726	18.772	18.349
**Bolivia (Plurinational State of)**	181.128	201.386	210.361	216.959	219.272	217.431
**Bosnia and Herzegovina**	87.598	88.941	84.516	79.974	73.034	66.47
**Botswana**	55.343	66.446	73.674	79.487	83.26	84.342
**Brazil**	8686.382	9090.881	8892.768	8637.14	8222.941	7692.683
**Brunei Darussalam**	34.643	35.36	32.943	30.337	27.488	24.32
**Bulgaria**	330.414	344.312	323.014	294.319	264.028	235.848
**Burkina Faso**	101.543	122.978	139.817	158.968	178.625	198.099
**Burundi**	25.587	30.826	34.731	38.755	42.595	46.266
**Côte d'Ivoire**	297.859	352.939	391.998	433.739	471.917	507.122
**Cabo Verde**	15.262	17.375	18.179	18.651	18.604	17.919

**Cambodia**	123.962	141.6	148.286	153.658	157.208	155.88
**Cameroon**	223.114	267.551	301.715	334.278	361.387	384.29
**Canada**	9546.678	9998.012	9879.446	9671.122	9369.044	9006.168
**Central African Republic**	5.01	5.988	6.868	7.913	9.027	10.101
**Chad**	82.978	98.11	110.172	122.32	134.908	148.174
**Chile**	1014.707	1079.37	1090.63	1081.648	1052.294	1008.071
**China**	73618.062	81281.424	81800.226	81211.428	78432.059	74369.203
**Colombia**	1016.023	1074.988	1051.956	1031.331	993.113	939.302
**Comoros**	7.137	8.02	8.471	8.88	9.219	9.452
**Congo**	117.345	137.834	151.34	161.589	169.83	176.609
**Costa Rica**	322.714	347.309	345.842	340.384	323.923	301.089
**Croatia**	349.501	345.557	316.248	287.893	257.111	226.077
**Cyprus**	119.528	126.908	126.456	125.787	122.302	114.429
**Czechia**	1040.146	1107.163	1075.151	1002.327	920.429	851.829
**Democratic Republic of the Congo**	531.295	630.35	698.036	761.008	811.237	846.99
**Denmark**	2073.116	2128.305	2022.296	1919.157	1837.38	1762.833
**Djibouti**	20.594	23.118	23.643	23.873	23.238	22.28
**Dominican Republic**	649.135	705.821	704.439	700.463	686.56	660.491
**Ecuador**	361.552	405.701	426.08	439.576	441.009	434.934
**Egypt**	1392.205	1636.551	1803.893	1973.154	2086.468	2161.963
**El Salvador**	145.16	153.062	151.839	149.216	142.53	133.759
**Estonia**	190.663	196.25	186.527	174.723	161.01	144.966
**Eswatini**	29.261	33.11	36.043	38.919	41.093	42.341

**Ethiopia**	779.321	959.539	1063.861	1162.38	1262.535	1354
**Fiji**	15.706	16.869	16.941	16.871	16.224	15.312
**Finland**	1587.54	1634.641	1576.298	1502.073	1412.487	1315.501
**France**	15775.106	16177.479	15450.679	14658.153	13917.244	13149.576
**Gabon**	80.576	94.388	101.436	106.693	109.993	112.242
**Gambia**	13.666	16.288	18.135	20.069	21.997	23.595
**Georgia**	134.015	137.081	132.668	127.88	120.59	111.842
**Germany**	19476.723	19520.88	18263.096	17540.797	16671.608	15603.944
**Ghana**	359.176	414.398	449.809	483.884	509.293	524.195
**Greece**	1221.176	1190.785	1066.465	953.951	845.615	753.873
**Guatemala**	294.911	343.434	373.188	394.611	408.595	416.832
**Guinea**	117.452	141.06	159.29	177.318	194.618	211.962
**Guinea-Bissau**	9.401	11.058	12.233	13.441	14.547	15.533
**Haiti**	84.977	89.722	90.237	90.752	89.831	87.168
**Honduras**	122.522	141.244	150.621	157.648	161.589	162.649
**Hungary**	736.671	766.163	721.3	661.865	601.154	558.193
**Iceland**	132.654	134.4	127.055	119.775	112.932	105.763
**India**	14549.114	16703.177	17278.397	17512.502	17412.067	17016.471
**Indonesia**	3696.26	4089.433	4127.774	4131.779	4089.289	4013.769
**Iran (Islamic Republic of)**	2298.377	2434.689	2399.423	2314.388	2142.451	1929.674
**Iraq**	600.146	744.733	838.641	903.328	949.897	989.35
**Ireland**	2431.709	2616.747	2586.809	2489.102	2340.854	2190.2
**Israel**	2683.894	3029.889	3148.141	3201.68	3197.155	3152.208
**Italy**	9025.368	8889.834	8065.067	7243.714	6547.136	5985.945
**Jamaica**	73.922	76.269	72.527	67.973	62.825	56.625
**Japan**	12280.036	12356.829	11360.715	10171.416	9147.901	8293.254

**Jordan**	169.902	184.903	187.544	188.593	186.516	182.32
**Kazakhstan**	781.405	837.859	847.759	847.794	835.72	800.515
**Kenya**	569.669	691.274	763.867	818.274	855.501	882.68
**Kuwait**	394.344	446.03	449.029	432.816	399.504	352.598
**Kyrgyzstan**	50.677	54.76	56.561	58.217	58.948	58.36
**Lao People's Democratic Republic**	57.724	64.543	67.291	70.232	72.373	72.618
**Latvia**	218.685	214.655	197.748	180.521	163.298	145.824
**Lebanon**	148.125	142.837	135.314	128.014	118.023	105.352
**Lesotho**	18.435	20.552	21.673	22.481	22.892	23.034
**Lithuania**	314.504	308.05	281.983	258.555	236.464	214.767
**Luxembourg**	321.249	333.73	323.159	313.322	302.506	290.269
**Madagascar**	88.547	103.692	113.126	120.822	127.04	132.45
**Malaysia**	1710.476	1923.306	1984.917	2015.744	1990.292	1917.137
**Maldives**	27.266	29.924	30.702	30.747	29.691	27.562
**Mali**	86.952	104.296	118.321	133.399	148.458	164.065
**Malta**	60.316	65.669	65.719	64.537	62.458	59.62
**Mauritania**	38.662	46.861	53.099	59.008	64.017	68.331
**Mauritius**	48.454	50.103	47.963	46.235	43.593	41.093
**Mexico**	4687.923	5147.933	5221.622	5171.509	5028.768	4823.141
**Mongolia**	75.205	82.792	86.765	89.295	89.496	88.854
**Montenegro**	24.486	24.772	23.309	21.895	20.261	18.537
**Morocco**	736.385	806.373	822.735	834.169	830.068	811.96
**Mozambique**	89.911	116.214	138.5	162.47	186.351	210.598
**Myanmar**	224.071	247.345	252.869	257.98	259.542	258.129
**Namibia**	56.966	65.474	70.319	74.46	77.281	78.939

**Nepal**	257.875	295.452	308.094	317.783	321.701	317.05
**Netherlands**	4682.238	4788.949	4504.875	4260.163	4066.885	3857.36
**New Zealand**	1046.824	1121.453	1109.734	1082.612	1046.907	999.57
**Nicaragua**	65.034	71.713	73.295	73.636	71.957	68.757
**Niger**	60.962	77.109	92.101	108.896	126.971	146.175
**Nigeria**	2147.953	2461.725	2666.116	2857.45	3040.784	3207.212
**North Macedonia**	46.923	48.356	45.638	42.444	38.437	34.193
**Norway**	2762.542	2944.592	2866.823	2741.918	2612.458	2476.69
**Oman**	672.413	755.633	796.706	811.074	790.208	745.954
**Pakistan**	1764.412	2037.343	2193.528	2333.059	2434.736	2489.215
**Panama**	213.241	253.737	274.404	288.23	293.599	294.133
**Paraguay**	175.025	197.765	206.528	212.066	211.45	205.614
**Peru**	357.563	400.731	413.327	420.094	417.451	403.837
**Philippines**	1033.433	1209.03	1264.779	1295.313	1293.906	1276.789
**Poland**	2278.046	2357.733	2242.214	2089.112	1881.854	1657.078
**Portugal**	1492.96	1473.386	1343.974	1219.982	1107.645	1019.272
**Qatar**	485.746	562.957	566.382	545.868	508.216	456.128
**Republic of Korea**	3869.064	4053.356	3860.883	3595.431	3288.747	3016.863
**Republic of Moldova**	116.257	119.84	111.935	100.998	87.107	70.982
**Romania**	887.61	940.987	865.18	789.281	713.683	654.005
**Russian Federation**	11589.736	11516.128	10904.882	10053.991	9038.071	8021.699
**Rwanda**	118.783	144.013	159.082	173.098	185.645	196.176
**Saudi Arabia**	3010.943	3446.449	3539.887	3457.639	3217.793	2892.002

**Senegal**	106.951	130.97	148.952	167.183	184.734	201.108
**Serbia**	327.927	344.118	327.51	304.456	275.122	245.93
**Sierra Leone**	19.348	22.022	23.493	25.11	26.652	28.038
**Singapore**	870.649	918.986	890.503	857.674	785.515	715.717
**Slovakia**	424.647	442.259	425.361	397.218	361.957	324.95
**Slovenia**	291	293.403	274.436	253.577	230.255	208.745
**South Africa**	2209.153	2369.012	2393.114	2382.805	2321.439	2207.66
**Spain**	8641.425	8747.979	8130.858	7412.082	6700.584	6186.27
**Sri Lanka**	269.066	283.942	276.32	266.567	251.6	236.473
**Sudan**	183.134	217.68	240.715	261.963	278.663	291.156
**Suriname**	19.788	20.78	20.209	19.512	18.767	17.928
**Sweden**	3144.829	3371	3344.387	3285.827	3203.6	3091.754
**Switzerland**	4730.65	4843.181	4579.141	4342.659	4090.35	3810.813
**Syrian Arab Republic**	135.736	162.275	157.544	149.217	142.381	139.696
**Tajikistan**	27.92	31.656	34.182	37.448	40.218	41.47
**Thailand**	1302.59	1338.951	1251.525	1159.703	1073.182	992.856
**Togo**	68.11	79.531	86.866	93.798	99.416	103.729
**Tunisia**	254.342	271.772	272.098	270.015	259.154	243.525
**Turkey**	3395.589	3638.789	3690.713	3647.855	3494.229	3311.512
**Uganda**	441.721	545.945	623.637	699.892	770.821	836.011
**Ukraine**	1016.82	1000.243	913.737	811.432	699.086	584.879
**United Kingdom**	18695.919	19307.607	18639.552	18044.634	17352.973	16523.358
**United Republic of Tanzania**	457.389	565.132	645.439	727.401	805.005	879.044

**United States of America**	149588.393	162360.201	161074.404	158152.688	153104.861	146339.643
**Uruguay**	233.568	250.963	252.741	248.782	237.935	225.605
**Uzbekistan**	349.626	375.468	379.992	387.157	383.486	365.472
**Venezuela (Bolivarian Republic of)**	720.374	746.563	707.427	660.047	598.246	535.601
**Viet Nam**	1501.632	1684.596	1708.842	1699.613	1643.8	1562.123
**Zambia**	167.168	205.979	235.558	263.206	285.637	305.233
**Zimbabwe**	118.565	131.592	137.774	142.948	145.703	146.888

D: Sensitivity analyses

Variation of all parameters

Tables S5 and **Table S6** show the total economic burden of depression in 2025–2050 for each country incorporating sensitivity analyses based on variations in all input parameters.

Table S5. Total macroeconomic cost, economic cost as a percentage of GDP in 2025–2050, and per capita cost attributable to depression, by country and World Bank region in 2024 U.S. $ with lower and upper bound, variation in all input parameters.

	Economic cost, 2024 U.S.$ million	Proportion of total GDP ($\times10^{-5}$), 2025–2050	Per capita cost, 2024 U.S.$
**East Asia and Pacific**			
**Australia**	225388(168089-315050)	560(437-712)	7528(5614-10522)
**Brunei Darussalam**	720(533-1019)	192(149-248)	1495(1106-2116)
**Cambodia**	3429(2416-5000)	363(264-484)	171(121-250)
**China**	1854105(1269365-2639361)	372(267-489)	1282(877-1824)
**Fiji**	380(278-538)	292(217-377)	377(275-534)
**Indonesia**	93698(68092-129932)	246(185-312)	300(218-416)
**Japan**	246547(195088-321139)	289(242-344)	2141(1694-2789)
**Lao People's Democratic Republic**	1565(1096-2280)	334(241-444)	179(125-261)

**Malaysia**	44965(32272-64115)	397(295-518)	1186(851-1691)
**Mongolia**	1973(1446-2792)	397(302-505)	494(362-699)
**Myanmar**	5779(3993-8702)	276(194-379)	96(66-145)
**New Zealand**	24606(18301-33470)	455(357-571)	4598(3420-6254)
**Philippines**	28669(21333-39348)	238(183-296)	217(162-298)
**Republic of Korea**	84509(65517-112632)	237(197-289)	1696(1315-2260)
**Singapore**	19679(15157-26601)	181(148-224)	3096(2384-4185)
**Thailand**	27793(19825-39900)	258(188-343)	403(287-578)
**Viet Nam**	38169(26989-55761)	320(231-428)	358(253-524)
**Europe and Central Asia**			
**Albania**	1355(984-1905)	313(235-404)	509(370-716)
**Armenia**	2282(1650-3230)	418(310-541)	783(566-1108)
**Austria**	39241(30005-53467)	410(327-505)	4275(3269-5824)
**Azerbaijan**	6815(4851-9796)	366(266-479)	624(444-897)
**Belarus**	8787(6398-12376)	531(394-682)	974(709-1372)
**Belgium**	66619(50633-91197)	530(422-659)	5531(4204-7571)
**Bosnia and Herzegovina**	1878(1359-2649)	353(264-460)	633(458-893)
**Bulgaria**	7040(5122-9896)	351(261-456)	1170(851-1644)
**Croatia**	6972(5060-9877)	451(332-589)	1891(1372-2678)
**Cyprus**	2827(2105-3978)	335(261-424)	2161(1609-3040)
**Czechia**	23336(17117-32691)	367(276-471)	2189(1606-3067)
**Denmark**	44801(33451-61577)	518(399-654)	7344(5484-10094)
**Estonia**	4101(2909-5891)	518(370-691)	3323(2358-4774)
**Finland**	34675(26714-46792)	569(464-699)	6247(4813-8429)
**France**	339097(256185-467345)	577(449-728)	5046(3812-6955)
**Georgia**	2982(2068-4314)	461(322-619)	800(555-1157)
**Germany**	409930(312173-563835)	496(394-615)	4990(3800-6864)
**Greece**	23361(17009-33371)	547(408-718)	2433(1771-3475)
**Hungary**	15722(11209-22589)	408(296-541)	1745(1244-2507)
**Iceland**	2793(2098-3891)	480(373-607)	7602(5710-10590)
**Ireland**	56459(43519-75479)	465(381-565)	10417(8030-13926)
**Italy**	176232(136229-237269)	437(351-534)	3066(2370-4128)
**Kazakhstan**	19084(14173-26529)	318(246-401)	870(646-1210)
**Kyrgyzstan**	1301(933-1871)	407(298-534)	161(115-231)
**Latvia**	4366(3200-6118)	529(393-682)	2689(1971-3768)

**Lithuania**	6285(4556-8934)	468(344-615)	2685(1946-3816)
**Luxembourg**	7181(5505-9774)	408(330-499)	9846(7549-13402)
**Montenegro**	522(373-758)	368(267-493)	852(609-1239)
**Netherlands**	99823(74888-138077)	492(384-621)	5738(4304-7936)
**North Macedonia**	1000(733-1402)	331(250-427)	505(370-708)
**Norway**	62886(48421-85043)	468(383-578)	10209(7861-13806)
**Poland**	48564(37360-65369)	315(248-384)	1363(1049-1835)
**Portugal**	29435(21846-41105)	581(439-744)	3058(2270-4270)
**Republic of Moldova**	2402(1745-3382)	498(367-649)	650(472-916)
**Romania**	18880(13917-26446)	283(213-362)	1076(793-1507)
**Russian Federation**	236918(182235-317655)	484(385-594)	1689(1299-2265)
**Serbia**	7139(5182-10065)	392(291-507)	913(663-1288)
**Slovakia**	9285(6692-13240)	369(270-487)	1768(1274-2521)
**Slovenia**	6061(4396-8592)	482(354-629)	3008(2182-4264)
**Spain**	178301(140182-232278)	619(512-732)	3929(3089-5119)
**Sweden**	74339(57862-98564)	577(479-690)	6814(5304-9034)
**Switzerland**	101078(77832-134986)	585(470-710)	10709(8246-14302)
**Tajikistan**	811(570-1187)	243(174-319)	61(43-89)
**Turkey**	80828(59552-114134)	354(269-458)	873(643-1233)
**Ukraine**	19748(14589-27243)	500(377-635)	510(377-703)
**United Kingdom**	413009(318133-558184)	593(478-727)	5743(4424-7762)
**Uzbekistan**	8694(6247-12394)	392(288-505)	219(158-312)
**Latin America and Caribbean**			
**Argentina**	37347(28870-49906)	249(206-303)	727(562-972)
**Bahamas**	1177(832-1685)	387(280-508)	2659(1881-3808)
**Barbados**	670(475-951)	503(362-662)	2343(1662-3324)
**Belize**	339(243-487)	407(298-530)	667(478-957)
**Bolivia (Plurinational State of)**	4855(3391-7124)	421(304-566)	340(238-500)
**Brazil**	198644(152984-265622)	442(353-528)	876(675-1171)
**Chile**	24257(17980-33906)	361(286-463)	1218(903-1703)
**Colombia**	23823(17474-33267)	296(226-379)	436(320-609)
**Costa Rica**	7716(5510-11005)	440(323-580)	1376(982-1962)
**Dominican Republic**	15986(11150-23398)	519(369-695)	1310(914-1917)
**Ecuador**	9736(6937-13971)	325(241-425)	459(327-659)
**El Salvador**	3407(2463-4810)	420(314-547)	496(359-701)

**Guatemala**	8645(6009-12770)	299(214-404)	369(256-545)
**Haiti**	2061(1448-2969)	420(303-557)	152(107-219)
**Honduras**	3476(2381-5229)	390(270-537)	280(192-422)
**Jamaica**	1602(1137-2286)	389(282-514)	529(375-755)
**Mexico**	116258(88856-157167)	310(246-377)	791(605-1070)
**Nicaragua**	1652(1178-2371)	402(295-527)	210(150-302)
**Panama**	6244(4532-8904)	280(213-360)	1179(856-1682)
**Paraguay**	4655(3399-6586)	384(288-494)	553(404-783)
**Peru**	9332(6789-13169)	158(120-201)	247(180-348)
**Suriname**	451(331-635)	559(421-721)	691(506-973)
**Uruguay**	5556(4203-7698)	325(259-409)	1542(1167-2136)
**Venezuela (Bolivarian Republic of)**	15486(11262-21979)	397(295-519)	442(321-627)
**Middle East and North Africa**			
**Algeria**	25362(18349-36554)	487(359-636)	467(338-673)
**Bahrain**	3881(2899-5416)	298(230-376)	1816(1357-2534)
**Djibouti**	537(377-786)	527(383-710)	452(317-662)
**Egypt**	42202(30933-59817)	297(226-378)	311(228-441)
**Iran (Islamic Republic of)**	51780(38140-72752)	603(456-778)	535(394-751)
**Iraq**	18964(14134-26378)	258(201-325)	327(244-455)
**Israel**	70305(52370-98664)	525(407-663)	6390(4760-8968)
**Jordan**	4186(3100-5913)	366(280-470)	362(268-511)
**Kuwait**	9552(7272-12994)	251(200-314)	1901(1448-2587)
**Lebanon**	2985(2155-4286)	513(376-680)	470(340-675)
**Malta**	1455(1088-2035)	398(312-501)	3298(2467-4613)
**Morocco**	18687(13557-26655)	505(378-656)	434(315-619)
**Oman**	17453(12734-25096)	524(393-682)	2771(2022-3985)
**Qatar**	12071(9119-16585)	194(154-243)	3413(2578-4689)
**Saudi Arabia**	75849(57100-104030)	287(224-361)	1828(1376-2508)
**Syrian Arab Republic**	3415(2458-4896)	463(340-607)	118(85-168)
**Tunisia**	6040(4396-8638)	534(398-695)	459(334-656)
**North America**			
**Canada**	218257(161355-300639)	450(348-567)	5108(3776-7035)
**United States of America**	3565500(2749756-4699126)	600(494-716)	9860(7604-12995)
**South Asia**			

**Bangladesh**	72356(50078-106589)	560(393-752)	391(271-576)
**Bhutan**	416(281-624)	514(354-707)	479(323-717)
**India**	391615(287770-542488)	365(278-458)	251(184-347)
**Maldives**	694(488-1008)	349(257-469)	1267(891-1840)
**Nepal**	7135(4740-10673)	607(418-855)	208(138-311)
**Pakistan**	51150(34960-75732)	451(315-611)	175(120-260)
**Sri Lanka**	6140(4462-8659)	366(273-471)	278(202-393)
**Sub-Saharan Africa**			
**Angola**	31588(22016-46604)	724(521-960)	558(389-824)
**Benin**	3231(2197-4870)	523(369-715)	172(117-259)
**Botswana**	1700(1233-2426)	293(222-374)	556(403-793)
**Burkina Faso**	3451(2368-5135)	509(364-687)	104(71-154)
**Burundi**	846(579-1260)	566(406-769)	44(30-65)
**Côte d'Ivoire**	9434(6612-13844)	375(276-501)	235(165-345)
**Cabo Verde**	413(293-591)	629(461-822)	646(459-925)
**Cameroon**	7212(4978-10717)	463(332-627)	180(125-268)
**Central African Republic**	169(122-242)	248(189-317)	25(18-35)
**Chad**	2674(1802-4068)	578(406-801)	102(69-155)
**Comoros**	199(139-289)	485(353-648)	163(115-238)
**Congo**	3474(2492-5031)	719(528-944)	415(298-601)
**Democratic Republic of the Congo**	16273(11555-23744)	694(507-920)	110(78-161)
**Eswatini**	847(596-1240)	574(420-762)	582(409-852)
**Ethiopia**	25484(17948-37006)	538(393-721)	152(107-221)
**Gabon**	2309(1689-3273)	361(273-463)	735(538-1043)
**Gambia**	440(291-678)	574(395-818)	116(77-178)
**Ghana**	10552(7260-15757)	516(365-702)	245(168-365)
**Guinea**	3843(2642-5715)	501(357-679)	189(130-281)
**Guinea-Bissau**	294(202-439)	511(364-695)	102(70-153)
**Kenya**	17852(13043-25233)	423(322-547)	235(171-332)
**Lesotho**	497(351-721)	771(557-1014)	202(143-293)
**Madagascar**	2679(1806-4055)	525(364-747)	63(43-96)
**Mali**	2873(1958-4278)	382(270-516)	87(59-129)
**Mauritania**	1267(885-1855)	331(241-444)	179(125-262)
**Mauritius**	1079(788-1507)	349(263-448)	866(632-1209)

**Mozambique**	3446(2317-5216)	427(299-594)	69(46-104)
**Namibia**	1626(1169-2340)	420(314-544)	482(346-693)
**Niger**	2313(1577-3458)	360(256-486)	50(34-75)
**Nigeria**	62940(45285-89851)	390(293-506)	201(144-286)
**Rwanda**	3805(2578-5732)	746(525-1038)	202(137-305)
**Senegal**	3606(2510-5300)	328(238-439)	140(97-205)
**Sierra Leone**	560(391-819)	462(336-619)	51(36-75)
**South Africa**	53675(41210-72140)	541(434-653)	769(591-1034)
**Sudan**	5537(3957-8052)	541(399-713)	85(61-124)
**Togo**	2056(1412-3066)	741(529-1008)	168(115-250)
**Uganda**	15169(10271-23097)	761(537-1057)	214(145-327)
**United Republic of Tanzania**	15638(10655-23609)	523(365-720)	161(109-242)
**Zambia**	5631(3916-8306)	518(376-696)	189(132-279)
**Zimbabwe**	3207(2173-4796)	360(250-499)	160(108-239)

Table S6. Total economic cost, cost as a percentage of GDP in 2025–2050, and per capita cost attributable to depression morbidity, by World Bank region and World Bank income group, and globally in billions of 2024 U.S.$ with an upper bound and lower bound, variation in all input parameters.

	Economic costs, 2024 U.S.$ billion	Proportion of total GDP ($\times 10^{-5}$), 2025–2050	Per capita cost, 2024 U.S.$
**By region**			
**East Asia and Pacific**	2702(1910-3798)	353(250-497)	1123(794-1578)
**Europe and Central Asia**	2715(2070-3697)	502(382-683)	2939(2241-4001)
**Latin America and Caribbean**	503(380-688)	353(266-482)	707(533-966)
**Middle East and North Africa**	365(270-511)	374(277-524)	703(521-986)
**North America**	3784(2911-5000)	589(453-778)	9358(7199-12365)
**South Asia**	530(383-746)	393(284-553)	252(183-356)
**Sub-Saharan Africa**	330(235-476)	496(354-716)	201(143-290)
**By income group**			
**Low income**	95(66-141)	570(396-843)	118(82-175)
**Lower middle income**	1071(772-1518)	378(273-536)	266(192-377)
**Upper middle income**	2853(2022-4006)	374(265-525)	1083(768-1521)
**High income**	6894(5287-9228)	521(399-697)	5721(4388-7658)
**Total**	10928(8159-14916)	457(341-624)	1256(938-1714)

Discounted estimates

Tables S7 and **Table S8** show the total economic burden of depression in 2025–2050 for each country using discount rates of 0% and 3%. **Table S9** and **Table S10** show the aggerated results by World Bank region, and by World Bank income group using discount rates of 0% and 3%.

Table S7. Total macroeconomic cost, economic cost as a percentage of GDP in 2025–2050, and per capita cost attributable to depression, by country and World Bank region in 2024 U.S. $ with lower and upper bound, discounting at 0%.

	Economic cost, 2024 U.S.$ million	Proportion of total GDP ($\times 10^{-5}$), 2025–2050	Per capita cost, 2024 U.S.$
**East Asia and Pacific**			
**Australia**	337807(271328-420009)	572(459-711)	11282(9062-14028)
**Brunei Darussalam**	1040(800-1342)	194(149-250)	2160(1661-2786)
**Cambodia**	5077(3980-6445)	363(284-461)	254(199-322)
**China**	2691486(2277605-3168020)	369(313-435)	1860(1574-2190)
**Fiji**	558(438-708)	292(229-370)	554(434-702)
**Indonesia**	138121(114123-166285)	247(204-297)	442(365-532)
**Japan**	355063(303565-413911)	293(251-342)	3084(2636-3595)
**Lao People's Democratic Republic**	2328(1793-2997)	334(257-430)	266(205-343)
**Malaysia**	66298(52443-83347)	399(315-501)	1748(1383-2198)
**Mongolia**	2939(2346-3671)	399(318-498)	736(587-919)
**Myanmar**	8584(6575-11132)	276(212-358)	143(109-185)
**New Zealand**	36432(30210-43850)	464(385-559)	6808(5645-8194)
**Philippines**	42526(35569-50713)	239(200-284)	322(269-384)
**Republic of Korea**	122327(101656-146929)	241(200-289)	2455(2040-2948)
**Singapore**	28562(23491-34665)	183(150-222)	4493(3695-5453)
**Thailand**	40023(31785-50228)	259(206-326)	580(461-728)
**Viet Nam**	56179(43611-71930)	321(249-411)	527(409-675)
**Europe and Central Asia**			
**Albania**	1953(1526-2484)	315(246-401)	734(573-933)
**Armenia**	3306(2625-4151)	418(332-525)	1134(900-1424)
**Austria**	57585(46672-70886)	416(337-512)	6273(5084-7722)
**Azerbaijan**	9932(7754-12651)	368(287-468)	909(710-1158)
**Belarus**	12768(10133-16012)	535(424-671)	1416(1123-1775)
**Belgium**	98689(79467-122106)	539(434-666)	8193(6597-10137)
**Bosnia and Herzegovina**	2706(2118-3440)	357(279-454)	912(714-1159)
**Bulgaria**	10079(8105-12501)	354(285-439)	1675(1347-2077)
**Croatia**	9952(7867-12515)	454(359-571)	2698(2133-3393)
**Cyprus**	4191(3299-5314)	339(267-430)	3203(2522-4062)
**Czechia**	33928(27247-42132)	372(299-462)	3183(2556-3953)
**Denmark**	66149(52509-83064)	525(417-660)	10844(8608-13616)
**Estonia**	5942(4718-7452)	521(414-654)	4815(3824-6039)
**Finland**	50965(41759-61994)	577(473-702)	9181(7523-11168)
**France**	502276(400472-627645)	588(469-735)	7475(5960-9340)
**Georgia**	4321(3408-5447)	463(365-583)	1159(914-1461)
**Germany**	602344(485013-746341)	503(405-623)	7333(5904-9086)
**Greece**	33473(25305-44058)	552(417-726)	3486(2635-4588)
**Hungary**	22750(18019-28576)	412(326-517)	2525(2000-3171)
**Iceland**	4120(3245-5200)	486(383-613)	11214(8831-14154)

**Ireland**	83217(68820-100493)	471(389-569)	15354(12698-18542)
**Italy**	254720(207878-310862)	442(361-539)	4431(3617-5408)
**Kazakhstan**	28244(22716-34981)	320(257-396)	1288(1036-1595)
**Kyrgyzstan**	1932(1532-2422)	410(325-514)	239(189-300)
**Latvia**	6262(5041-7741)	533(429-658)	3856(3104-4768)
**Lithuania**	9014(7176-11297)	472(376-592)	3851(3065-4826)
**Luxembourg**	10657(8677-13033)	413(337-506)	14614(11898-17871)
**Montenegro**	749(588-950)	369(289-467)	1224(961-1551)
**Netherlands**	147250(117064-184825)	501(398-629)	8463(6729-10623)
**North Macedonia**	1442(1132-1829)	335(263-425)	728(571-923)
**Norway**	92971(76338-112969)	476(391-578)	15093(12393-18340)
**Poland**	70516(58603-84448)	318(264-381)	1979(1645-2371)
**Portugal**	42689(33267-54524)	587(458-750)	4435(3456-5664)
**Republic of Moldova**	3419(2708-4295)	503(398-632)	926(733-1163)
**Romania**	27269(21764-34060)	286(229-358)	1554(1240-1941)
**Russian Federation**	342575(281131-415284)	487(400-590)	2442(2004-2961)
**Serbia**	10302(8202-12881)	396(315-495)	1318(1049-1648)
**Slovakia**	13419(10620-16883)	374(296-470)	2555(2022-3215)
**Slovenia**	8703(6927-10876)	483(384-604)	4319(3437-5397)
**Spain**	256431(218098-300588)	623(530-731)	5651(4806-6624)
**Sweden**	110594(92799-131488)	586(491-696)	10137(8506-12052)
**Switzerland**	148727(122943-179376)	594(491-716)	15757(13026-19004)
**Tajikistan**	1231(967-1559)	247(194-312)	93(73-117)
**Turkey**	120730(93944-154544)	362(281-463)	1304(1015-1669)
**Ukraine**	27997(22522-34577)	501(403-619)	723(581-893)
**United Kingdom**	613688(501823-747798)	602(493-734)	8534(6979-10399)
**Uzbekistan**	12821(10115-16166)	393(310-495)	323(255-408)
**Latin America and Caribbean**			
**Argentina**	55802(46428-66912)	254(211-305)	1086(904-1303)
**Bahamas**	1709(1322-2198)	390(302-501)	3861(2988-4968)
**Barbados**	962(741-1240)	505(389-651)	3364(2590-4336)
**Belize**	507(396-646)	410(320-522)	996(777-1269)
**Bolivia (Plurinational State of)**	7153(5603-9094)	421(330-535)	502(393-638)
**Brazil**	290350(246493-340859)	444(377-522)	1281(1087-1503)
**Chile**	36041(28677-45288)	369(294-464)	1810(1440-2275)
**Colombia**	34677(27932-42832)	297(240-367)	635(511-784)
**Costa Rica**	11286(8769-14460)	443(344-568)	2012(1563-2578)
**Dominican Republic**	23443(17886-30522)	518(395-675)	1921(1465-2501)
**Ecuador**	14414(11373-18235)	326(258-413)	680(536-860)
**El Salvador**	4975(3878-6355)	422(329-540)	725(565-926)
**Guatemala**	12896(10059-16519)	300(234-385)	550(429-705)
**Haiti**	3032(2316-3940)	420(321-546)	223(171-290)
**Honduras**	5169(3972-6696)	390(299-505)	417(320-540)
**Jamaica**	2312(1778-2990)	391(301-505)	763(587-987)
**Mexico**	171787(143163-205598)	313(261-375)	1169(975-1400)
**Nicaragua**	2429(1893-3104)	404(315-517)	309(241-395)
**Panama**	9364(7511-11704)	285(229-356)	1769(1419-2211)

**Paraguay**	6949(5451-8832)	387(304-492)	826(648-1050)
**Peru**	13851(11147-17207)	160(129-199)	367(295-455)
**Suriname**	663(517-844)	563(440-717)	1015(792-1292)
**Uruguay**	8258(6616-10291)	332(266-413)	2292(1836-2856)
**Venezuela (Bolivarian Republic of)**	22346(17221-28836)	397(306-512)	638(492-823)
**Middle East and North Africa**			
**Algeria**	37942(28952-49455)	491(375-640)	699(533-911)
**Bahrain**	5763(4578-7255)	303(241-381)	2697(2142-3395)
**Djibouti**	784(604-1011)	525(404-676)	660(509-851)
**Egypt**	64118(50494-81273)	299(235-379)	473(373-600)
**Iran (Islamic Republic of)**	76718(59636-98172)	612(476-783)	792(616-1014)
**Iraq**	29234(23491-36455)	263(212-328)	504(405-629)
**Israel**	105721(83730-133309)	530(420-669)	9609(7610-12116)
**Jordan**	6280(4908-8010)	372(291-474)	543(424-692)
**Kuwait**	14161(11531-17483)	259(211-319)	2819(2296-3480)
**Lebanon**	4354(3268-5747)	516(387-681)	686(515-906)
**Malta**	2157(1710-2720)	404(320-509)	4890(3876-6167)
**Morocco**	27706(21528-35511)	506(393-649)	644(500-825)
**Oman**	26292(20200-34025)	534(410-691)	4175(3208-5403)
**Qatar**	17944(14448-22335)	198(160-247)	5073(4085-6314)
**Saudi Arabia**	112322(90411-140018)	293(236-366)	2708(2179-3375)
**Syrian Arab Republic**	5062(3844-6614)	466(354-609)	174(132-228)
**Tunisia**	8947(6876-11596)	540(415-700)	679(522-880)
**North America**			
**Canada**	325918(266141-397736)	460(376-561)	7627(6228-9308)
**United States of America**	5301590(4575641-6134258)	616(531-712)	14661(12653-16963)
**South Asia**			
**Bangladesh**	107731(83050-138743)	563(434-725)	582(449-750)
**Bhutan**	617(478-793)	513(397-659)	710(549-911)
**India**	577966(479846-692696)	364(302-437)	370(307-443)
**Maldives**	1008(797-1272)	349(275-440)	1840(1454-2321)
**Nepal**	10474(8222-13268)	605(475-766)	306(240-387)
**Pakistan**	76550(60344-96439)	448(353-564)	262(207-331)
**Sri Lanka**	8973(7072-11306)	368(290-464)	407(321-513)
**Sub-Saharan Africa**			
**Angola**	48615(36808-63732)	723(548-948)	859(651-1127)
**Benin**	4884(3749-6312)	520(399-672)	259(199-335)
**Botswana**	2572(2078-3186)	296(239-367)	840(679-1041)
**Burkina Faso**	5235(4053-6724)	508(393-653)	157(122-202)
**Burundi**	1271(980-1639)	561(432-723)	66(51-85)
**Côte d'Ivoire**	14247(11060-18221)	376(292-481)	355(275-454)
**Cabo Verde**	610(478-776)	633(495-805)	955(748-1214)
**Cameroon**	10878(8403-13992)	461(356-593)	272(210-350)
**Central African Republic**	261(210-327)	255(204-319)	38(30-48)
**Chad**	4036(3068-5274)	575(437-752)	154(117-201)
**Comoros**	294(228-377)	483(374-619)	242(188-310)
**Congo**	5289(4049-6862)	722(552-936)	632(484-820)

**Democratic Republic of the Congo**	24810(18794-32517)	695(527-911)	168(127-221)
**Eswatini**	1275(985-1641)	576(445-742)	875(676-1127)
**Ethiopia**	38267(30586-47567)	535(428-666)	228(182-284)
**Gabon**	3495(2731-4461)	363(284-464)	1113(870-1421)
**Gambia**	660(498-869)	569(429-749)	173(131-228)
**Ghana**	15844(12154-20509)	514(394-665)	367(282-475)
**Guinea**	5818(4458-7531)	499(382-645)	286(219-370)
**Guinea-Bissau**	442(342-568)	510(395-656)	154(119-198)
**Kenya**	26592(22000-31995)	420(348-506)	350(289-421)
**Lesotho**	741(574-951)	773(599-993)	301(234-387)
**Madagascar**	3963(3041-5128)	517(397-669)	93(72-121)
**Mali**	4388(3386-5651)	381(294-491)	133(102-171)
**Mauritania**	1919(1493-2453)	332(258-424)	271(211-346)
**Mauritius**	1568(1235-1983)	354(279-447)	1258(991-1591)
**Mozambique**	5293(4146-6749)	427(334-544)	106(83-135)
**Namibia**	2443(1924-3089)	422(333-534)	724(570-915)
**Niger**	3579(2772-4601)	363(281-467)	78(60-100)
**Nigeria**	94487(76325-116270)	384(310-473)	301(243-371)
**Rwanda**	5673(4340-7363)	737(564-957)	302(231-392)
**Senegal**	5470(4272-6975)	329(257-420)	212(165-270)
**Sierra Leone**	834(647-1068)	462(358-592)	76(59-98)
**South Africa**	79126(66229-94115)	545(456-648)	1134(949-1349)
**Sudan**	8542(6450-11215)	545(412-716)	132(99-173)
**Togo**	3075(2356-3979)	737(565-954)	251(192-324)
**Uganda**	22824(17449-29671)	754(576-980)	323(247-420)
**United Republic of Tanzania**	23767(18299-30656)	519(399-669)	244(188-315)
**Zambia**	8522(6637-10878)	515(402-658)	287(223-366)
**Zimbabwe**	4725(3608-6129)	356(272-461)	236(180-306)

Table S8. Total macroeconomic cost, economic cost as a percentage of GDP in 2025–2050, and per capita cost attributable to depression, by country and World Bank region in 2024 U.S. $ with lower and upper bound, discounting at 3%

	Economic cost, 2024 U.S.$ million	Proportion of total GDP ($\times10^{-5}$), 2025–2050	Per capita cost, 2024 U.S.$
**East Asia and Pacific**			
**Australia**	229068(183548-285347)	566(454-705)	7650(6130-9530)
**Brunei Darussalam**	725(557-935)	193(148-249)	1505(1157-1942)
**Cambodia**	3400(2660-4325)	360(282-458)	170(133-216)
**China**	1830095(1546784-2156372)	366(310-432)	1265(1069-1490)
**Fiji**	380(298-483)	291(228-369)	377(295-479)
**Indonesia**	93495(77170-112657)	245(202-295)	299(247-361)
**Japan**	248298(212015-289750)	290(248-339)	2156(1841-2516)
**Lao People's Democratic Republic**	1557(1199-2006)	332(256-428)	178(137-229)
**Malaysia**	44741(35337-56316)	395(312-497)	1180(932-1485)
**Mongolia**	1975(1573-2471)	396(315-495)	495(394-619)
**Myanmar**	5781(4421-7505)	274(210-356)	96(73-125)
**New Zealand**	24857(20574-29962)	460(381-555)	4645(3845-5599)

**Philippines**	28563(23841-34123)	236(197-282)	216(180-258)
**Republic of Korea**	84707(70210-101961)	237(197-286)	1700(1409-2046)
**Singapore**	19684(16154-23931)	181(149-220)	3097(2541-3765)
**Thailand**	27762(21993-34911)	257(203-323)	402(319-506)
**Viet Nam**	38150(29579-48893)	320(248-410)	358(278-459)
**Europe and Central Asia**			
**Albania**	1355(1057-1725)	313(244-398)	509(397-648)
**Armenia**	2274(1801-2860)	415(329-522)	780(618-981)
**Austria**	39761(32144-49045)	411(333-507)	4331(3502-5343)
**Azerbaijan**	6814(5313-8688)	365(285-466)	624(487-796)
**Belarus**	8811(6982-11062)	532(421-667)	977(774-1226)
**Belgium**	67827(54523-84033)	534(429-662)	5631(4526-6976)
**Bosnia and Herzegovina**	1874(1464-2387)	353(276-450)	632(493-804)
**Bulgaria**	7022(5634-8726)	350(281-435)	1167(936-1450)
**Croatia**	6968(5502-8770)	451(356-567)	1889(1492-2378)
**Cyprus**	2854(2240-3628)	335(263-426)	2181(1712-2773)
**Czechia**	23426(18772-29140)	368(295-458)	2198(1761-2734)
**Denmark**	45514(36061-57237)	521(413-656)	7461(5911-9383)
**Estonia**	4115(3263-5166)	518(410-650)	3335(2644-4187)
**Finland**	35091(28701-42748)	572(468-697)	6322(5170-7701)
**France**	345887(275264-432838)	583(464-729)	5147(4096-6441)
**Georgia**	2974(2342-3752)	457(360-577)	797(628-1006)
**Germany**	415580(333798-515954)	498(400-618)	5059(4064-6281)
**Greece**	23535(17765-31010)	548(413-722)	2451(1850-3229)
**Hungary**	15800(12500-19862)	409(324-514)	1754(1387-2204)
**Iceland**	2845(2238-3594)	484(381-611)	7744(6092-9783)
**Ireland**	57026(47095-68941)	467(386-565)	10522(8690-12720)
**Italy**	178346(145307-217941)	438(357-535)	3103(2528-3792)
**Kazakhstan**	19152(15376-23754)	318(255-394)	873(701-1083)
**Kyrgyzstan**	1300(1029-1631)	406(321-509)	161(127-202)
**Latvia**	4376(3517-5416)	529(425-655)	2695(2166-3335)
**Lithuania**	6292(4996-7902)	468(372-588)	2688(2134-3376)
**Luxembourg**	7297(5933-8932)	410(333-501)	10006(8136-12248)
**Montenegro**	519(407-659)	366(287-464)	848(665-1076)
**Netherlands**	101532(80521-127687)	495(393-623)	5836(4628-7339)
**North Macedonia**	1002(785-1273)	332(260-421)	506(396-642)
**Norway**	63752(52258-77573)	472(387-575)	10350(8484-12594)
**Poland**	48979(40663-58705)	316(262-379)	1375(1141-1648)
**Portugal**	29829(23204-38149)	583(454-746)	3099(2411-3963)
**Republic of Moldova**	2402(1899-3021)	498(394-626)	650(514-818)
**Romania**	18992(15124-23765)	284(226-355)	1082(862-1354)
**Russian Federation**	238549(195592-289375)	485(398-588)	1701(1394-2063)
**Serbia**	7149(5682-8951)	392(312-491)	915(727-1145)
**Slovakia**	9293(7342-11708)	370(292-466)	1769(1398-2229)
**Slovenia**	6056(4815-7573)	480(382-601)	3005(2389-3758)
**Spain**	178641(151742-209625)	618(525-725)	3937(3344-4620)
**Sweden**	75307(63072-89671)	580(486-691)	6902(5781-8219)

**Switzerland**	102648(84722-123953)	589(486-711)	10875(8976-13133)
**Tajikistan**	813(638-1032)	243(190-308)	61(48-78)
**Turkey**	82190(63831-105366)	358(278-459)	888(689-1138)
**Ukraine**	19743(15874-24394)	500(402-618)	510(410-630)
**United Kingdom**	420710(343392-513406)	597(488-729)	5851(4775-7140)
**Uzbekistan**	8679(6834-10959)	390(307-493)	219(172-276)
**Latin America and Caribbean**			
**Argentina**	37754(31329-45370)	251(208-301)	735(610-883)
**Bahamas**	1175(908-1514)	387(299-498)	2656(2052-3422)
**Barbados**	670(516-865)	503(387-649)	2344(1803-3022)
**Belize**	340(265-434)	407(317-519)	669(521-853)
**Bolivia (Plurinational State of)**	4801(3753-6114)	417(326-531)	337(263-429)
**Brazil**	198947(168602-233902)	439(372-516)	877(744-1032)
**Chile**	24499(19427-30869)	364(289-459)	1230(976-1550)
**Colombia**	23700(19059-29311)	295(237-365)	434(349-537)
**Costa Rica**	7707(5977-9888)	439(341-564)	1374(1066-1763)
**Dominican Republic**	15929(12139-20761)	515(392-671)	1305(995-1701)
**Ecuador**	9675(7610-12269)	322(253-408)	456(359-579)
**El Salvador**	3398(2642-4350)	419(326-536)	495(385-634)
**Guatemala**	8569(6661-11006)	296(230-381)	366(284-470)
**Haiti**	2055(1568-2672)	418(319-544)	151(115-197)
**Honduras**	3449(2645-4474)	385(295-499)	278(213-361)
**Jamaica**	1600(1228-2072)	388(298-503)	528(405-684)
**Mexico**	116609(96960-139825)	309(257-371)	794(660-952)
**Nicaragua**	1645(1280-2106)	401(312-513)	210(163-268)
**Panama**	6238(4984-7822)	280(223-351)	1178(941-1477)
**Paraguay**	4673(3656-5952)	383(299-488)	555(434-707)
**Peru**	9340(7489-11639)	158(126-196)	247(198-308)
**Suriname**	454(354-578)	559(436-713)	695(542-886)
**Uruguay**	5628(4496-7030)	328(262-409)	1562(1248-1951)
**Venezuela (Bolivarian Republic of)**	15502(11933-20022)	396(305-511)	443(341-572)
**Middle East and North Africa**			
**Algeria**	25578(19498-33360)	487(371-635)	471(359-614)
**Bahrain**	3931(3112-4963)	298(236-377)	1840(1456-2322)
**Djibouti**	531(409-685)	522(402-673)	447(344-576)
**Egypt**	42304(33212-53761)	295(232-375)	312(245-397)
**Iran (Islamic Republic of)**	52729(40914-67577)	608(472-779)	545(423-698)
**Iraq**	19255(15416-24085)	260(208-325)	332(266-415)
**Israel**	71154(56246-89855)	526(416-664)	6467(5112-8167)
**Jordan**	4249(3312-5431)	368(287-470)	367(286-469)
**Kuwait**	9705(7873-12018)	253(205-314)	1932(1567-2392)
**Lebanon**	3020(2266-3988)	516(387-681)	476(357-628)
**Malta**	1469(1161-1857)	399(315-504)	3329(2631-4209)
**Morocco**	18709(14501-24028)	503(390-646)	435(337-558)
**Oman**	17735(13605-22977)	527(405-683)	2816(2160-3649)

**Qatar**	12281(9852-15332)	195(157-244)	3472(2785-4335)
**Saudi Arabia**	76635(61436-95845)	288(231-360)	1847(1481-2310)
**Syrian Arab Republic**	3459(2624-4522)	465(353-608)	119(90-156)
**Tunisia**	6096(4672-7917)	535(410-695)	463(355-601)
**North America**			
**Canada**	222392(181257-271814)	457(372-558)	5204(4242-6361)
**United States of America**	3612542(3111207-4187610)	608(523-704)	9990(8604-11580)
**South Asia**			
**Bangladesh**	72590(55911-93553)	558(430-720)	392(302-505)
**Bhutan**	415(321-534)	510(393-655)	477(369-614)
**India**	389433(322914-467246)	362(300-434)	249(207-299)
**Maldives**	684(539-865)	345(272-436)	1249(984-1579)
**Nepal**	7027(5507-8913)	599(469-760)	205(161-260)
**Pakistan**	50842(40040-64105)	446(351-563)	174(137-220)
**Sri Lanka**	6157(4849-7763)	367(289-462)	279(220-352)
**Sub-Saharan Africa**			
**Angola**	31677(23961-41560)	722(546-947)	560(424-735)
**Benin**	3205(2458-4145)	519(398-671)	170(131-220)
**Botswana**	1700(1368-2113)	291(234-362)	555(447-690)
**Burkina Faso**	3402(2630-4376)	503(389-647)	102(79-131)
**Burundi**	830(639-1072)	557(429-720)	43(33-56)
**Côte d'Ivoire**	9345(7245-11965)	373(289-477)	233(180-298)
**Cabo Verde**	410(321-523)	627(490-798)	642(502-818)
**Cameroon**	7129(5499-9179)	458(353-590)	178(138-230)
**Central African Republic**	169(135-213)	247(197-312)	25(20-31)
**Chad**	2638(2003-3452)	572(434-748)	101(76-132)
**Comoros**	196(152-252)	480(372-616)	161(125-207)
**Congo**	3499(2677-4543)	719(550-934)	418(320-543)
**Democratic Republic of the Congo**	16346(12370-21442)	693(524-909)	111(84-145)
**Eswatini**	844(651-1088)	573(442-738)	580(447-747)
**Ethiopia**	25117(20055-31251)	532(425-662)	150(120-186)
**Gabon**	2326(1814-2974)	361(281-461)	741(578-947)
**Gambia**	433(326-570)	567(427-747)	114(86-150)
**Ghana**	10494(8041-13597)	512(392-663)	243(186-315)
**Guinea**	3802(2911-4927)	496(380-643)	187(143-242)
**Guinea-Bissau**	290(224-374)	506(391-652)	101(78-130)
**Kenya**	17581(14524-21179)	417(345-502)	231(191-278)
**Lesotho**	496(383-638)	767(593-986)	202(156-259)
**Madagascar**	2624(2010-3399)	516(395-668)	62(47-80)
**Mali**	2858(2203-3684)	380(293-489)	86(67-111)
**Mauritania**	1256(975-1607)	329(256-421)	177(138-227)
**Mauritius**	1079(848-1367)	349(274-442)	866(680-1097)
**Mozambique**	3403(2657-4350)	422(329-539)	68(53-87)
**Namibia**	1624(1276-2058)	418(329-530)	481(378-610)
**Niger**	2295(1775-2954)	358(277-460)	50(39-64)
**Nigeria**	62496(50466-76919)	386(312-475)	199(161-245)
**Rwanda**	3734(2854-4852)	734(561-953)	199(152-258)

**Senegal**	3566(2779-4554)	326(254-416)	138(108-176)
**Sierra Leone**	553(428-709)	458(354-588)	51(39-65)
**South Africa**	53739(44917-63994)	541(452-644)	770(644-917)
**Sudan**	5630(4247-7397)	543(410-714)	87(65-114)
**Togo**	2032(1556-2631)	734(563-951)	166(127-214)
**Uganda**	14879(11360-19364)	750(572-975)	210(161-274)
**United Republic of Tanzania**	15477(11903-19984)	517(397-667)	159(122-205)
**Zambia**	5567(4330-7114)	512(398-654)	187(146-239)
**Zimbabwe**	3164(2416-4106)	355(271-461)	158(121-205)

Table S9. Total economic cost, cost as a percentage of GDP in 2025–2050, and per capita cost attributable to depression morbidity, by World Bank region and World Bank income group, and globally in billions of 2024 U.S.$ with an upper bound and lower bound, discounting at 0%

	**Economic costs, 2024 U.S.$ billion**	**Proportion of total GDP (×10⁻⁵), 2025–2050**	**Per capita cost, 2024 U.S.$**
**By region**			
**East Asia and Pacific**	3935(3301-4676)	354(297-420)	1636(1372-1944)
**Europe and Central Asia**	3985(3235-4895)	509(413-625)	4313(3501-5298)
**Latin America and Caribbean**	740(611-895)	356(294-430)	1040(858-1257)
**Middle East and North Africa**	546(430-691)	379(299-480)	1051(829-1331)
**North America**	5628(4842-6532)	604(519-701)	13917(11974-16154)
**South Asia**	783(640-955)	393(321-478)	374(305-455)
**Sub-Saharan Africa**	496(393-624)	494(391-621)	303(240-380)
**By income group**			
**Low income**	144(111-185)	568(440-730)	179(138-230)
**Lower middle income**	1590(1279-1967)	379(305-469)	395(318-489)
**Upper middle income**	4156(3475-4954)	373(312-445)	1578(1320-1881)
**High income**	10201(8568-12133)	532(447-633)	8466(7111-10069)
**Total**	16113(13452-19268)	463(386-554)	1852(1546-2214)

Table S10. Total economic cost, cost as a percentage of GDP in 2025–2050, and per capita cost attributable to depression morbidity, by World Bank region and World Bank income group, and globally in billions of 2024 U.S.$ with an upper bound and lower bound, discounting at 3%

	**Economic costs, 2024 U.S.$ billion**	**Proportion of total GDP (×10⁻⁵), 2025–2050**	**Per capita cost, 2024 U.S.$**
**By region**			
**East Asia and Pacific**	2683(2248-3192)	350(293-416)	1115(934-1327)
**Europe and Central Asia**	2751(2229-3384)	504(409-620)	2978(2413-3662)
**Latin America and Caribbean**	504(415-611)	352(290-426)	708(584-858)

**Middle East and North Africa**	369(290-468)	375(295-476)	711(559-902)
**North America**	3835(3292-4459)	596(512-693)	9484(8143-11029)
**South Asia**	527(430-643)	390(318-476)	251(205-307)
**Sub-Saharan Africa**	328(259-412)	492(389-619)	200(158-251)
**By income group**			
**Low income**	94(73-122)	565(437-727)	117(91-151)
**Lower middle income**	1068(858-1323)	376(302-466)	265(213-329)
**Upper middle income**	2833(2366-3381)	370(309-442)	1076(898-1284)
**High income**	6986(5856-8325)	526(440-626)	5798(4859-6908)
**Total**	10997(9164-13169)	458(382-549)	1264(1053-1513)

Sensitivity analyses for GDP input data

Tables S11-S14 show the total economic burden of depression in 2025–2050 for each country incorporating sensitivity analyses based on variations in GDP data from GBD network.[21] For **Table S11** and **Table S12,** we used the upper bound of GDP data as input to our model. For **Table S13** and **Table S14**, we used the lower bound of GDP data as input to our model.

Table S11. Total macroeconomic cost, economic cost as a percentage of GDP in 2025–2050, and per capita cost attributable to depression, by country and World Bank region in 2024 U.S. $ with lower and upper bound, discounting at 2%, using the upper bound of GDP data.

	Economic cost, 2024 U.S.$ million	Proportion of total GDP ($\times 10^{-5}$), 2025 − 2050	Per capita cost, 2024 U.S.$
**East Asia and Pacific**			
**Australia**	295834(236921-368673)	565(453-704)	9880(7913-12313)
**Brunei Darussalam**	904(695-1166)	193(149-249)	1877(1443-2421)
**Cambodia**	4519(3537-5743)	360(282-458)	226(177-287)
**China**	2383990(2014575-2809342)	366(310-432)	1648(1392-1942)
**Fiji**	487(381-619)	290(227-368)	483(378-614)
**Indonesia**	119819(98959-144289)	245(203-296)	383(317-462)
**Japan**	304123(259685-354905)	291(248-339)	2641(2255-3082)
**Lao People's Democratic Republic**	2052(1581-2642)	333(257-429)	235(181-302)
**Malaysia**	57564(45501-72398)	396(313-498)	1518(1200-1909)
**Mongolia**	2558(2036-3201)	396(315-496)	641(510-802)

**Myanmar**	8340(6376-10832)	274(210-356)	139(106-180)
**New Zealand**	31281(25893-37703)	460(381-555)	5845(4839-7045)
**Philippines**	36697(30633-43834)	236(197-282)	278(232-332)
**Republic of Korea**	107741(89351-129625)	238(197-286)	2162(1793-2601)
**Singapore**	25350(20795-30831)	180(148-219)	3988(3271-4850)
**Thailand**	34972(27711-43970)	257(204-323)	507(402-637)
**Viet Nam**	49399(38283-63336)	320(248-410)	464(359-595)
**Europe and Central Asia**			
**Albania**	1735(1354-2208)	314(245-400)	652(509-830)
**Armenia**	3018(2390-3797)	414(328-521)	1035(820-1303)
**Austria**	49622(40135-61185)	412(333-508)	5406(4372-6665)
**Azerbaijan**	8832(6882-11268)	365(285-466)	809(630-1032)
**Belarus**	11125(8822-13958)	532(422-668)	1233(978-1547)
**Belgium**	86168(69310-106692)	535(430-662)	7154(5754-8858)
**Bosnia and Herzegovina**	2418(1889-3079)	354(276-451)	815(637-1038)
**Bulgaria**	8771(7035-10903)	351(281-436)	1458(1169-1812)
**Croatia**	8661(6840-10899)	452(357-569)	2348(1855-2955)
**Cyprus**	4027(3158-5121)	334(262-425)	3078(2414-3914)
**Czechia**	29184(23397-36289)	369(296-458)	2738(2195-3405)
**Denmark**	56790(44983-71436)	521(413-656)	9309(7374-11710)
**Estonia**	5198(4123-6525)	518(411-650)	4213(3342-5288)
**Finland**	44313(36252-53970)	573(469-698)	7983(6531-9723)
**France**	422548(336491-528494)	584(465-730)	6288(5007-7865)
**Georgia**	3783(2983-4769)	460(363-580)	1014(800-1279)
**Germany**	520064(417682-645796)	498(400-619)	6331(5085-7862)
**Greece**	29203(22057-38464)	549(415-723)	3041(2297-4005)
**Hungary**	19641(15533-24702)	409(324-515)	2180(1724-2742)

**Iceland**	3570(2808-4509)	484(381-611)	9716(7643-12274)
**Ireland**	70947(58600-85757)	467(386-565)	13090(10812-15823)
**Italy**	218490(178089-266920)	439(358-537)	3801(3098-4644)
**Kazakhstan**	24784(19891-30749)	317(254-393)	1130(907-1402)
**Kyrgyzstan**	1673(1324-2100)	407(322-511)	207(164-260)
**Latvia**	5526(4444-6838)	530(426-656)	3403(2737-4211)
**Lithuania**	7994(6349-10039)	468(372-588)	3415(2712-4288)
**Luxembourg**	9083(7385-11118)	410(334-502)	12454(10127-15245)
**Montenegro**	663(520-841)	367(288-465)	1083(850-1373)
**Netherlands**	126280(100234-158679)	496(394-624)	7258(5761-9120)
**North Macedonia**	1261(988-1601)	333(261-423)	636(499-808)
**Norway**	79649(65282-96921)	472(387-574)	12931(10598-15735)
**Poland**	63147(52443-75659)	316(262-378)	1773(1472-2124)
**Portugal**	36559(28455-46736)	584(455-747)	3798(2956-4855)
**Republic of Moldova**	3477(2748-4375)	497(393-626)	941(744-1184)
**Romania**	24372(19407-30499)	284(226-355)	1389(1106-1738)
**Russian Federation**	301661(247409-365855)	485(398-588)	2151(1764-2608)
**Serbia**	9079(7215-11368)	393(312-492)	1162(923-1454)
**Slovakia**	11638(9196-14659)	371(293-467)	2216(1751-2791)
**Slovenia**	7548(6000-9441)	481(382-601)	3746(2978-4685)
**Spain**	218222(185481-255928)	619(526-726)	4809(4088-5640)
**Sweden**	96302(80636-114701)	580(485-690)	8827(7391-10513)
**Switzerland**	131075(108105-158391)	589(486-712)	13887(11454-16781)
**Tajikistan**	1072(841-1359)	245(192-310)	81(63-102)

**Turkey**	104662(81333-134112)	359(279-460)	1130(878-1449)
**Ukraine**	25794(20745-31860)	500(402-618)	666(536-822)
**United Kingdom**	516074(421495-629455)	598(489-730)	7177(5861-8753)
**Uzbekistan**	11260(8875-14205)	391(308-493)	284(224-358)
**Latin America and Caribbean**			
**Argentina**	49967(41472-60036)	251(208-302)	973(807-1169)
**Bahamas**	1467(1133-1889)	387(299-499)	3314(2561-4269)
**Barbados**	827(637-1065)	504(388-649)	2890(2227-3723)
**Belize**	454(354-580)	407(317-519)	893(696-1139)
**Bolivia (Plurinational State of)**	6114(4781-7784)	418(327-532)	429(335-546)
**Brazil**	254439(215768-298975)	441(374-518)	1122(952-1319)
**Chile**	31251(24813-39330)	365(290-459)	1570(1246-1975)
**Colombia**	30036(24171-37123)	296(238-365)	550(443-680)
**Costa Rica**	9761(7572-12520)	440(342-565)	1740(1350-2232)
**Dominican Republic**	20691(15786-26937)	516(394-672)	1695(1293-2207)
**Ecuador**	12287(9671-15573)	323(254-409)	579(456-734)
**El Salvador**	4273(3325-5465)	420(327-537)	622(484-796)
**Guatemala**	10746(8359-13795)	297(231-381)	459(357-589)
**Haiti**	2584(1974-3357)	419(320-544)	190(145-247)
**Honduras**	4451(3416-5771)	387(297-502)	359(276-466)
**Jamaica**	1983(1523-2566)	389(299-503)	654(503-847)
**Mexico**	146303(121732-175322)	310(258-372)	996(829-1193)
**Nicaragua**	2133(1659-2728)	402(313-514)	272(211-347)
**Panama**	8072(6444-10131)	279(223-351)	1525(1217-1914)
**Paraguay**	6139(4804-7818)	383(300-488)	730(571-929)
**Peru**	11982(9612-14925)	158(127-197)	317(254-395)

**Suriname**	590(460-752)	560(437-714)	903(704-1151)
**Uruguay**	7114(5680-8889)	328(262-410)	1974(1576-2467)
**Venezuela (Bolivarian Republic of)**	21321(16391-27565)	394(303-509)	609(468-787)
**Middle East and North Africa**			
**Algeria**	31706(24169-41357)	488(372-637)	584(445-762)
**Bahrain**	5045(3995-6368)	299(237-377)	2361(1869-2980)
**Djibouti**	710(546-915)	522(402-673)	598(460-770)
**Egypt**	56068(44014-71243)	295(232-375)	414(325-526)
**Iran (Islamic Republic of)**	66949(51972-85766)	609(473-781)	691(537-886)
**Iraq**	26132(20892-32717)	258(206-323)	451(360-564)
**Israel**	93615(73912-118334)	524(414-662)	8509(6718-10755)
**Jordan**	5558(4332-7104)	368(287-471)	480(374-614)
**Kuwait**	12681(10267-15729)	251(203-312)	2524(2044-3131)
**Lebanon**	4217(3166-5566)	516(387-681)	665(499-877)
**Malta**	1903(1504-2407)	398(315-504)	4314(3409-5456)
**Morocco**	24232(18792-31106)	502(390-645)	563(437-723)
**Oman**	23043(17679-29851)	530(406-686)	3659(2807-4740)
**Qatar**	15658(12555-19554)	195(156-243)	4427(3549-5528)
**Saudi Arabia**	100417(80428-125669)	287(230-359)	2421(1939-3029)
**Syrian Arab Republic**	4787(3634-6255)	465(353-608)	165(125-215)
**Tunisia**	7729(5934-10023)	536(412-696)	587(450-761)
**North America**			
**Canada**	278015(226591-339821)	457(372-558)	6506(5303-7952)
**United States of America**	4449389(3835081-5153480)	609(524-705)	12304(10605-14251)

**South Asia**			
**Bangladesh**	97299(74970-125345)	559(431-720)	526(405-677)
**Bhutan**	540(417-692)	511(395-656)	620(480-796)
**India**	502121(416782-601864)	362(301-434)	321(267-385)
**Maldives**	959(756-1213)	344(271-435)	1751(1379-2213)
**Nepal**	9085(7123-11521)	601(471-762)	265(208-336)
**Pakistan**	67189(52939-84679)	447(352-563)	230(181-290)
**Sri Lanka**	7855(6187-9902)	367(289-463)	356(281-449)
**Sub-Saharan Africa**			
**Angola**	43148(32653-56585)	722(546-947)	763(577-1000)
**Benin**	4016(3081-5193)	519(398-672)	213(164-276)
**Botswana**	2178(1754-2705)	292(235-362)	712(573-884)
**Burkina Faso**	4384(3388-5639)	505(390-649)	132(102-169)
**Burundi**	1169(900-1509)	558(429-720)	61(47-78)
**Côte d'Ivoire**	12226(9482-15650)	374(290-479)	305(236-390)
**Cabo Verde**	521(408-663)	628(491-799)	816(638-1038)
**Cameroon**	8991(6938-11572)	459(354-590)	225(174-290)
**Central African Republic**	222(177-280)	248(198-313)	32(26-41)
**Chad**	4206(3191-5501)	570(432-745)	160(122-210)
**Comoros**	285(221-365)	480(372-615)	234(182-300)
**Congo**	4874(3726-6331)	718(549-932)	582(445-756)
**Democratic Republic of the Congo**	22226(16819-29151)	693(524-909)	151(114-198)
**Eswatini**	1099(848-1415)	573(442-738)	755(582-972)
**Ethiopia**	35371(28225-44029)	532(425-662)	211(168-262)
**Gabon**	3101(2417-3964)	359(280-459)	988(770-1263)
**Gambia**	569(429-750)	567(428-747)	149(113-197)

**Ghana**	15154(11610-19634)	512(392-663)	351(269-455)
**Guinea**	4932(3780-6382)	497(381-643)	243(186-314)
**Guinea-Bissau**	412(319-531)	506(391-651)	144(111-185)
**Kenya**	23337(19284-28107)	417(345-503)	307(253-369)
**Lesotho**	663(513-853)	769(594-989)	270(209-347)
**Madagascar**	3317(2543-4294)	516(396-668)	78(60-101)
**Mali**	4296(3307-5543)	379(291-488)	130(100-168)
**Mauritania**	1690(1312-2164)	329(255-421)	238(185-305)
**Mauritius**	1344(1058-1701)	351(276-444)	1079(849-1365)
**Mozambique**	4522(3533-5776)	422(330-539)	90(71-115)
**Namibia**	2118(1666-2682)	419(329-530)	627(494-794)
**Niger**	3134(2422-4036)	359(278-463)	68(53-88)
**Nigeria**	83539(67460-102811)	384(310-472)	266(215-328)
**Rwanda**	4907(3751-6373)	734(561-954)	261(200-339)
**Senegal**	4573(3565-5839)	326(254-416)	177(138-226)
**Sierra Leone**	733(568-940)	459(356-589)	67(52-86)
**South Africa**	67851(56734-80774)	542(453-645)	972(813-1157)
**Sudan**	8222(6205-10799)	544(410-714)	127(96-166)
**Togo**	2596(1989-3361)	735(563-952)	212(162-274)
**Uganda**	19801(15113-25775)	750(573-977)	280(214-364)
**United Republic of Tanzania**	20795(15999-26837)	516(397-666)	214(164-276)
**Zambia**	7371(5727-9429)	512(398-655)	248(193-317)
**Zimbabwe**	4079(3115-5291)	355(271-461)	204(156-264)

Table S12. Total economic cost, cost as a percentage of GDP in 2025–2050, and per capita cost attributable to depression morbidity, by World Bank region and World Bank income group, and globally in billions of 2024 U.S.$ with an upper bound and lower bound using the upper bound of GDP data.

	Economic costs, 2024 U.S.$ billion	Proportion of total GDP (×10^{-5}), 2025 − 2050	Per capita cost, 2024 U.S.$
**By region**			
**East Asia and Pacific**	3466(2903-4123)	351(294-417)	1440(1207-1714)
**Europe and Central Asia**	3427(2778-4214)	504(408-620)	3709(3007-4562)
**Latin America and Caribbean**	645(532-781)	353(291-427)	906(747-1097)
**Middle East and North Africa**	480(378-610)	373(293-473)	926(728-1175)
**North America**	4727(4062-5493)	597(513-694)	11691(10045-13586)
**South Asia**	685(559-835)	391(319-477)	327(267-398)
**Sub-Saharan Africa**	438(346-551)	492(389-620)	267(211-336)
**By income group**			
**Low income**	130(100-167)	564(436-725)	161(124-207)
**Lower middle income**	1391(1118-1722)	377(303-467)	346(278-428)
**Upper middle income**	3663(3059-4371)	370(309-442)	1391(1162-1659)
**High income**	8663(7263-10321)	525(440-626)	7189(6027-8565)
**Total**	13868(11557-16608)	457(381-547)	1594(1328-1908)

Table S13. Total macroeconomic cost, economic cost as a percentage of GDP in 2025–2050, and per capita cost attributable to depression, by country and World Bank region in 2024 U.S. $ with lower and upper bound, discounting at 2% using the lower bound of GDP data.

	Economic cost, 2024 U.S.$ million	Proportion of total GDP (×10^{-5}), 2025 − 2050	Per capita cost, 2024 U.S.$
**East Asia and Pacific**			
**Australia**	227566(182829-282841)	571(459-710)	7600(6106-9446)
**Brunei Darussalam**	727(559-937)	193(149-249)	1509(1160-1946)

**Cambodia**	3358(2631-4266)	362(283-459)	168(131-213)
**China**	1805432(1528061-2124648)	368(311-433)	1248(1056-1469)
**Fiji**	384(301-488)	292(229-370)	381(299-484)
**Indonesia**	94887(78351-114293)	245(202-295)	304(251-366)
**Japan**	254519(217338-297018)	291(249-340)	2210(1888-2580)
**Lao People's Democratic Republic**	1505(1157-1940)	332(256-429)	172(132-222)
**Malaysia**	44908(35525-56448)	396(314-498)	1184(937-1489)
**Mongolia**	1968(1568-2460)	398(317-497)	493(393-616)
**Myanmar**	5113(3917-6631)	275(211-357)	85(65-110)
**New Zealand**	25100(20777-30260)	463(383-558)	4690(3882-5654)
**Philippines**	28485(23816-33979)	238(199-284)	216(180-257)
**Republic of Korea**	85165(70698-102384)	239(198-287)	1709(1419-2054)
**Singapore**	19464(16002-23630)	183(150-222)	3062(2517-3717)
**Thailand**	27778(22026-34906)	257(204-323)	403(319-506)
**Viet Nam**	38316(29729-49080)	321(249-411)	360(279-461)
**Europe and Central Asia**			
**Albania**	1349(1053-1718)	313(244-398)	507(396-645)
**Armenia**	2156(1711-2709)	418(332-525)	740(587-929)
**Austria**	40523(32811-49919)	413(334-509)	4414(3574-5438)
**Azerbaijan**	6685(5219-8515)	367(286-467)	612(478-780)
**Belarus**	8768(6952-11003)	533(422-669)	972(771-1220)
**Belgium**	68223(54887-84468)	536(431-663)	5664(4557-7012)
**Bosnia and Herzegovina**	1834(1434-2334)	354(277-451)	618(483-787)
**Bulgaria**	7090(5693-8805)	352(283-437)	1178(946-1463)
**Croatia**	7014(5541-8825)	451(357-568)	1902(1502-2393)

**Cyprus**	2321(1830-2940)	340(268-431)	1774(1399-2247)
**Czechia**	23610(18922-29370)	369(296-460)	2215(1775-2756)
**Denmark**	45949(36434-57752)	523(415-657)	7532(5972-9467)
**Estonia**	4163(3303-5224)	519(412-651)	3374(2677-4234)
**Finland**	35388(28971-43076)	574(470-699)	6375(5219-7760)
**France**	356433(283854-445819)	584(465-731)	5304(4224-6634)
**Georgia**	2998(2364-3780)	458(361-577)	804(634-1014)
**Germany**	419307(336996-520408)	500(402-620)	5104(4102-6335)
**Greece**	23980(18089-31625)	548(413-723)	2497(1884-3293)
**Hungary**	16109(12748-20249)	410(324-515)	1788(1415-2247)
**Iceland**	2865(2254-3620)	485(382-613)	7799(6135-9853)
**Ireland**	58970(48731-71260)	469(388-567)	10880(8991-13148)
**Italy**	184917(150779-225817)	439(358-536)	3217(2623-3929)
**Kazakhstan**	19073(15329-23637)	319(257-396)	870(699-1078)
**Kyrgyzstan**	1302(1032-1634)	407(322-510)	161(128-202)
**Latvia**	4404(3542-5448)	530(426-656)	2712(2182-3356)
**Lithuania**	6200(4928-7781)	470(374-590)	2648(2105-3324)
**Luxembourg**	7483(6089-9154)	411(335-503)	10261(8350-12552)
**Montenegro**	520(407-659)	366(287-464)	849(665-1076)
**Netherlands**	102372(81290-128609)	498(395-625)	5884(4672-7392)
**North Macedonia**	1022(801-1298)	332(261-422)	516(404-655)
**Norway**	65917(54057-80190)	475(389-578)	10701(8776-13019)
**Poland**	47683(39626-57103)	317(264-380)	1338(1112-1603)
**Portugal**	30864(24027-39449)	584(455-747)	3206(2496-4098)
**Republic of Moldova**	1910(1514-2399)	503(399-632)	517(410-649)
**Romania**	18724(14918-23424)	285(227-357)	1067(850-1335)
**Russian Federation**	238248(195491-288822)	486(399-589)	1699(1394-2059)
**Serbia**	7072(5624-8852)	393(313-492)	905(720-1133)

**Slovakia**	9431(7453-11880)	371(294-468)	1796(1419-2262)
**Slovenia**	6036(4799-7549)	481(383-602)	2995(2382-3746)
**Spain**	185297(157487-217328)	620(527-727)	4083(3471-4789)
**Sweden**	75199(63037-89482)	583(489-694)	6893(5778-8202)
**Switzerland**	103626(85617-125029)	591(489-713)	10979(9071-13247)
**Tajikistan**	811(636-1029)	243(190-308)	61(48-77)
**Turkey**	82821(64400-106060)	359(279-459)	895(696-1146)
**Ukraine**	19110(15370-23603)	500(402-618)	493(397-609)
**United Kingdom**	438158(357968-534288)	599(490-731)	6093(4978-7430)
**Uzbekistan**	8721(6868-11013)	391(308-494)	220(173-278)
**Latin America and Caribbean**			
**Argentina**	37137(30869-44564)	252(210-303)	723(601-868)
**Bahamas**	1207(934-1554)	388(300-499)	2728(2110-3511)
**Barbados**	678(522-874)	503(388-649)	2369(1824-3054)
**Belize**	330(258-421)	409(319-521)	648(506-826)
**Bolivia (Plurinational State of)**	4874(3813-6203)	419(327-533)	342(267-435)
**Brazil**	200915(170392-236074)	441(374-518)	886(751-1041)
**Chile**	24648(19589-30997)	367(291-461)	1238(984-1557)
**Colombia**	23804(19156-29424)	295(238-365)	436(351-539)
**Costa Rica**	7856(6096-10074)	441(342-565)	1401(1087-1796)
**Dominican Republic**	15690(11959-20448)	516(393-672)	1286(980-1675)
**Ecuador**	9916(7814-12557)	324(255-410)	468(368-592)
**El Salvador**	3436(2674-4393)	420(327-537)	500(390-640)
**Guatemala**	8842(6890-11333)	298(233-382)	377(294-484)
**Haiti**	2082(1589-2706)	419(320-544)	153(117-199)
**Honduras**	3526(2708-4569)	386(296-500)	284(218-369)

**Jamaica**	1640(1259-2124)	389(298-503)	541(416-701)
**Mexico**	121184(100857-145205)	311(259-373)	825(687-988)
**Nicaragua**	1646(1282-2104)	402(313-514)	210(163-268)
**Panama**	6229(4989-7797)	283(226-354)	1177(942-1473)
**Paraguay**	4571(3582-5816)	386(302-491)	543(426-691)
**Peru**	9413(7567-11702)	159(128-198)	249(200-310)
**Suriname**	450(351-572)	561(438-714)	688(538-876)
**Uruguay**	5724(4579-7140)	330(264-411)	1589(1271-1982)
**Venezuela (Bolivarian Republic of)**	13497(10416-17398)	399(308-514)	385(297-497)
**Middle East and North Africa**			
**Algeria**	26762(20411-34895)	488(372-636)	493(376-643)
**Bahrain**	3872(3075-4875)	301(239-379)	1812(1439-2281)
**Djibouti**	502(387-646)	524(404-674)	423(326-544)
**Egypt**	41839(32930-53060)	298(234-378)	309(243-391)
**Iran (Islamic Republic of)**	53127(41208-68120)	609(472-781)	549(426-704)
**Iraq**	18454(14831-23012)	264(212-329)	318(256-397)
**Israel**	71185(56360-89788)	530(419-668)	6470(5123-8161)
**Jordan**	4252(3320-5425)	370(289-472)	367(287-469)
**Kuwait**	9174(7487-11303)	260(212-320)	1826(1490-2250)
**Lebanon**	2629(1973-3471)	516(387-681)	414(311-547)
**Malta**	1433(1136-1808)	403(319-508)	3249(2574-4100)
**Morocco**	18640(14456-23931)	505(391-648)	433(336-556)
**Oman**	17038(13092-22045)	530(407-686)	2705(2079-3501)
**Qatar**	12221(9837-15214)	198(159-246)	3455(2781-4301)
**Saudi Arabia**	74197(59728-92480)	292(235-365)	1789(1440-2229)

**Syrian Arab Republic**	3177(2412-4151)	465(353-608)	109(83-143)
**Tunisia**	6161(4733-7986)	537(412-696)	468(359-606)
**North America**			
**Canada**	228920(186720-279627)	459(374-560)	5357(4370-6544)
**United States of America**	3685819(3176437-4270579)	612(527-709)	10193(8784-11810)
**South Asia**			
**Bangladesh**	67998(52426-87568)	560(432-722)	367(283-473)
**Bhutan**	416(322-535)	510(394-655)	479(370-615)
**India**	391179(324448-469251)	363(301-435)	250(208-300)
**Maldives**	650(513-819)	348(275-439)	1186(937-1495)
**Nepal**	7119(5584-9024)	601(471-761)	208(163-263)
**Pakistan**	50231(39588-63296)	447(352-563)	172(136-217)
**Sri Lanka**	6205(4889-7820)	367(289-462)	281(222-355)
**Sub-Saharan Africa**			
**Angola**	30870(23370-40471)	723(548-948)	546(413-715)
**Benin**	3356(2576-4336)	520(399-671)	178(137-230)
**Botswana**	1723(1391-2135)	294(237-364)	563(454-697)
**Burkina Faso**	3449(2670-4431)	505(391-649)	104(80-133)
**Burundi**	807(621-1040)	560(431-722)	42(32-54)
**Côte d'Ivoire**	9452(7332-12096)	374(290-478)	235(183-301)
**Cabo Verde**	416(325-529)	629(492-800)	651(509-828)
**Cameroon**	7459(5761-9593)	459(355-590)	187(144-240)
**Central African Republic**	167(134-210)	251(201-316)	24(19-31)
**Chad**	2266(1724-2960)	576(438-752)	86(66-113)
**Comoros**	168(131-216)	482(374-618)	138(107-177)
**Congo**	3335(2555-4323)	722(553-936)	398(305-516)

**Democratic Republic of the Congo**	15664(11868-20525)	694(526-910)	106(80-139)
**Eswatini**	838(647-1079)	574(443-739)	575(444-741)
**Ethiopia**	23322(18645-28989)	535(427-665)	139(111-173)
**Gabon**	2256(1764-2876)	364(285-464)	719(562-916)
**Gambia**	436(329-575)	568(428-749)	115(86-151)
**Ghana**	8857(6791-11470)	514(394-666)	205(157-266)
**Guinea**	3868(2964-5008)	497(381-643)	190(146-246)
**Guinea-Bissau**	275(213-354)	510(394-655)	96(74-123)
**Kenya**	17379(14377-20910)	419(346-504)	228(189-275)
**Lesotho**	482(373-620)	770(595-989)	196(152-252)
**Madagascar**	2705(2073-3504)	516(396-669)	64(49-83)
**Mali**	2554(1973-3284)	382(295-491)	77(60-99)
**Mauritania**	1211(942-1548)	331(258-423)	171(133-218)
**Mauritius**	1094(860-1385)	350(275-443)	878(690-1111)
**Mozambique**	3390(2653-4326)	425(332-542)	68(53-86)
**Namibia**	1610(1266-2040)	420(330-533)	477(375-604)
**Niger**	2254(1746-2897)	360(279-463)	49(38-63)
**Nigeria**	61259(49517-75328)	387(313-476)	195(158-240)
**Rwanda**	3740(2858-4860)	735(562-955)	199(152-259)
**Senegal**	3681(2873-4695)	327(256-418)	143(111-182)
**Sierra Leone**	547(423-702)	459(355-589)	50(39-64)
**South Africa**	54451(45530-64821)	542(453-645)	780(652-929)
**Sudan**	5029(3795-6608)	544(411-715)	78(58-102)
**Togo**	2075(1590-2685)	735(563-951)	169(130-219)
**Uganda**	14752(11274-19184)	752(575-978)	209(159-271)
**United Republic of Tanzania**	15285(11772-19711)	518(399-669)	157(121-202)

**Zambia**	5514(4294-7039)	514(400-656)	185(144-237)
**Zimbabwe**	3190(2437-4137)	355(271-461)	159(122-207)

Table S14. Total economic cost, cost as a percentage of GDP in 2025–2050, and per capita cost attributable to depression morbidity, by World Bank region and World Bank income group, and globally in billions of 2024 U.S.$ with an upper bound and lower bound, using the lower bound of GDP data.

Group	Economic costs, 2024 U.S.$ billion	Proportion of total GDP ($\times10^{-5}$), 2025 – 2050	Per capita cost, 2024 U.S.$
**By region**			
**East Asia and Pacific**	2665(2235-3166)	351(294-417)	1108(929-1316)
**Europe and Central Asia**	2803(2273-3445)	507(411-623)	3034(2460-3729)
**Latin America and Caribbean**	509(420-616)	353(292-427)	715(590-865)
**Middle East and North Africa**	365(287-462)	380(300-482)	703(554-891)
**North America**	3915(3363-4550)	600(515-697)	9682(8317-11253)
**South Asia**	524(428-638)	390(318-475)	250(204-304)
**Sub-Saharan Africa**	321(254-403)	493(391-620)	196(155-246)
**By income group**			
**Low income**	90(70-116)	569(440-731)	112(87-144)
**Lower middle income**	1062(855-1315)	376(303-466)	264(213-327)
**Upper middle income**	2814(2352-3354)	372(311-443)	1068(893-1274)
**High income**	7121(5974-8479)	529(444-630)	5910(4957-7036)
**Total**	11101(9261-13281)	462(385-552)	1276(1064-1526)

Sensitivity analyses for treatment costs input data

Tables S15-S18 show the total economic burden of depression in 2025–2050 for each country incorporating sensitivity analyses based on the upper bound and lower bound of treatment costs data. The upper bound is set as ten times of baseline treatment cost and lower bound is set as half of baseline treatment cost.

Table S15. Total macroeconomic cost, economic cost as a percentage of GDP in 2025–2050, and per capita cost attributable to depression, by country and World Bank region in 2024 U.S. $ with lower and upper bound, discounting at 2%. The treatment costs input is ten times of baseline treatment cost data.

	**Economic cost, 2024 U.S.$ million**	**Proportion of total GDP ($\times10^{-5}$), 2025 − 2050**	**Per capita cost, 2024 U.S.$**
**East Asia and Pacific**			
**Australia**	499138(447483-562998)	1093(980-1232)	16670(14945-18803)
**Brunei Darussalam**	945(757-1181)	224(180-280)	1962(1571-2452)
**Cambodia**	5873(5030-6925)	548(469-646)	293(251-346)
**China**	3305826(2985720-3674326)	586(529-651)	2285(2064-2540)
**Fiji**	660(567-776)	447(384-525)	655(562-769)
**Indonesia**	143527(125090-165153)	332(289-382)	459(400-529)
**Japan**	478526(437913-524917)	500(458-549)	4156(3803-4559)
**Lao People's Democratic Republic**	1954(1547-2463)	367(291-463)	224(177-282)
**Malaysia**	71355(60673-84506)	557(474-660)	1882(1600-2229)
**Mongolia**	3758(3303-4320)	665(584-764)	941(827-1082)
**Myanmar**	8762(7219-10718)	367(302-449)	146(120-178)
**New Zealand**	51581(46735-57357)	847(768-942)	9639(8733-10718)
**Philippines**	60761(55415-67050)	443(404-489)	460(420-508)
**Republic of Korea**	195463(179190-214832)	489(448-538)	3922(3596-4311)
**Singapore**	45109(41140-49884)	369(337-408)	7096(6472-7848)
**Thailand**	54078(47588-62124)	446(392-512)	784(690-900)
**Viet Nam**	60783(51087-72930)	451(379-541)	571(480-685)
**Europe and Central Asia**			

**Albania**	1995(1660-2412)	411(342-496)	750(624-906)
**Armenia**	4393(3859-5055)	713(626-821)	1507(1324-1734)
**Austria**	84137(75570-94579)	776(697-872)	9166(8232-10303)
**Azerbaijan**	10860(9167-12973)	517(436-617)	994(839-1188)
**Belarus**	14488(12432-17018)	777(667-913)	1606(1378-1887)
**Belgium**	124978(109948-143290)	875(770-1003)	10376(9128-11896)
**Bosnia and Herzegovina**	3260(2799-3837)	548(470-645)	1099(943-1293)
**Bulgaria**	14359(12799-16274)	640(571-725)	2386(2127-2704)
**Croatia**	10651(9001-12681)	616(521-734)	2888(2441-3439)
**Cyprus**	5918(5225-6790)	617(545-708)	4523(3994-5190)
**Czechia**	47891(42647-54331)	671(597-761)	4493(4001-5097)
**Denmark**	84315(73644-97553)	859(750-994)	13821(12072-15991)
**Estonia**	7131(6170-8316)	799(691-932)	5779(5001-6740)
**Finland**	66046(58837-74681)	958(854-1083)	11898(10599-13454)
**France**	633084(553732-730691)	949(830-1095)	9421(8240-10874)
**Georgia**	4474(3765-5349)	613(515-732)	1200(1010-1435)
**Germany**	863615(771463-976717)	921(823-1042)	10513(9391-11890)
**Greece**	35540(29069-43935)	740(606-915)	3701(3027-4575)
**Hungary**	25723(22016-30286)	594(508-699)	2855(2444-3361)
**Iceland**	4177(3492-5021)	632(528-759)	11368(9506-13667)
**Ireland**	112581(101356-126051)	819(737-917)	20772(18701-23257)
**Italy**	309277(272219-353685)	680(598-777)	5381(4736-6153)
**Kazakhstan**	35295(31023-40500)	518(455-594)	1609(1415-1847)
**Kyrgyzstan**	1965(1657-2341)	542(457-646)	243(205-290)
**Latvia**	7202(6240-8369)	778(674-904)	4436(3843-5154)

**Lithuania**	12467(11007-14283)	829(732-950)	5325(4702-6101)
**Luxembourg**	12541(11002-14387)	625(548-717)	17197(15086-19728)
**Montenegro**	828(702-986)	520(441-619)	1353(1147-1610)
**Netherlands**	207887(184201-237377)	904(801-1032)	11949(10587-13644)
**North Macedonia**	1742(1498-2047)	515(443-605)	879(756-1033)
**Norway**	130446(117440-146086)	858(773-961)	21177(19066-23717)
**Poland**	81325(71985-92246)	468(414-530)	2283(2021-2589)
**Portugal**	48047(40603-57403)	839(709-1003)	4992(4218-5963)
**Republic of Moldova**	3839(3277-4531)	713(609-842)	1039(887-1227)
**Romania**	37727(33388-43081)	503(445-575)	2150(1903-2455)
**Russian Federation**	359164(311054-416051)	651(564-754)	2561(2218-2966)
**Serbia**	12225(10575-14253)	598(518-698)	1564(1353-1824)
**Slovakia**	16533(14339-19249)	587(509-683)	3148(2730-3665)
**Slovenia**	9618(8221-11327)	680(582-801)	4773(4080-5621)
**Spain**	318596(288409-353350)	984(891-1091)	7021(6356-7787)
**Sweden**	161623(147765-177895)	1104(1009-1215)	14814(13544-16306)
**Switzerland**	205987(185762-230023)	1052(949-1175)	21824(19681-24370)
**Tajikistan**	1382(1181-1632)	363(310-428)	104(89-123)
**Turkey**	140393(119648-166579)	542(462-643)	1516(1292-1799)
**Ukraine**	25164(20834-30370)	570(472-688)	650(538-784)
**United Kingdom**	755140(667644-860082)	952(842-1085)	10501(9285-11961)
**Uzbekistan**	13613(11524-16196)	541(458-644)	343(291-408)

**Latin America and Caribbean**			
**Argentina**	84898(77623-93523)	499(457-550)	1653(1511-1821)
**Bahamas**	1940(1638-2323)	567(479-679)	4384(3702-5249)
**Barbados**	845(672-1063)	566(450-712)	2955(2348-3717)
**Belize**	547(462-654)	576(486-689)	1075(907-1285)
**Bolivia (Plurinational State of)**	8400(7212-9889)	644(553-758)	589(506-694)
**Brazil**	363995(329736-403465)	714(647-792)	1605(1454-1779)
**Chile**	55177(49440-62383)	728(652-823)	2771(2483-3133)
**Colombia**	39090(33853-45421)	431(374-501)	716(620-832)
**Costa Rica**	12931(10973-15402)	654(555-779)	2305(1956-2746)
**Dominican Republic**	22131(17851-27582)	633(511-789)	1813(1463-2260)
**Ecuador**	20107(17771-23040)	591(523-678)	948(838-1086)
**El Salvador**	5879(5024-6954)	643(549-760)	856(732-1013)
**Guatemala**	18339(16171-21105)	558(492-643)	783(690-901)
**Haiti**	2709(2158-3408)	487(388-613)	199(159-251)
**Honduras**	5706(4790-6875)	561(471-676)	460(386-555)
**Jamaica**	2374(1957-2904)	513(423-627)	784(646-958)
**Mexico**	239436(217264-265623)	563(511-625)	1630(1479-1808)
**Nicaragua**	2843(2429-3363)	613(523-725)	362(309-428)
**Panama**	17382(15956-19183)	688(631-759)	3283(3014-3623)
**Paraguay**	9018(7862-10473)	653(569-758)	1072(934-1245)
**Peru**	22858(20763-25461)	342(310-381)	605(549-674)
**Suriname**	702(589-843)	768(644-922)	1075(902-1291)
**Uruguay**	12051(10776-13628)	623(557-705)	3345(2991-3782)
**Venezuela (Bolivarian Republic of)**	24153(20138-29237)	549(457-664)	689(575-835)

**Middle East and North Africa**			
**Algeria**	40459(33553-49300)	680(564-829)	745(618-908)
**Bahrain**	9184(8260-10346)	620(557-698)	4297(3865-4841)
**Djibouti**	737(599-911)	640(520-791)	621(504-767)
**Egypt**	87354(76987-100410)	535(472-615)	644(568-741)
**Iran (Islamic Republic of)**	77854(64538-94582)	798(661-969)	804(667-977)
**Iraq**	53508(49139-58997)	634(582-699)	923(847-1017)
**Israel**	149085(132126-170357)	972(862-1111)	13550(12009-15484)
**Jordan**	7851(6790-9187)	602(520-704)	679(587-794)
**Kuwait**	31679(29610-34292)	737(689-798)	6306(5894-6827)
**Lebanon**	3397(2549-4485)	516(387-681)	535(402-707)
**Malta**	3362(3014-3800)	810(726-915)	7621(6834-8613)
**Morocco**	33125(28350-39161)	786(673-929)	770(659-910)
**Oman**	26575(21894-32514)	699(576-855)	4220(3477-5163)
**Qatar**	33651(30921-37075)	476(438-525)	9514(8742-10482)
**Saudi Arabia**	223910(206736-245621)	749(691-821)	5397(4983-5921)
**Syrian Arab Republic**	4337(3395-5538)	516(404-659)	149(117-191)
**Tunisia**	10486(8878-12541)	816(691-976)	796(674-952)
**North America**			
**Canada**	427679(381137-483608)	778(694-880)	10008(8919-11317)
**United States of America**	8977826(8410050-9628859)	1340(1256-1438)	24827(23257-26627)
**South Asia**			
**Bangladesh**	98518(79600-122282)	669(541-830)	532(430-661)
**Bhutan**	549(441-683)	594(478-740)	631(507-785)

**India**	596548(521268-684539)	489(427-561)	382(334-438)
**Maldives**	1284(1120-1487)	573(500-664)	2343(2044-2715)
**Nepal**	10966(9239-13110)	824(695-986)	320(270-382)
**Pakistan**	67623(55315-82729)	521(426-637)	232(190-284)
**Sri Lanka**	8567(7091-10378)	453(375-548)	389(322-471)
**Sub-Saharan Africa**			
**Angola**	42066(33207-53405)	835(659-1060)	744(587-944)
**Benin**	4169(3316-5243)	590(469-741)	221(176-278)
**Botswana**	4507(4128-4978)	679(622-750)	1472(1349-1626)
**Burkina Faso**	5427(4540-6544)	700(586-845)	163(136-197)
**Burundi**	1270(1051-1547)	745(616-907)	66(55-80)
**Côte d'Ivoire**	13503(11105-16492)	471(388-576)	336(277-411)
**Cabo Verde**	674(572-801)	909(772-1080)	1055(896-1254)
**Cameroon**	10679(8815-13023)	600(495-731)	267(221-326)
**Central African Republic**	542(503-593)	696(645-761)	79(73-86)
**Chad**	3905(3175-4838)	739(601-915)	149(121-184)
**Comoros**	286(235-349)	615(506-751)	235(193-287)
**Congo**	5024(4084-6217)	905(735-1119)	600(488-743)
**Democratic Republic of the Congo**	22851(18305-28671)	847(679-1063)	155(124-194)
**Eswatini**	1317(1097-1595)	784(653-949)	905(753-1096)
**Ethiopia**	35349(29551-42369)	655(548-785)	211(176-253)
**Gabon**	4579(3995-5316)	624(545-725)	1458(1273-1693)
**Gambia**	590(468-748)	675(535-856)	155(123-196)
**Ghana**	14657(11849-18208)	627(507-778)	340(275-422)
**Guinea**	4890(3868-6179)	557(440-704)	240(190-304)
**Guinea-Bissau**	469(393-564)	716(600-862)	163(137-197)

**Kenya**	28828(25341-32931)	600(527-685)	379(333-433)
**Lesotho**	777(649-938)	1058(884-1278)	316(264-382)
**Madagascar**	3574(2875-4456)	615(495-767)	84(68-105)
**Mali**	4264(3513-5211)	494(407-603)	129(106-158)
**Mauritania**	2084(1763-2488)	478(404-570)	294(249-351)
**Mauritius**	1960(1699-2284)	565(490-658)	1572(1363-1833)
**Mozambique**	7371(6512-8459)	795(702-912)	147(130-169)
**Namibia**	3063(2667-3559)	694(604-806)	907(790-1054)
**Niger**	4145(3544-4907)	562(481-665)	90(77-107)
**Nigeria**	90903(77147-107396)	490(416-579)	290(246-342)
**Rwanda**	5206(4197-6484)	895(721-1115)	277(223-345)
**Senegal**	6514(5613-7646)	520(448-610)	252(217-296)
**Sierra Leone**	866(724-1045)	631(527-760)	79(66-96)
**South Africa**	87362(77401-98933)	778(689-881)	1252(1109-1418)
**Sudan**	7132(5553-9150)	602(469-773)	110(86-141)
**Togo**	2497(1954-3180)	790(619-1006)	203(159-259)
**Uganda**	22481(18439-27631)	988(810-1214)	318(261-391)
**United Republic of Tanzania**	22195(18095-27360)	646(526-796)	228(186-281)
**Zambia**	8855(7437-10627)	711(597-853)	298(250-357)
**Zimbabwe**	3601(2750-4672)	355(271-461)	180(137-233)

Table S16. Total economic cost, cost as a percentage of GDP in 2025–2050, and per capita cost attributable to depression morbidity, by World Bank region and World Bank income group, and globally in billions of 2024 U.S.$ with an upper bound and lower bound. The treatment costs input is ten times of baseline treatment cost data.

Group	Economic costs, 2024 U.S.$ billion	Proportion of total GDP ($\times 10^{-5}$), 2025 – 2050	Per capita cost, 2024 U.S.$
**By region**			
**East Asia and Pacific**	4988(4496-5562)	577(520-644)	2073(1869-2312)

**Europe and Central Asia**	5070(4482-5782)	827(731-944)	5488(4851-6259)
**Latin America and Caribbean**	974(873-1094)	602(540-677)	1367(1226-1536)
**Middle East and North Africa**	797(707-909)	716(636-818)	1535(1363-1752)
**North America**	9406(8791-10112)	1298(1213-1395)	23261(21742-25009)
**South Asia**	784(674-915)	512(440-597)	374(321-436)
**Sub-Saharan Africa**	490(412-587)	645(542-772)	299(251-358)
**By income group**			
**Low income**	137(113-168)	717(589-879)	170(140-209)
**Lower middle income**	1643(1405-1931)	510(436-600)	408(349-480)
**Upper middle income**	5098(4570-5716)	591(529-662)	1936(1735-2170)
**High income**	15606(14329-17118)	1044(958-1145)	12951(11891-14205)
**Total**	22508(20436-24962)	832(756-923)	2586(2348-2868)

Table S17. Total macroeconomic cost, economic cost as a percentage of GDP in 2025–2050, and per capita cost attributable to depression, by country and World Bank region in 2024 U.S. $ with lower and upper bound, discounting at 2%. The treatment costs input is half of baseline treatment cost data.

	Economic cost, 2024 U.S.$ million	Proportion of total GDP ($\times10^{-5}$), 2025 – 2050	Per capita cost, 2024 U.S.$
**East Asia and Pacific**			
**Australia**	246142(194779-309636)	539(426-678)	8221(6505-10341)
**Brunei Darussalam**	806(618-1042)	191(147-247)	1674(1284-2163)
**Cambodia**	3758(2920-4804)	351(272-448)	188(146-240)

**China**	2002323(1682646-2370435)	355(298-420)	1384(1163-1638)
**Fiji**	417(324-533)	282(219-361)	414(322-529)
**Indonesia**	103917(85437-125611)	240(198-291)	333(273-402)
**Japan**	267244(226711-313549)	279(237-328)	2321(1969-2723)
**Lao People's Democratic Republic**	1762(1354-2273)	331(254-427)	202(155-260)
**Malaysia**	49625(38998-62692)	387(304-489)	1309(1028-1653)
**Mongolia**	2160(1705-2721)	382(302-481)	541(427-681)
**Myanmar**	6441(4898-8399)	270(205-352)	107(81-139)
**New Zealand**	26803(21976-32555)	440(361-535)	5008(4107-6083)
**Philippines**	30897(25548-37194)	225(186-271)	234(193-282)
**Republic of Korea**	89723(73470-109074)	225(184-273)	1800(1474-2189)
**Singapore**	20908(16941-25684)	171(139-210)	3289(2665-4041)
**Thailand**	29943(23474-37957)	247(194-313)	434(340-550)
**Viet Nam**	42237(32542-54386)	313(241-403)	397(306-511)
**Europe and Central Asia**			
**Albania**	1497(1163-1912)	308(239-393)	562(437-718)
**Armenia**	2461(1929-3122)	399(313-507)	844(662-1071)
**Austria**	42570(34028-52981)	393(314-489)	4637(3707-5772)
**Azerbaijan**	7518(5827-9629)	358(277-458)	688(534-882)
**Belarus**	9664(7612-12189)	519(409-654)	1071(844-1351)
**Belgium**	73805(58837-92037)	517(412-644)	6127(4885-7641)
**Bosnia and Herzegovina**	2043(1582-2620)	343(266-440)	688(533-883)
**Bulgaria**	7520(5964-9430)	335(266-420)	1250(991-1567)
**Croatia**	7650(6009-9667)	443(348-559)	2074(1629-2621)
**Cyprus**	3078(2386-3948)	321(249-411)	2352(1824-3018)

**Czechia**	25186(19962-31599)	353(279-442)	2363(1873-2965)
**Denmark**	49465(38843-62637)	504(396-638)	8109(6367-10268)
**Estonia**	4489(3533-5669)	503(396-635)	3638(2863-4594)
**Finland**	38069(30908-46642)	552(448-677)	6858(5568-8403)
**France**	376229(296716-474188)	564(445-711)	5599(4416-7057)
**Georgia**	3289(2580-4164)	450(353-570)	882(692-1117)
**Germany**	446061(354309-558676)	476(378-596)	5430(4313-6801)
**Greece**	25827(19379-34184)	538(404-712)	2689(2018-3560)
**Hungary**	17310(13591-21895)	400(314-505)	1921(1508-2430)
**Iceland**	3149(2466-3990)	476(373-604)	8570(6713-10861)
**Ireland**	61701(50537-75090)	449(368-546)	11384(9324-13855)
**Italy**	193825(156732-238331)	426(344-524)	3372(2727-4146)
**Kazakhstan**	20952(16683-26154)	307(245-384)	955(761-1193)
**Kyrgyzstan**	1449(1142-1824)	399(315-503)	179(141-226)
**Latvia**	4778(3817-5944)	516(412-642)	2943(2351-3661)
**Lithuania**	6754(5304-8557)	449(353-569)	2885(2266-3655)
**Luxembourg**	7998(6460-9842)	399(322-491)	10967(8859-13495)
**Montenegro**	570(444-727)	358(279-457)	931(725-1187)
**Netherlands**	109152(85491-138627)	474(372-603)	6274(4914-7968)
**North Macedonia**	1091(847-1395)	322(250-412)	550(428-704)
**Norway**	68670(55739-84220)	452(367-554)	11148(9049-13673)
**Poland**	53584(44276-64462)	308(255-371)	1504(1243-1809)
**Portugal**	32630(25201-41968)	570(440-733)	3390(2618-4360)
**Republic of Moldova**	2625(2063-3317)	488(383-616)	711(559-898)
**Romania**	20402(16082-25727)	272(215-343)	1163(917-1466)
**Russian Federation**	262758(214626-319700)	476(389-580)	1873(1530-2279)
**Serbia**	7801(6155-9823)	382(301-481)	998(787-1257)
**Slovakia**	10114(7916-12837)	359(281-456)	1926(1507-2444)

**Slovenia**	6642(5250-8345)	470(371-590)	3296(2605-4141)
**Spain**	193891(163696-228682)	599(506-706)	4273(3607-5040)
**Sweden**	80921(67136-97104)	553(458-663)	7417(6154-8900)
**Switzerland**	110576(90430-134522)	565(462-687)	11715(9581-14252)
**Tajikistan**	904(704-1153)	237(185-302)	68(53-87)
**Turkey**	90355(69670-116454)	349(269-449)	976(752-1258)
**Ukraine**	21904(17576-27106)	496(398-614)	565(454-700)
**United Kingdom**	459280(372102-563847)	579(469-711)	6387(5175-7841)
**Uzbekistan**	9632(7548-12206)	383(300-485)	243(190-308)
**Latin America and Caribbean**			
**Argentina**	40467(33234-49036)	238(195-288)	788(647-955)
**Bahamas**	1291(990-1672)	378(290-489)	2918(2238-3778)
**Barbados**	747(574-966)	500(384-646)	2612(2005-3375)
**Belize**	378(293-485)	398(309-511)	743(576-953)
**Bolivia (Plurinational State of)**	5296(4109-6782)	406(315-520)	371(288-476)
**Brazil**	216814(182706-256099)	426(359-503)	956(806-1129)
**Chile**	26191(20476-33369)	346(270-440)	1315(1028-1676)
**Colombia**	26085(20871-32384)	288(230-357)	478(382-593)
**Costa Rica**	8474(6527-10926)	429(330-553)	1511(1164-1948)
**Dominican Republic**	17802(13525-23249)	509(387-665)	1459(1108-1905)
**Ecuador**	10491(8157-13423)	309(240-395)	495(385-633)
**El Salvador**	3727(2875-4799)	408(314-525)	543(419-699)
**Guatemala**	9302(7135-12068)	283(217-367)	397(305-515)
**Haiti**	2308(1757-3007)	415(316-541)	170(129-221)
**Honduras**	3831(2916-4998)	377(287-491)	309(235-403)
**Jamaica**	1767(1350-2297)	382(292-496)	583(445-758)

**Mexico**	126165(103983-152373)	297(245-358)	859(708-1037)
**Nicaragua**	1811(1397-2332)	390(301-502)	231(178-297)
**Panama**	6537(5116-8332)	259(202-330)	1235(966-1574)
**Paraguay**	5104(3952-6551)	369(286-474)	606(470-778)
**Peru**	9916(7832-12501)	148(117-187)	262(207-331)
**Suriname**	502(390-642)	549(426-702)	768(596-983)
**Uruguay**	6050(4771-7634)	313(247-395)	1679(1324-2119)
**Venezuela (Bolivarian Republic of)**	17061(13053-22137)	388(296-503)	487(373-632)
**Middle East and North Africa**			
**Algeria**	28392(21529-37164)	477(362-625)	523(396-684)
**Bahrain**	4184(3263-5344)	282(220-360)	1958(1527-2500)
**Djibouti**	594(456-768)	516(396-667)	500(384-647)
**Egypt**	46200(35862-59219)	283(220-363)	341(265-437)
**Iran (Islamic Republic of)**	58399(45126-75070)	598(462-769)	603(466-775)
**Iraq**	20293(15931-25774)	240(189-305)	350(275-444)
**Israel**	77013(60191-98094)	502(393-640)	7000(5471-8916)
**Jordan**	4651(3595-5982)	357(276-458)	402(311-517)
**Kuwait**	9806(7749-12405)	228(180-289)	1952(1543-2469)
**Lebanon**	3395(2548-4482)	516(387-681)	535(401-706)
**Malta**	1567(1220-2003)	377(294-483)	3552(2766-4541)
**Morocco**	20561(15803-26574)	488(375-631)	478(367-618)
**Oman**	19772(15089-25718)	520(397-677)	3140(2396-4084)
**Qatar**	12774(10046-16202)	181(142-229)	3612(2840-4581)
**Saudi Arabia**	79026(61970-100577)	264(207-336)	1905(1494-2424)
**Syrian Arab Republic**	3884(2945-5079)	462(350-604)	134(101-175)

**Tunisia**	6697(5091-8751)	521(396-681)	508(386-664)
**North America**			
**Canada**	241578(195204-297289)	440(355-541)	5653(4568-6957)
**United States of America**	3812126(3247649-4459665)	569(485-666)	10542(8981-12333)
**South Asia**			
**Bangladesh**	81538(62640-105280)	554(425-715)	441(338-569)
**Bhutan**	467(360-602)	506(390-652)	537(414-692)
**India**	433561(358264-521610)	356(294-428)	277(229-334)
**Maldives**	747(583-951)	334(261-425)	1363(1065-1735)
**Nepal**	7823(6101-9961)	588(459-749)	228(178-291)
**Pakistan**	57485(45209-72543)	443(348-559)	197(155-249)
**Sri Lanka**	6857(5385-8661)	362(284-458)	311(244-393)
**Sub-Saharan Africa**			
**Angola**	36080(27256-47367)	716(541-941)	638(482-837)
**Benin**	3645(2790-4721)	515(394-668)	194(148-251)
**Botswana**	1801(1424-2269)	271(215-342)	588(465-741)
**Burkina Faso**	3828(2941-4945)	494(380-638)	115(88-149)
**Burundi**	935(717-1211)	548(420-710)	49(37-63)
**Côte d'Ivoire**	10553(8156-13543)	368(285-473)	263(203-337)
**Cabo Verde**	455(353-582)	613(476-785)	712(552-911)
**Cameroon**	8035(6168-10383)	451(346-583)	201(154-260)
**Central African Republic**	175(136-226)	225(175-290)	25(20-33)
**Chad**	2979(2251-3910)	564(426-740)	114(86-149)
**Comoros**	220(170-283)	473(365-608)	181(140-233)
**Congo**	3942(3004-5133)	710(541-924)	471(359-613)

**Democratic Republic of the Congo**	18481(13949-24281)	685(517-900)	125(95-165)
**Eswatini**	944(724-1222)	562(431-727)	649(497-839)
**Ethiopia**	28408(22623-35415)	526(419-656)	169(135-211)
**Gabon**	2546(1963-3281)	347(268-447)	811(625-1045)
**Gambia**	491(369-648)	562(422-742)	129(97-170)
**Ghana**	11838(9042-15372)	506(387-657)	274(210-356)
**Guinea**	4336(3315-5624)	494(377-640)	213(163-277)
**Guinea-Bissau**	325(250-420)	496(381-642)	113(87-146)
**Kenya**	19611(16129-23708)	408(336-493)	258(212-312)
**Lesotho**	553(425-714)	753(579-973)	225(173-290)
**Madagascar**	2964(2264-3847)	510(390-662)	70(53-91)
**Mali**	3228(2479-4170)	374(287-483)	98(75-126)
**Mauritania**	1405(1085-1806)	322(249-414)	198(153-255)
**Mauritius**	1175(915-1500)	339(264-432)	943(734-1203)
**Mozambique**	3737(2879-4824)	403(310-520)	75(58-96)
**Namibia**	1785(1390-2278)	404(315-516)	529(412-675)
**Niger**	2568(1968-3328)	348(267-451)	56(43-73)
**Nigeria**	70338(56597-86814)	380(305-468)	224(180-277)
**Rwanda**	4223(3220-5494)	726(553-944)	225(171-292)
**Senegal**	3962(3063-5092)	316(244-406)	153(119-197)
**Sierra Leone**	618(476-797)	450(346-580)	57(44-73)
**South Africa**	59393(49420-70987)	529(440-632)	851(708-1017)
**Sudan**	6403(4825-8418)	541(407-711)	99(74-130)
**Togo**	2314(1771-2998)	732(560-949)	189(144-244)
**Uganda**	16785(12765-21899)	738(561-963)	237(180-310)
**United Republic of Tanzania**	17543(13446-22707)	510(391-661)	180(138-233)

**Zambia**	6254(4838-8025)	502(389-644)	210(163-270)
**Zimbabwe**	3601(2750-4672)	355(271-461)	180(137-233)

Table S18. Total economic cost, cost as a percentage of GDP in 2025–2050, and per capita cost attributable to depression morbidity, by World Bank region and World Bank income group, and globally in billions of 2024 U.S.$ with an upper bound and lower bound. The treatment costs input is half of baseline treatment cost data.

Group	Economic costs, 2024 U.S.$ billion	Proportion of total GDP ($\times 10^{-5}$), 2025 – 2050	Per capita cost, 2024 U.S.$
**By region**			
**East Asia and Pacific**	2925(2434-3499)	338(282-405)	1216(1012-1454)
**Europe and Central Asia**	2988(2401-3699)	488(392-604)	3234(2599-4004)
**Latin America and Caribbean**	548(448-668)	339(277-413)	770(629-938)
**Middle East and North Africa**	397(308-509)	357(277-458)	765(594-981)
**North America**	4054(3443-4757)	559(475-656)	10025(8515-11764)
**South Asia**	588(479-720)	384(312-470)	281(228-343)
**Sub-Saharan Africa**	368(290-465)	484(382-611)	225(177-283)
**By income group**			
**Low income**	107(82-138)	558(430-719)	132(102-171)
**Lower middle income**	1190(953-1478)	370(296-459)	296(237-368)
**Upper middle income**	3098(2571-3715)	359(298-430)	1176(976-1411)
**High income**	7457(6185-8963)	499(414-600)	6188(5132-7438)
**Total**	11869(9804-14316)	439(363-530)	1364(1127-1645)

Economic burden accounting for suicide-related deaths

Tables S19-S22 show the total economic burden of depression in 2025–2050 for each country incorporating sensitivity analyses acoounting for suicide-related deaths. For **Tables S19–S20**, we present the worst-case scenario, assuming that 60% of individuals who die by suicide do so as a result of depression. In contrast, for **Tables S21–S22**, we show a more optimistic scenario, assuming that 16% of suicide deaths are attributable to depression.

Table S19. Total macroeconomic cost, economic cost as a percentage of GDP in 2025–2050, and per capita cost attributable to depression, by country and World Bank region in 2024 U.S. $ accounting for 60% of suicide-related deaths with lower and upper bound, discounting at 2%.

	Economic cost, 2024 U.S.$ million	Proportion of total GDP ($\times 10^{-5}$), 2025 − 2050	Per capita cost, 2024 U.S.$
**East Asia and Pacific**			
**Australia**	289339(229681-364068)	633(503-797)	9663(7671-12159)
**Brunei Darussalam**	899(677-1189)	213(161-282)	1867(1406-2469)
**Cambodia**	4145(3177-5424)	387(296-506)	207(159-271)
**China**	2214512(1851416-2643152)	393(328-469)	1531(1280-1827)
**Fiji**	507(381-677)	343(258-458)	503(378-672)
**Indonesia**	108458(88981-131804)	251(206-305)	347(285-422)
**Japan**	347743(289126-418142)	364(302-437)	3020(2511-3632)
**Lao People's Democratic Republic**	1935(1452-2581)	364(273-485)	221(166-295)
**Malaysia**	53789(41933-68833)	420(327-537)	1419(1106-1815)
**Mongolia**	2702(2060-3569)	478(364-631)	677(516-894)
**Myanmar**	7133(5344-9512)	299(224-398)	118(89-158)
**New Zealand**	31532(25732-38619)	518(423-634)	5892(4808-7216)
**Philippines**	34714(28618-42102)	253(209-307)	263(217-319)
**Republic of Korea**	126318(98862-162745)	316(247-407)	2535(1984-3266)
**Singapore**	24679(19996-30437)	202(164-249)	3882(3146-4788)
**Thailand**	36574(28059-47776)	302(231-394)	530(407-692)
**Viet Nam**	48734(36631-64983)	361(272-482)	458(344-610)
**Europe and Central Asia**			
**Albania**	1603(1233-2081)	330(254-428)	602(463-782)

**Armenia**	2710(2125-3455)	440(345-561)	930(729-1185)
**Austria**	49305(39567-61303)	455(365-565)	5371(4310-6678)
**Azerbaijan**	7923(6129-10215)	377(292-486)	726(561-935)
**Belarus**	11684(8955-15277)	627(481-820)	1295(993-1694)
**Belgium**	86970(69135-109060)	609(484-763)	7220(5740-9054)
**Bosnia and Herzegovina**	2336(1779-3075)	392(299-517)	787(600-1036)
**Bulgaria**	8875(6969-11313)	396(311-504)	1475(1158-1880)
**Croatia**	8531(6638-10928)	494(384-632)	2313(1800-2963)
**Cyprus**	3343(2608-4283)	348(272-446)	2555(1993-3274)
**Czechia**	29450(23182-37407)	412(325-524)	2763(2175-3510)
**Denmark**	54904(43299-69393)	559(441-707)	9000(7098-11375)
**Estonia**	5104(3977-6545)	572(446-733)	4137(3223-5304)
**Finland**	43764(35416-53946)	635(514-783)	7884(6380-9718)
**France**	424962(335864-535780)	637(503-803)	6324(4998-7973)
**Georgia**	3619(2816-4632)	495(386-634)	970(755-1242)
**Germany**	509071(406120-636658)	543(433-679)	6197(4944-7750)
**Greece**	27083(20421-35733)	564(425-744)	2820(2126-3721)
**Hungary**	20118(15565-25977)	464(359-600)	2233(1727-2883)
**Iceland**	3666(2854-4683)	554(432-708)	9978(7769-12745)
**Ireland**	68784(56304-83990)	500(410-611)	12691(10389-15497)
**Italy**	208793(169608-256003)	459(373-563)	3632(2951-4454)
**Kazakhstan**	26847(20719-34920)	394(304-512)	1224(945-1592)
**Kyrgyzstan**	1714(1316-2233)	473(363-616)	212(163-276)
**Latvia**	5631(4414-7183)	608(477-776)	3468(2719-4424)
**Lithuania**	8460(6512-11023)	563(433-733)	3614(2782-4709)
**Luxembourg**	8713(7034-10753)	434(351-536)	11947(9646-14745)
**Montenegro**	677(514-893)	425(323-561)	1106(840-1458)

**Netherlands**	124705(98205-158026)	542(427-687)	7168(5645-9083)
**North Macedonia**	1215(933-1583)	359(276-468)	613(471-799)
**Norway**	79186(64378-97215)	521(424-640)	12856(10451-15783)
**Poland**	67346(53731-84732)	387(309-487)	1890(1508-2378)
**Portugal**	35065(27181-45021)	612(475-786)	3643(2824-4677)
**Republic of Moldova**	3059(2352-3988)	568(437-741)	828(637-1080)
**Romania**	23914(18604-30798)	319(248-411)	1363(1060-1755)
**Russian Federation**	341795(267460-438548)	620(485-795)	2437(1907-3127)
**Serbia**	9049(7010-11692)	443(343-572)	1158(897-1496)
**Slovakia**	11821(9092-15392)	420(323-546)	2251(1731-2931)
**Slovenia**	7464(5838-9520)	528(413-673)	3704(2897-4724)
**Spain**	208360(176055-246008)	644(544-760)	4592(3880-5421)
**Sweden**	92628(76650-111838)	632(523-764)	8490(7026-10251)
**Switzerland**	123058(100916-149669)	628(515-764)	13038(10692-15857)
**Tajikistan**	975(753-1267)	256(198-332)	73(57-95)
**Turkey**	97027(74845-125384)	374(289-484)	1048(808-1354)
**Ukraine**	27259(20627-36489)	618(467-827)	704(532-942)
**United Kingdom**	513252(416374-630601)	647(525-795)	7137(5790-8769)
**Uzbekistan**	11268(8573-14875)	448(341-591)	284(216-375)
**Latin America and Caribbean**			
**Argentina**	49571(40333-60904)	292(237-358)	965(785-1186)
**Bahamas**	1373(1053-1786)	402(308-522)	3103(2379-4035)
**Barbados**	785(599-1024)	526(401-685)	2745(2094-3580)
**Belize**	421(323-547)	443(340-576)	827(635-1074)

**Bolivia (Plurinational State of)**	5834(4465-7656)	447(342-587)	409(313-537)
**Brazil**	244027(204849-290095)	479(402-569)	1076(903-1279)
**Chile**	30822(24237-39182)	407(320-517)	1548(1217-1968)
**Colombia**	29942(23603-37930)	331(261-419)	548(432-695)
**Costa Rica**	9993(7602-13106)	506(385-663)	1782(1355-2337)
**Dominican Republic**	19107(14322-25466)	547(410-729)	1565(1173-2086)
**Ecuador**	12228(9410-15931)	360(277-468)	577(444-751)
**El Salvador**	4359(3273-5844)	477(358-639)	635(477-851)
**Guatemala**	10649(8165-13902)	324(249-423)	454(348-593)
**Haiti**	2561(1890-3500)	461(340-629)	189(139-258)
**Honduras**	4139(3119-5508)	407(307-542)	334(252-444)
**Jamaica**	1831(1401-2383)	396(303-515)	604(462-786)
**Mexico**	145539(118809-178469)	342(280-420)	991(809-1215)
**Nicaragua**	2026(1543-2665)	437(332-574)	258(196-339)
**Panama**	7624(6005-9731)	302(238-385)	1440(1134-1838)
**Paraguay**	5752(4402-7548)	416(319-546)	684(523-897)
**Peru**	11144(8809-14171)	167(132-212)	295(233-375)
**Suriname**	639(472-869)	699(517-951)	978(723-1331)
**Uruguay**	7940(6213-10133)	410(321-524)	2204(1724-2812)
**Venezuela (Bolivarian Republic of)**	20544(15241-27773)	467(346-631)	586(435-793)
**Middle East and North Africa**			
**Algeria**	30063(22711-39687)	505(382-667)	554(418-731)
**Bahrain**	4611(3627-5868)	311(245-396)	2158(1697-2746)
**Djibouti**	629(476-836)	547(413-726)	530(400-704)
**Egypt**	49239(38531-62862)	302(236-385)	363(284-464)

**Iran (Islamic Republic of)**	62427(48263-80325)	639(494-823)	645(498-830)
**Iraq**	23442(18421-30089)	278(218-356)	404(318-519)
**Israel**	84146(66301-106637)	549(432-695)	7648(6026-9692)
**Jordan**	4902(3809-6292)	376(292-482)	424(329-544)
**Kuwait**	11161(9034-13860)	260(210-322)	2222(1798-2759)
**Lebanon**	3430(2568-4541)	521(390-690)	540(405-716)
**Malta**	1741(1371-2211)	420(330-533)	3948(3108-5012)
**Morocco**	21938(16833-28591)	521(399-678)	510(391-664)
**Oman**	20341(15570-26438)	535(410-696)	3230(2472-4198)
**Qatar**	14315(11410-18023)	203(161-255)	4047(3226-5095)
**Saudi Arabia**	93945(73645-121029)	314(246-405)	2265(1775-2917)
**Syrian Arab Republic**	3962(2996-5204)	471(356-619)	136(103-179)
**Tunisia**	7130(5419-9368)	555(422-729)	541(411-711)
**North America**			
**Canada**	291999(234691-362356)	531(427-660)	6833(5492-8480)
**United States of America**	4638755(3977387-5402291)	693(594-807)	12828(10999-14939)
**South Asia**			
**Bangladesh**	85487(65092-112055)	581(442-761)	462(352-605)
**Bhutan**	499(378-659)	540(409-713)	573(435-757)
**India**	533873(431060-660629)	438(353-542)	342(276-423)
**Maldives**	798(625-1017)	356(279-454)	1456(1140-1856)
**Nepal**	8986(6796-11974)	676(511-900)	262(198-349)
**Pakistan**	64304(48819-85592)	495(376-659)	220(167-293)
**Sri Lanka**	8624(6459-11568)	456(341-611)	391(293-525)
**Sub-Saharan Africa**			
**Angola**	38396(28652-51272)	762(569-1018)	679(507-906)

**Benin**	3914(2945-5198)	553(416-735)	208(156-276)
**Botswana**	2098(1647-2703)	316(248-407)	685(538-883)
**Burkina Faso**	4146(3157-5438)	535(407-702)	125(95-163)
**Burundi**	1014(766-1342)	594(449-786)	53(40-70)
**Côte d'Ivoire**	11531(8758-15184)	403(306-530)	287(218-378)
**Cabo Verde**	510(389-668)	687(525-901)	798(609-1045)
**Cameroon**	8795(6640-11664)	494(373-655)	220(166-292)
**Central African Republic**	207(162-268)	266(208-344)	30(24-39)
**Chad**	3206(2390-4303)	607(452-814)	122(91-164)
**Comoros**	235(179-311)	506(384-669)	193(147-256)
**Congo**	4264(3204-5669)	768(577-1021)	509(383-677)
**Democratic Republic of the Congo**	19893(14770-26791)	737(548-993)	135(100-182)
**Eswatini**	1150(843-1586)	685(502-944)	790(579-1089)
**Ethiopia**	30375(24009-38258)	563(445-709)	181(143-228)
**Gabon**	2820(2159-3697)	384(294-504)	898(688-1178)
**Gambia**	521(386-705)	596(441-807)	137(101-185)
**Ghana**	12761(9632-16851)	546(412-720)	296(223-391)
**Guinea**	4585(3450-6094)	522(393-694)	226(170-300)
**Guinea-Bissau**	367(275-491)	560(420-750)	128(96-171)
**Kenya**	21146(17263-25863)	440(359-538)	278(227-340)
**Lesotho**	656(487-892)	894(663-1215)	267(198-363)
**Madagascar**	3190(2402-4228)	549(414-728)	75(57-100)
**Mali**	3459(2617-4585)	400(303-531)	105(79-139)
**Mauritania**	1508(1152-1982)	346(264-454)	213(162-279)
**Mauritius**	1359(1056-1744)	392(304-503)	1091(847-1400)
**Mozambique**	4313(3279-5712)	465(354-616)	86(66-114)

**Namibia**	2107(1589-2829)	477(360-641)	624(471-838)
**Niger**	2760(2101-3641)	374(285-494)	60(46-79)
**Nigeria**	75302(59971-94396)	406(324-509)	240(191-301)
**Rwanda**	4506(3392-5975)	774(583-1027)	240(181-318)
**Senegal**	4416(3378-5779)	352(270-461)	171(131-224)
**Sierra Leone**	661(503-872)	481(366-635)	61(46-80)
**South Africa**	70595(57620-86410)	629(513-770)	1012(826-1238)
**Sudan**	6858(5071-9276)	579(428-783)	106(78-143)
**Togo**	2531(1893-3380)	801(599-1070)	206(154-275)
**Uganda**	17895(13516-23611)	787(594-1038)	253(191-334)
**United Republic of Tanzania**	18904(14319-24889)	550(417-724)	194(147-256)
**Zambia**	7018(5324-9266)	564(428-744)	236(179-312)
**Zimbabwe**	4617(3284-6578)	455(324-649)	230(164-328)

Table S20. Total economic cost, cost as a percentage of GDP in 2025–2050, and per capita cost attributable to depression morbidity, by World Bank region and World Bank income group, and globally in billions of 2024 U.S.$ accounting for 60% of suicide-related deaths with an upper bound and lower bound.

	**Economic costs, 2024 U.S.$ billion**	**Proportion of total GDP ($\times10^{-5}$), 2025 – 2050**	**Per capita cost, 2024 U.S.$**
**By region**			
**East Asia and Pacific**	3334(2752-4036)	386(318-467)	1386(1144-1677)
**Europe and Central Asia**	3413(2731-4261)	557(446-695)	3694(2956-4613)
**Latin America and Caribbean**	629(510-776)	389(316-480)	883(717-1090)
**Middle East and North Africa**	437(341-562)	393(307-505)	843(657-1083)

**North America**	4931(4212-5765)	680(581-795)	12194(10417-14257)
**South Asia**	703(559-883)	459(365-577)	335(267-421)
**Sub-Saharan Africa**	405(315-520)	532(414-684)	247(192-317)
**By income group**			
**Low income**	114(87-150)	599(456-785)	142(108-186)
**Lower middle income**	1357(1069-1723)	421(332-535)	337(266-428)
**Upper middle income**	3507(2882-4268)	406(334-494)	1332(1094-1621)
**High income**	8852(7366-10635)	592(493-711)	7346(6113-8825)
**Total**	13851(11420-16804)	512(422-622)	1592(1312-1931)

Table S21. Total macroeconomic cost, economic cost as a percentage of GDP in 2025–2050, and per capita cost attributable to depression, by country and World Bank region in 2024 U.S. $ accounting for 16% of suicide-related deaths with lower and upper bound, discounting at 2%.

	Economic cost, 2024 U.S.$ million	Proportion of total GDP ($\times 10^{-5}$), 2025 − 2050	Per capita cost, 2024 U.S.$
**East Asia and Pacific**			
**Australia**	267516(213906-334056)	586(468-731)	8935(7144-11157)
**Brunei Darussalam**	837(639-1087)	199(152-258)	1737(1328-2257)
**Cambodia**	3943(3069-5053)	368(286-471)	197(153-252)
**China**	2110067(1778547-2494634)	374(315-442)	1458(1229-1724)
**Fiji**	451(349-581)	305(236-393)	447(346-576)
**Indonesia**	106665(87920-128798)	247(203-298)	341(281-412)
**Japan**	296947(251552-349724)	310(263-366)	2579(2185-3037)
**Lao People's Democratic Republic**	1816(1388-2363)	341(261-444)	208(159-270)
**Malaysia**	51587(40613-65214)	403(317-509)	1360(1071-1720)

**Mongolia**	2367(1861-3010)	418(329-532)	593(466-754)
**Myanmar**	6717(5110-8785)	281(214-368)	112(85-146)
**New Zealand**	29022(23935-35133)	477(393-577)	5423(4473-6565)
**Philippines**	33072(27523-39660)	241(201-289)	250(208-300)
**Republic of Korea**	103583(84352-127468)	259(211-319)	2079(1693-2558)
**Singapore**	22851(18694-27886)	187(153-228)	3595(2941-4387)
**Thailand**	32648(25635-41510)	269(211-342)	473(371-601)
**Viet Nam**	44686(34349-57928)	331(255-429)	420(323-544)
**Europe and Central Asia**			
**Albania**	1545(1201-1977)	318(247-407)	580(451-743)
**Armenia**	2603(2057-3286)	423(334-533)	893(706-1127)
**Austria**	45988(37123-56827)	424(342-524)	5010(4044-6190)
**Azerbaijan**	7754(6035-9914)	369(287-472)	710(553-908)
**Belarus**	10395(8159-13208)	558(438-709)	1152(905-1464)
**Belgium**	79300(63565-98564)	555(445-690)	6583(5277-8183)
**Bosnia and Herzegovina**	2169(1683-2788)	364(283-468)	731(567-940)
**Bulgaria**	8150(6502-10198)	363(290-455)	1354(1080-1695)
**Croatia**	8004(6295-10122)	463(364-586)	2170(1707-2745)
**Cyprus**	3260(2556-4150)	340(266-432)	2491(1954-3172)
**Czechia**	27204(21700-34032)	381(304-476)	2552(2036-3193)
**Denmark**	52277(41384-65811)	532(421-670)	8570(6784-10788)
**Estonia**	4757(3754-6007)	533(421-673)	3855(3043-4868)
**Finland**	40682(33188-49710)	590(481-721)	7329(5979-8955)
**France**	399265(317268-500523)	598(475-750)	5942(4721-7449)
**Georgia**	3424(2689-4337)	469(368-594)	918(721-1163)
**Germany**	479079(384297-595736)	511(410-635)	5832(4678-7252)
**Greece**	26544(20038-34979)	553(417-729)	2764(2087-3642)

**Hungary**	18390(14459-23297)	424(334-538)	2041(1605-2586)
**Iceland**	3327(2610-4216)	503(395-638)	9057(7105-11476)
**Ireland**	65567(54034-79467)	477(393-578)	12098(9970-14662)
**Italy**	202301(164755-247364)	445(362-544)	3519(2866-4303)
**Kazakhstan**	23074(18311-29040)	338(269-426)	1052(835-1324)
**Kyrgyzstan**	1540(1209-1954)	425(333-539)	190(150-242)
**Latvia**	5101(4072-6369)	551(440-688)	3142(2508-3922)
**Lithuania**	7434(5851-9437)	494(389-628)	3175(2499-4031)
**Luxembourg**	8367(6793-10263)	417(339-512)	11474(9314-14073)
**Montenegro**	609(473-781)	382(297-491)	995(772-1276)
**Netherlands**	117152(92779-147581)	509(403-641)	6734(5333-8483)
**North Macedonia**	1150(896-1471)	340(265-435)	580(452-742)
**Norway**	73897(60457-90117)	486(398-593)	11997(9815-14630)
**Poland**	58336(47862-70973)	335(275-408)	1638(1344-1992)
**Portugal**	33890(26349-43376)	592(460-758)	3521(2737-4506)
**Republic of Moldova**	2789(2188-3544)	518(406-658)	755(592-959)
**Romania**	22019(17427-27774)	294(233-371)	1255(993-1583)
**Russian Federation**	287610(232468-355184)	521(421-644)	2050(1657-2532)
**Serbia**	8308(6558-10495)	407(321-514)	1063(839-1343)
**Slovakia**	10821(8487-13761)	384(301-489)	2060(1616-2620)
**Slovenia**	6979(5525-8776)	494(391-621)	3463(2742-4355)
**Spain**	202681(171967-238175)	626(531-736)	4467(3790-5249)
**Sweden**	87194(72814-104198)	595(497-711)	7992(6674-9551)
**Switzerland**	117611(96935-142264)	601(495-727)	12461(10270-15073)
**Tajikistan**	942(736-1202)	247(193-315)	71(55-90)
**Turkey**	94089(72971-120842)	363(282-466)	1016(788-1305)
**Ukraine**	23462(18523-29732)	532(420-674)	606(478-767)

**United Kingdom**	485190(395519-593016)	612(499-748)	6747(5500-8247)
**Uzbekistan**	10221(7973-13074)	406(317-520)	258(201-330)
**Latin America and Caribbean**			
**Argentina**	44619(36835-53947)	262(217-317)	869(717-1050)
**Bahamas**	1338(1032-1728)	391(302-505)	3024(2332-3905)
**Barbados**	761(585-985)	510(391-659)	2662(2044-3443)
**Belize**	396(308-508)	417(324-535)	778(604-998)
**Bolivia (Plurinational State of)**	5559(4322-7137)	426(331-547)	390(303-501)
**Brazil**	229826(194332-270960)	451(381-532)	1014(857-1195)
**Chile**	28562(22613-36056)	377(298-476)	1435(1136-1811)
**Colombia**	27628(22100-34398)	305(244-380)	506(405-630)
**Costa Rica**	9055(6986-11689)	458(353-591)	1614(1245-2084)
**Dominican Republic**	18324(13904-24026)	524(398-687)	1501(1139-1968)
**Ecuador**	11329(8862-14471)	333(261-426)	534(418-682)
**El Salvador**	3980(3065-5162)	435(335-565)	580(447-752)
**Guatemala**	10010(7758-12909)	305(236-393)	427(331-551)
**Haiti**	2391(1808-3154)	430(325-567)	176(133-232)
**Honduras**	3986(3043-5206)	392(299-512)	322(246-420)
**Jamaica**	1809(1388-2345)	391(300-507)	597(458-774)
**Mexico**	135685(112296-163678)	319(264-385)	924(764-1114)
**Nicaragua**	1908(1476-2460)	411(318-530)	243(188-313)
**Panama**	7249(5774-9128)	287(228-361)	1369(1091-1724)
**Paraguay**	5428(4223-6969)	393(306-504)	645(502-828)
**Peru**	10749(8593-13460)	161(128-201)	284(227-356)
**Suriname**	546(419-711)	597(459-777)	836(642-1088)
**Uruguay**	6787(5390-8529)	351(279-441)	1884(1496-2367)

**Venezuela (Bolivarian Republic of)**	18263(13910-23913)	415(316-543)	521(397-683)
**Middle East and North Africa**			
**Algeria**	29315(22298-38356)	493(375-645)	540(411-706)
**Bahrain**	4491(3552-5677)	303(240-383)	2101(1662-2656)
**Djibouti**	609(467-792)	529(405-688)	513(393-667)
**Egypt**	48616(38163-61818)	298(234-379)	359(282-456)
**Iran (Islamic Republic of)**	60272(46741-77300)	617(479-792)	623(483-798)
**Iraq**	22413(17869-28218)	265(212-334)	386(308-487)
**Israel**	81722(64574-103263)	533(421-673)	7428(5869-9386)
**Jordan**	4841(3773-6191)	371(289-475)	418(326-535)
**Kuwait**	11014(8938-13639)	256(208-317)	2193(1779-2715)
**Lebanon**	3405(2554-4499)	517(388-684)	537(402-709)
**Malta**	1683(1330-2129)	405(320-513)	3816(3015-4827)
**Morocco**	21420(16567-27607)	508(393-655)	498(385-642)
**Oman**	20190(15486-26174)	531(407-689)	3206(2459-4156)
**Qatar**	13993(11216-17497)	198(159-248)	3956(3171-4947)
**Saudi Arabia**	88618(70669-111684)	296(236-373)	2136(1703-2692)
**Syrian Arab Republic**	3924(2975-5136)	467(354-611)	135(102-177)
**Tunisia**	6959(5325-9062)	542(415-705)	528(404-688)
**North America**			
**Canada**	262283(212989-321906)	477(388-586)	6138(4984-7533)
**United States of America**	4234172(3643781-4912682)	632(544-734)	11709(10076-13585)
**South Asia**			
**Bangladesh**	83254(63938-107780)	565(434-732)	450(345-582)

**Bhutan**	479(368-620)	519(399-671)	551(423-713)
**India**	466699(384002-565153)	383(315-463)	299(246-362)
**Maldives**	781(615-989)	349(275-442)	1426(1122-1805)
**Nepal**	8257(6408-10623)	621(482-799)	241(187-310)
**Pakistan**	59698(46539-76457)	460(358-589)	205(160-262)
**Sri Lanka**	7396(5737-9508)	391(303-502)	335(260-431)
**Sub-Saharan Africa**			
**Angola**	36923(27831-48672)	733(553-966)	653(492-860)
**Benin**	3737(2852-4868)	528(403-688)	198(151-259)
**Botswana**	1985(1588-2490)	299(239-375)	648(519-814)
**Burkina Faso**	3974(3060-5138)	513(395-663)	119(92-154)
**Burundi**	969(742-1260)	568(435-738)	50(39-65)
**Côte d'Ivoire**	10929(8427-14101)	382(294-492)	272(210-351)
**Cabo Verde**	478(371-614)	644(501-827)	748(581-960)
**Cameroon**	8339(6396-10825)	468(359-608)	209(160-271)
**Central African Republic**	198(157-251)	254(202-323)	29(23-37)
**Chad**	3075(2323-4050)	582(440-766)	117(89-154)
**Comoros**	227(175-293)	488(376-631)	186(144-241)
**Congo**	4070(3098-5318)	733(558-958)	486(370-635)
**Democratic Republic of the Congo**	19023(14323-25133)	705(531-932)	129(97-170)
**Eswatini**	1014(770-1334)	603(459-794)	696(529-916)
**Ethiopia**	29205(23260-36453)	541(431-675)	174(139-217)
**Gabon**	2698(2094-3471)	368(285-473)	859(667-1106)
**Gambia**	503(377-667)	575(431-763)	132(99-175)
**Ghana**	12194(9307-15880)	521(398-679)	283(216-368)
**Guinea**	4422(3370-5769)	504(384-657)	217(166-284)

**Guinea-Bissau**	342(262-445)	522(400-679)	119(91-155)
**Kenya**	20376(16784-24643)	424(349-513)	268(221-324)
**Lesotho**	589(450-770)	803(613-1049)	240(183-313)
**Madagascar**	3049(2326-3974)	525(400-684)	72(55-94)
**Mali**	3330(2554-4325)	386(296-501)	101(77-131)
**Mauritania**	1458(1128-1880)	334(258-431)	206(159-265)
**Mauritius**	1255(983-1595)	362(283-460)	1007(789-1280)
**Mozambique**	4030(3125-5201)	435(337-561)	81(62-104)
**Namibia**	1920(1492-2475)	435(338-561)	569(442-733)
**Niger**	2680(2064-3472)	363(280-471)	58(45-76)
**Nigeria**	72456(58291-89630)	391(315-484)	231(186-286)
**Rwanda**	4336(3301-5665)	745(567-974)	231(176-301)
**Senegal**	4181(3243-5375)	334(259-429)	162(126-208)
**Sierra Leone**	639(493-826)	465(358-601)	59(45-76)
**South Africa**	63460(52691-76172)	565(469-679)	909(755-1091)
**Sudan**	6553(4917-8679)	553(415-733)	101(76-134)
**Togo**	2378(1810-3106)	753(573-983)	194(147-253)
**Uganda**	17304(13177-22599)	761(579-993)	245(186-320)
**United Republic of Tanzania**	18081(13851-23468)	526(403-683)	186(142-241)
**Zambia**	6557(5066-8456)	527(407-679)	220(170-284)
**Zimbabwe**	3872(2892-5180)	382(285-511)	193(144-259)

Table S22. Total economic cost, cost as a percentage of GDP in 2025–2050, and per capita cost attributable to depression morbidity, by World Bank region and World Bank income group, and globally in billions of 2024 U.S.$ accounting for 16% of suicide-related deaths with an upper bound and lower bound.

	Economic costs, 2024 U.S.$ billion	Proportion of total GDP ($\times10^{-5}$), 2025 − 2050	Per capita cost, 2024 U.S.$

**By region**			
**East Asia and Pacific**	3115(2599-3723)	360(301-431)	1295(1080-1547)
**Europe and Central Asia**	3182(2570-3930)	519(420-641)	3445(2782-4254)
**Latin America and Caribbean**	586(481-714)	363(298-442)	823(676-1002)
**Middle East and North Africa**	423(332-539)	381(299-485)	816(641-1039)
**North America**	4496(3857-5235)	620(532-722)	11120(9538-12946)
**South Asia**	627(508-771)	409(331-503)	299(242-368)
**Sub-Saharan Africa**	383(301-485)	503(396-637)	233(184-295)
**By income group**			
**Low income**	110(85-142)	575(443-743)	136(105-176)
**Lower middle income**	1252(1001-1562)	389(311-485)	311(249-388)
**Upper middle income**	3285(2732-3942)	381(317-457)	1247(1037-1497)
**High income**	8147(6818-9726)	545(456-651)	6761(5658-8072)
**Total**	12813(10649-15396)	474(394-569)	1472(1224-1769)

E: Strengths and limitations

Table S23. Comparison of per-patient economic burden estimates for depression across different methodological approaches.

**Study (Year)**	**Country**	**Method**	**Per-patient cost (2024 USD)**		**Estimate in our study***
Ding et al. [36](2013–2016)	China	COI: direct medical cost	RMB 2,706.92	438 ↓	1,321
Greenberg et al. [37](2005, 2010)	USA	COI: incremental cost	\$5,707 in 2005, \$5,988 in 2010	8,211 –8,614	8,716
Greenberg et al. [38](2019)	USA	Human capital: missed workdays	\$16,854	20,680 ↑	8,716

Iordache et al. [39](2015–2021)	Romania	COI: direct cost + morbidity cost (sick) + productivity loss (death)	EUR 5,553: 143 + 273 + 5,137	7,345 ↑	1,756
Tomonaga et al. [40](2009)	Switzerland	Direct costs+ Indirect costs (disability insurance + workdays lost)	EUR: 23,557: 10,468 + 13,089	35,931↑	8,171

*Comparable estimate in this study was derived by converting macroeconomic losses into per-patient terms using country-specific prevalence rates. These estimates reflect broader economic impacts and are not directly comparable with cost-of-illness measures. ↓ indicates that the cost reported in the previous study was lower than our estimate, where as ↑ indicates that the cost reported in the previous study was higher than our estimate.

Table S23 compares per-patient economic burden estimates from selected studies using different methodological approaches. "Per-patient cost (2024 USD)" refers to estimates reported in the original studies, converted to 2024 USD using World Bank exchange rates and inflation adjustments for comparability. "Estimate in our study" represents the average annual per-patient economic burden over 2025–2050, derived by allocating macroeconomic losses to individuals based on country-specific prevalence rates.

It should be noted that different studies varied substantially in the disease definitions applied and the populations included. Comparisons with the estimates from our study should be interpreted with caution. Ding et al. [36] identified patients with depression using ICD-10 diagnostic codes (F32.0–F32.3, F32.8, F32.9, F33.0–F33.3, F33.8, and F33.9), but their study population was limited to 26,123 urban insured patients with depression in China. Greenberg et al.[37,38] focused primarily on adult major depression, with the study population restricted to U.S. adults aged 18–64 years. Similarly, Iordache et al. [39] identified depression cases based on ICD-10 diagnostic codes (F32.0, F32.1, F32.2, F32.3, F32.8, F32.9, F33.4, F33.8, and F33.9), and their analysis focused on 2,504,792 adults diagnosed with depression and covered by the public health insurance system. While Tomonaga et al. [40] screened patients with depression according to the WHO ICD-10 criteria, and only included 566 outpatients diagnosed with depression. Our estimates capture broader economy-wide impacts and are not directly comparable with conventional cost-of-illness measures. Building on these comparisons, we summarize the key strengths and limitations of our approach relative to existing methods.

Strengths

- Our calculations draw on recently developed methods and integrate the most comprehensive global datasets, including the updated GBD Study 2023, the Barro–Lee education database, the World Bank, and the International Labour Organization.
- Our study represents the first effort to assess the morbidity-driven impact of depression on economic growth across 154 countries and territories.
- Our analytical framework distinguishes productivity losses by education and experience levels, thereby addressing a major limitation in previous work.
- Our model incorporates sex- and age-specific human-capital profiles to reflect how differences in education among cohorts influence productivity losses caused by depression.
- Our approach constructs causal links from depression to GDP by using a simulated production function, which eliminates concerns about reverse causality inherent in observational estimates.
- Our Appendix provides a detailed, step-by-step description of each method, data source, and parameter used in our analysis.

- Our sensitivity analyses adjust morbidity inputs using the upper and lower bounds from GBD data to capture the effect of parameter uncertainty.

Limitations

- We relied on imputations based on U.S. data to estimate health expenditures for depression, which may misestimate actual treatment costs in other countries.

- We applied an average duration for depression across all severity levels and diagnostic categories, which may bias our estimates because recovery times differ by disorder severity and subtype.

References

1. Bloom DE, Chen S, Kuhn M, McGovern ME, Oxley L, Prettner K. The economic burden of chronic diseases: estimates and projections for China, Japan, and South Korea. *The Journal of the Economics of Ageing* 2020; **17**(2020): 100163.
2. Chen S, Kuhn M, Prettner K, Bloom DE. The macroeconomic burden of noncommunicable diseases in the United States: Estimates and projections. *PLOS ONE* 2018; **13**(11): e0206702.
3. Bloom DE, Chen S, Kuhn M, Prettner K. The flip side of "live long and prosper": Noncommunicable diseases in the OECD and their macroeconomic impact. In: Bloom DE, editor. Live Long and Prosper? The Economics of Ageing Populations. London, UK: VoxEU.org and Centre for Economic Policy Research (CEPR); 2019. p. 44.
4. Chen S, Kuhn M, Prettner K, Bloom DE. Noncommunicable diseases attributable to tobacco use in china: macroeconomic burden and tobacco control policies. *Health Affairs* 2019; **38**(11): 1832-9.
5. Chen S, Bloom DE. The macroeconomic burden of noncommunicable diseases associated with air pollution in China. *PLoS One* 2019; **14**(4): e0215663.
6. Chen S, Cao Z, Prettner K, et al. Estimates and Projections of the Global Economic Cost of 29 Cancers in 204 Countries and Territories From 2020 to 2050. *JAMA Oncol* 2023; **9**(4): 465-72.
7. Chen S, Cao Z, Nandi A, et al. The global macroeconomic burden of Alzheimer's disease and other dementias: estimates and projections for 152 countries or territories. *Lancet Glob Health* 2024; **12**(9): e1534-e43.
8. Chen S, Kuhn M, Prettner K, et al. The global economic burden of chronic obstructive pulmonary disease for 204 countries and territories in 2020-50: a health-augmented macroeconomic modelling study. *Lancet Glob Health* 2023; **11**(8): e1183-e93.
9. Chen S, Cao Z, Chen W, et al. The global macroeconomic burden of diabetes mellitus. *Nature Medicine* 2026:32(1): 126-138.
10. Lucas RE. On the mechanics of economic development. *Journal of monetary economics* 1988; **22**(1): 3-42.
11. Solow RM. A contribution to the theory of economic growth. *The Quarterly Journal of Economics* 1956; **70**(1): 65–94.
12. Abegunde D, Stanciole A. An estimation of the economic impact of chronic noncommunicable diseases in selected countries. Geneva, Switzerland: World Health Organization, 2006.
13. Mincer J. Schooling, experience, and earnings. Human Behavior & Social Institutions No. 2. 261 Madison Ave., New York, New York 10016: National Bureau of Economic Research Inc.; 1974.
14. Chen S, Kuhn M, Prettner K, Bloom DE. The global macroeconomic burden of road injuries: estimates and projections for 166 countries. *The Lancet Planetary Health* 2019; **3**(9): e390-e8.
15. Institute for Health Metrics and Evaluation (IHME). GBD Results. Seattle, WA: IHME, University of Washington. 2024. https://vizhub.healthdata.org/gbd-results/ (accessed 1st May 2025).
16. ten Have M, Penninx BWJH, Tuithof M, et al. Duration of major and minor depressive episodes and associated risk indicators in a psychiatric epidemiological cohort study of the general population. *Acta Psychiatrica Scandinavica* 2017; **136**(3): 300-12.
17. Herrman H, Patel V, Kieling C, et al. Time for united action on depression: a Lancet–World Psychiatric Association Commission. *The Lancet* 2022; **399**(10328): 957-1022.
18. Barro RJ, Lee JW. A new data set of educational attainment in the world, 1950–2010. *Journal of Development Economics* 2013; **104**(September 2013): 184–98.
19. GBD 2021 Forecasting Collaborators. Burden of disease scenarios for 204 countries and territories, 2022-2050: a forecasting analysis for the Global Burden of Disease Study 2021. *Lancet* 2024; **403**(10440): 2204-56.
20. Vos T, Lim SS, Abbafati C, et al. Global burden of 369 diseases and injuries in 204 countries and territories, 1990-2019: a systematic analysis for the Global Burden of Disease Study 2019. *The Lancet* 2020; **396**(10258): 1204-22.
21. Global Burden of Disease Collaborative Network. Gross Domestic Product Per Capita 1960-2050 - FGH 2023. Seattle, United States of America: Institute for Health Metrics and Evaluation (IHME). 2024. https://ghdx.healthdata.org/record/ihme-data/global-gdp-per-capita-1960-2050-fgh-2023 (accessed 1st October 2025).
22. Feenstra RC, Robert Inklaar and Marcel P. Timmer. The Next Generation of the Penn World Table. *American Economic Review* 2015; **105**(10): 3150-82.
23. International Labour Organization. Labour force by sex and age (thousands). 2025. http://ilo.org/global/statistics-and-databases/lang--en/index.htm (accessed 25th April 2025).

24. United Nations. 2019 Revision of World Population Prospects. 2019. https://population.un.org/wpp/Download/Standard/CSV/ (accessed 25th April 2025).
25. World Bank. World Bank database, gross savings (% of GDP). 2022. https://data.worldbank.org/indicator/NY.GNS.ICTR.ZS (accessed 25th April 2025).
26. University of Groningen and University of California D. Capital stock at Current Purchasing Power Parities for United States [CKSPPPUSA666NRUG] retrieved from FRED, Federal Reserve Bank of St. Louis. 2022. https://fred.stlouisfed.org/series/CKSPPPUSA666NRUG (accessed 25th April 2025).
27. Grossmann V, Steger T, Trimborn T. Dynamically optimal R&D subsidization. *J Econ Dyn Control* 2013; **37**(3): 516-34.
28. Psacharopoulos G, Patrinos HA. Returns to investment in education: a decennial review of the global literature. *Education Economics* 2018; **26**(5): 445-58.
29. Heckman JJ, Lochner LJ, Todd PE. Earnings functions, rates of return and treatment effects: The Mincer equation and beyond. *Handbook of the Economics of Education* 2006; **1**(2006): 307–458.
30. Dieleman JL, Beauchamp M, Crosby SW, et al. Tracking US Health Care Spending by Health Condition and County. *JAMA* 2025; **333**(12): 1051-61.
31. Bloom DE, Cafiero E, Eva J-L, et al. The Global Economic Burden of Noncommunicable Diseases. Geneva, Switzerland: World Economic Forum, 2011.
32. Ding D, Lawson KD, Kolbe-Alexander TL, et al. The economic burden of physical inactivity: a global analysis of major non-communicable diseases. *Lancet* 2016; 388(10051): 1311-24.
33. Vieta E, Alonso J, Víctor.Pérez S, et al. Epidemiology and costs of depressive disorder in Spain: the EPICO study. *European neuropsychopharmacology : the journal of the European College of Neuropsychopharmacology* 2021; **50**: 93-103.
34. Yamabe K, Liebert R, Flores N, Pashos CL. Health-related quality of life outcomes, economic burden, and associated costs among diagnosed and undiagnosed depression patients in japan. *ClinicoEconomics and Outcomes Research* 2019; **11**: 233-43.
35. Dhodi DK, Sinha SR, Dawer F, Chavan MS. A prospective cross-sectional study to evaluate the economic burden of patients diagnosed with depression in a tertiary care hospital. *International Journal of Basic & Clinical Pharmacology* 2020; **9**(5): 722-5.
36. Ding R, Zhu D, Wang Y, Yong M, Shi X, He P. Medical service utilisation and direct medical cost of depression: a cross-sectional analysis of urban medical claims data from China. *BMJ Open* 2022; **12**(9): e056422.
37. Greenberg PE, Fournier AA, Sisitsky T, Pike CT, Kessler RC. The economic burden of adults with major depressive disorder in the United States (2005 and 2010). *Journal of Clinical Psychiatry* 2015; **76**(2): 155-62.
38. Greenberg P, Chitnis A, Louie D, et al. The Economic Burden of Adults with Major Depressive Disorder in the United States (2019). *Adv Ther* 2023; **40**(10): 4460-79.
39. Iordache MM, Sorici CO, Aivaz KA, et al. Depression in Central and Eastern Europe: How Much It Costs? Cost of Depression in Romania. *Healthcare (Basel)* 2023; **11**(6): 921.
40. Tomonaga Y, Haettenschwiler J, Hatzinger M, et al. The economic burden of depression in Switzerland. *PharmacoEconomics* 2013; **31**(3): 237-50.
