# Supplementary material for: Global economic burden of depression in 154 countries from 2025 to 2050
Source: Nat Med. 2026 Jul 28;32(9):3406–16. doi: 10.1038/s41591-026-04548-7 (PMC13577905; doi:10.1038/s41591-026-04548-7)
Supplement: Supplementary file 2 [file 41591_2026_4548_MOESM2_ESM.pdf]

Corresponding author(s): Simiao Chen

Last updated by author(s): Jun 23, 2026

Reporting Summary

Nature Portfolio wishes to improve the reproducibility of the work that we publish. This form provides structure for consistency and transparency in reporting. For further information on Nature Portfolio policies, see our Editorial Policies and the Editorial Policy Checklist.

Statistics

For all statistical analyses, confirm that the following items are present in the figure legend, table legend, main text, or Methods section.

n/a	Confirmed	
☐	☒	The exact sample size (*n*) for each experimental group/condition, given as a discrete number and unit of measurement
☒	☐	A statement on whether measurements were taken from distinct samples or whether the same sample was measured repeatedly
☒	☐	The statistical test(s) used AND whether they are one- or two-sided<br>*Only common tests should be described solely by name; describe more complex techniques in the Methods section.*
☒	☐	A description of all covariates tested
☒	☐	A description of any assumptions or corrections, such as tests of normality and adjustment for multiple comparisons
☒	☐	A full description of the statistical parameters including central tendency (e.g. means) or other basic estimates (e.g. regression coefficient) AND variation (e.g. standard deviation) or associated estimates of uncertainty (e.g. confidence intervals)
☒	☐	For null hypothesis testing, the test statistic (e.g. *F*, *t*, *r*) with confidence intervals, effect sizes, degrees of freedom and *P* value noted<br>*Give P values as exact values whenever suitable.*
☒	☐	For Bayesian analysis, information on the choice of priors and Markov chain Monte Carlo settings
☒	☐	For hierarchical and complex designs, identification of the appropriate level for tests and full reporting of outcomes
☒	☐	Estimates of effect sizes (e.g. Cohen's *d*, Pearson's *r*), indicating how they were calculated

Our web collection on statistics for biologists contains articles on many of the points above.

Software and code

Policy information about availability of computer code

Data collection	No custom software was used. Analyses were conducted using R version 4.6.0 and Python version 3.13, as described in the Methods section and Codes.
Data analysis	No custom software or algorithms were used for data collection. Data were obtained from publicly available sources as described in the Data section.

For manuscripts utilizing custom algorithms or software that are central to the research but not yet described in published literature, software must be made available to editors and reviewers. We strongly encourage code deposition in a community repository (e.g. GitHub). See the Nature Portfolio guidelines for submitting code & software for further information.

Data

Policy information about availability of data

All manuscripts must include a data availability statement. This statement should provide the following information, where applicable:

- Accession codes, unique identifiers, or web links for publicly available datasets
- A description of any restrictions on data availability
- For clinical datasets or third party data, please ensure that the statement adheres to our policy

All data used in this study were obtained from publicly available repositories and databases, including the Global Burden of Disease (GBD) Study database (https://ghdx.healthdata.org/), the World Bank Open Data platform (https://data.worldbank.org/), the United Nations World Population Prospects database (https://population.un.org/wpp/), the International Labour Organization database (https://ilostat.ilo.org/), and the Barro-Lee Educational Attainment Database (http://

www.barrolee.com/). Detailed descriptions of the data sources, parameter values, and processing procedures are provided in the Methods, Appendix B, and Table S2. No new datasets were generated for this study. Source data are provided with this paper.

Research involving human participants, their data, or biological material

Policy information about studies with human participants or human data. See also policy information about sex, gender (identity/presentation), and sexual orientation and race, ethnicity and racism.

Reporting on sex and gender	This study used aggregated, publicly available secondary datasets and did not involve direct human participant recruitment or individual-level participant data collection. Sex/gender-stratified results are not included in our reporting. Specifically, while sex was incorporated into the study design through the use of age-, sex- or gender-specific epidemiological inputs from the Global Burden of Disease (GBD) study, we did not perform or present separate sex- or gender-stratified analyses. This is because our study focuses on the overall impacts of asthma on the country-level labour force and the broader country-level economy. Ultimately, this is a country-level analysis designed to estimate population-level macroeconomic impacts, rather than individual-level sex or gender differences.
Reporting on race, ethnicity, or other socially relevant groupings	Race and/or ethnicity data are not included in this study. The underlying global data sources used in our analysis do not provide information on race or ethnicity, and these concepts are not included in the analytical framework. This study is a country-level macroeconomic analysis of depressive disorders across 154 countries and territories. We provide population-level estimates and examine differences by World Bank income group and geographic region, rather than disparities among specific racial or ethnic subgroups.
Population characteristics	The study used country-level and age-sex-specific aggregate data from publicly available international databases covering 154 countries and territories.
Recruitment	No participants were recruited because this study used publicly available secondary datasets.
Ethics oversight	Ethics approval was not required because this study used publicly available, de-identified secondary data and did not involve direct human participant recruitment.

Note that full information on the approval of the study protocol must also be provided in the manuscript.

Field-specific reporting

Please select the one below that is the best fit for your research. If you are not sure, read the appropriate sections before making your selection.

☐ Life sciences ☒ Behavioural & social sciences ☐ Ecological, evolutionary & environmental sciences

For a reference copy of the document with all sections, see nature.com/documents/nr-reporting-summary-flat.pdf

Behavioural & social sciences study design

All studies must disclose on these points even when the disclosure is negative.

Study description	This is a quantitative global macroeconomic modelling study that uses publicly available disease burden, demographic, labour, education, and economic data to estimate the projected economic burden of depression across 154 countries and territories from 2025 to 2050. The study quantifies economic impacts by comparing projected GDP under the status quo with a counterfactual scenario in which depression are mitigated.
Research sample	The research sample comprised 154 countries and territories with complete age- and sex-specific population, morbidity, labour, education, and macroeconomic data available for analysis. The study did not recruit individual participants; instead, it used aggregate, de-identified, publicly available datasets covering country-level demographic and health information, including age- and sex-specific depression morbidity, population size, labour force participation, educational attainment, savings rates, and GDP projections.The datasets were obtained from established international sources, including the Global Burden of Disease Study, World Bank, United Nations World Population Prospects, International Labour Organization, and Barro-Lee Educational Attainment Database.
Sampling strategy	The sample represents approximately 96% of the global population, making it broadly representative at the global level, although countries and territories without complete data were excluded. The study sample was chosen to enable comparable cross-country macroeconomic projections of the economic burden of depression from 2025 to 2050 using standardized, publicly available data.
Data collection	Existing aggregate, de-identified data were extracted from publicly available international databases, including Global Burden of Disease Study, World Bank, United Nations World Population Prospects, International Labour Organization, and Barro-Lee Educational Attainment Database. No participants were recruited; therefore, the presence of others and researcher blinding were not applicable, as this was a non-experimental modelling study.
Timing	The study projects the economic burden from 2025 to 2050. The authors accessed the various existing datasets used for the analysis between June 2023 and June 2026.
Data exclusions	Countries and territories without complete data required for the macroeconomic model were excluded based on pre-established data availability criteria. The final analysis included 154 countries and territories with complete datasets; no further data were excluded after applying these criteria.

Non-participation	The analysis included only countries and territories with complete data across all required datasets, including Global Burden of Disease Study depression morbidity data, population data, labour force participation, educational attainment, savings rates, health expenditure, and GDP projections. Countries or territories with incomplete data in any of these datasets were excluded according to pre-established data availability criteria; after this exclusion, 154 countries and territories were included, and no further data were excluded.
Randomization	Participants were not allocated to experimental groups.

Reporting for specific materials, systems and methods

We require information from authors about some types of materials, experimental systems and methods used in many studies. Here, indicate whether each material, system or method listed is relevant to your study. If you are not sure if a list item applies to your research, read the appropriate section before selecting a response.

Materials & experimental systems

n/a	Involved in the study
☒	☐ Antibodies
☒	☐ Eukaryotic cell lines
☒	☐ Palaeontology and archaeology
☒	☐ Animals and other organisms
☒	☐ Clinical data
☒	☐ Dual use research of concern
☒	☐ Plants

Methods

n/a	Involved in the study
☒	☐ ChIP-seq
☒	☐ Flow cytometry
☒	☐ MRI-based neuroimaging

Plants

Seed stocks	n/a
Novel plant genotypes	n/a
Authentication	n/a
